# An ATP‐Mediated Antibiotic β‐Peptide Nanofiber That Kills Multidrug‐Resistant Bacteria via a Multistage Mechanism

**DOI:** 10.1002/advs.202522269

**Published:** 2026-03-19

**Authors:** Sohini Chakraborty, Kamal el Battioui, Dániel Molnár, Bálint Jezsó, Lejla Daruka, Tasvilla Sonallya, Tünde Juhász, Rita Hirmondó, Imola Cs. Szigyártó, Loránd Románszki, Natália Tőkési, Daniel Pinkas, Eszter Házy, Olivér Pavela, Andrea Bodor, Zoltán Varga, István Mándity, Mihály Kovács, Csaba Pál, Kata Horváti, Judit Tóth, Tamás Beke‐Somfai

**Affiliations:** ^1^ Institute of Materials and Environmental Chemistry HUN‐REN Research Centre for Natural Sciences Budapest Hungary; ^2^ Hevesy György Ph.D. School of Chemistry Eötvös Loránd University Budapest Hungary; ^3^ Institute of Molecular Life Sciences HUN‐REN Research Centre for Natural Sciences Budapest Hungary; ^4^ Doctoral School of Biology and Institute of Biology Eötvös Loránd University Budapest Hungary; ^5^ Synthetic and Systems Biology Unit Institute of Biochemistry HUN‐REN Biological Research Centre Szeged Szeged H‐6726 Hungary; ^6^ Cryo‐Electron Microscopy and Tomography Core Facility CEITEC Masarykova University Brno Czech Republic; ^7^ HUN‐REN‐ELTE Motor Pharmacology Research Group Department of Biochemistry Eötvös Loránd University Budapest Hungary; ^8^ ELTE Eötvös Loránd University Institute of Chemistry Analytical and BioNMR Laboratory Budapest Hungary; ^9^ Department of Organic Chemistry Faculty of Pharmacy Semmelweis University Budapest Hungary; ^10^ MTA–HUN‐REN TTK Lendület “Momentum” Peptide‐Based Vaccines Research Group Institute of Materials and Environmental Chemistry HUN‐REN Research Centre for Natural Sciences Budapest Hungary; ^11^ Department of Chemistry, Stella Maris College (Autonomous) University of Madras Chennai Tamil Nadu India; ^12^ HUN‐REN‐ELTE Motor Pharmacology Research Group, Department of Biochemistry Eötvös Loránd University Budapest Hungary; ^13^ Department of Physical Chemistry and Materials Science, Faculty of Chemical Technology and Biotechnology Budapest University of Technology and Economics Budapest Hungary

**Keywords:** antimicrobials, antibacterial mechanism, β‐peptide, multi‐drug resistance, supramolecule

## Abstract

Artificial bioinspired supramolecular assemblies hold great potential to alter modern medicine. Organic tissue‐like nanomaterials built from foldamer peptidomimetics and natural biomolecules could expand our boundaries toward new biocompounds that are able to address global health challenges. Related, antimicrobial resistance against conventional antibiotics motivates search for new compounds with orthogonal mechanisms. Here we designed an antibacterial system where natural adenosine phosphates (APs) trigger supramolecular co‐assemblies from a lipopolysaccharide‐targeting (LPS) antimicrobial β^3^‐peptide (3K). Cryo‐EM and confocal microscopy images on *E. coli* confirmed that real‐time action of 3K‐APs progresses via a multiscale mechanism. Initially, entangled nanofibers capture bacteria resulting in agglutination. Further, individual cells become enwrapped, undergoing a reduction in cell height, as observed by AFM. Finally, in situ cryo‐EM observations, offering insight into their structural state in solution, suggest an association between the presence of extracellular vesicles and loss of cell integrity, which may contribute to cell death. 3K‐ATP was tested on multidrug resistant bacterial strains which adapted to membrane‐targeting antibiotics, where only limited cross‐resistance was observed. Resistant strains modifying their LPS even showed increased sensitivity. Besides induced disassembly by target membranes, degradation could also be reached through enzymatic hydrolysis of APs, altogether resulting in supramolecular bactericides with controllable build‐up and clearance.

## Graphical abstract

The 3K‐AP system forms infinite supramolecular fibrils that undergo controlled degradation into shorter fragments upon enzymatic hydrolysis. Multitechnique imaging reveals a stepwise bacteriolytic mechanism involving bacterial entrapment, disassembly into lethal constructs, and associated extracellular vesicle release, highlighting the dynamic evolution of 3K‐AP assemblies from robust fibrillar supramolecules to active antimicrobial nanostructures.

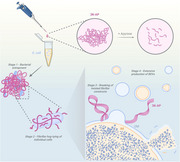

## 1 Introduction

Antimicrobial resistance (AMR) is already a formidable public health threat, however, recent evidence [1, 2] on deaths from multidrug resistant bacterial infections suggests that the current WHO (World Health Organization) predictions need to be remodeled to considerably worse [3, 4]. Gram‐negative multidrug‐resistant bacterial pathogens are particularly lethal [5] and WHO has enlisted several from these species, including *E. coli*, as ‘priority pathogens’ [3]. Considering the shrinking arsenal of effective last resort small molecule antibiotics, there is an urgent need for new antimicrobial systems with novel mechanisms. Optimally, new antibiotics should be able to follow potential adaptations of target microbes, as these could be able to exert orthogonal routes with multiple lines of defence barriers including, for instance, prevention of internal spread. Related, natural stimuli‐activated entrapment mechanisms, such as the formation of neutrophil extracellular traps from antimicrobial enzymes [6, 7, 8], or nanonets, mitigate the diffusion and spread of individual bacteria, facilitating their elimination at the site of infection by an alternative bactericidal mechanism [9, 10]. These traps can be triggered by a diverse set of bacterial species, where at a molecular level, the presence of “danger signals”, such as extracellular ATP and other key compounds, activate inflammatory responses [11, 12]. Several of the above aspects would be highly beneficial in the function of new antimicrobials. In this regard, β‐peptide foldamers and related peptidomimetics have both demonstrated extensive biological applications [13, 14, 15, 16] and high affinity for assembly formation [17, 18, 19, 20]. However, co‐assembly formation of these systems, especially with key biomolecules have not been explored yet.

Here we demonstrate the construction of an artificial supramolecular system using adenosine triphosphate (ATP) and its metabolites with a heterochiral β^3^‐peptide (3K) [21, 22] in order to reach an antimicrobial construct similar to neutrophil extracellular traps. We observed that the 3K‐ATP co‐assembly created an extensive molecular yarn, a fibrous structure with low nanometer width, which exhibited high antibacterial efficacy and negligible hemotoxicity. Its antibiotic mechanism was investigated at different spatio‐temporal scales, using TEM, AFM, cryo‐EM and confocal microscopy. Using these high‐end methods, we demonstrated that the formed supramolecules could wrap around individual *E. coli* cells, eventually leading to massive colony agglutination and a measurable reduction in bacterial diameter along their short axes. Subsequently, time‐dependent cryo‐EM confirmed a spontaneous degradation into shorter fragments, which directly perturb the outer membrane of the bacteria and are associated with the presence of extracellular vesicles (bacterial EVs, BEVs), ultimately leading to the rupture of the bacterial envelope.

## 2 Results

### 2.1 Co‐Assembly of Adenosine Phosphates and 3K

**FIGURE 1:**
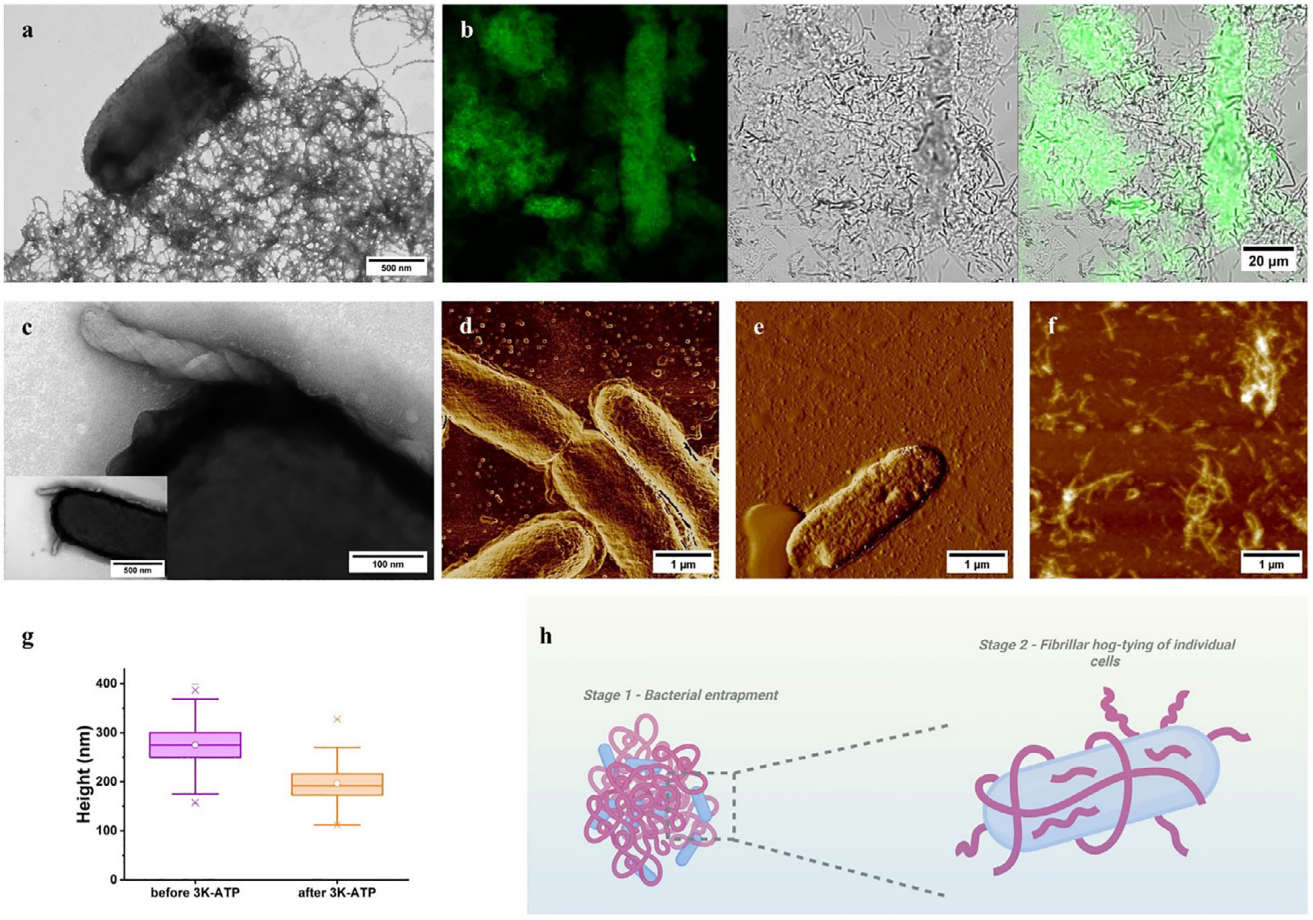
Multitechnique imaging study of the 3K‐AP system showing bacterial entrapment. The 3K‐ATP co‐assembled system generates massive bacterial colony agglutination which is investigated using various imaging techniques. (a) TEM image at 0 min showing bacterial clustering. (b) Confocal microscopy at 60 min revealing continued aggregation. The signal of 3K‐1% fluoATP (fluorescently labelled ATP) is shown in green (first image), *E. coli* are visualized via transmitted light (second column). Merged images are presented in the third column. (c) TEM image at 60 min showing twisted 3K‐ATP fibrils observed on the surface of the bacteria. (d–f) Contrast‐enhanced tapping mode AFM height image of *E. coli* cells tightly surrounded by 3K‐ATP fibrils (d), untreated *E. coli* cells (e), and 3K‐ATP assemblies (f) (3K to AP concentration ratio set at 1:4; 20 µm 3K and 80 µm AP). (g) Average height of *E. coli* cells before and after 3K‐ATP treatment. A reduction of the median cell height by 30% was observed following 3K‐ATP treatment. Three height measurements were taken per cell (38 untreated and 27 treated cells in total). (h) Schematic overview of the first two stages of the multiscale supramolecular antibacterial effect of 3K‐APs illustrated by the imaging techniques shown above: the first stage takes place on the macroscale involving entrapment and bacterial agglutination while the second stage occurs at the single‐cell level, where 3K‐APs enwrap the bacteria. Figure was created with Biorender.com.

**FIGURE 2:**
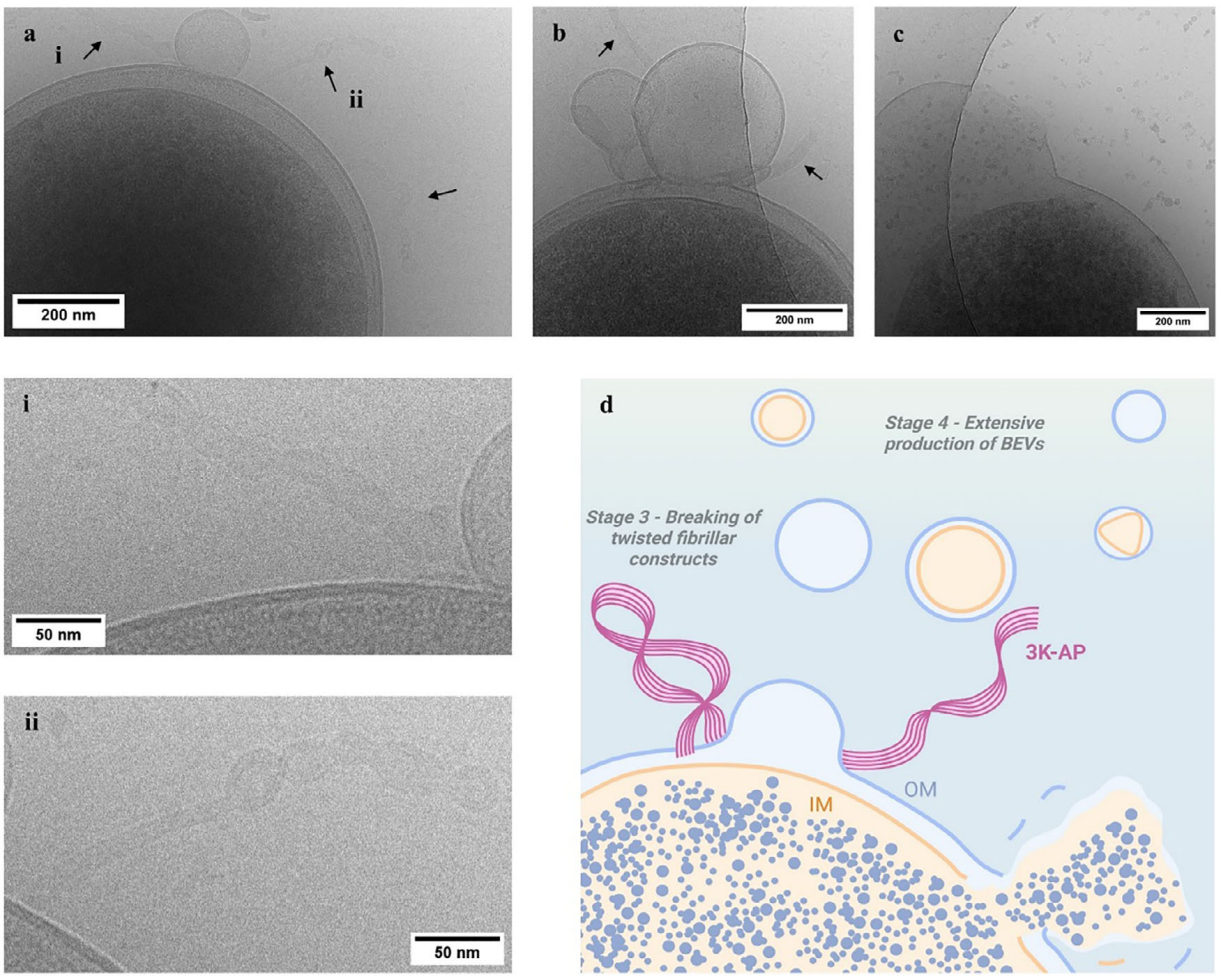
Cryo‐EM images reveal the local antibacterial effect of 3K‐ATP and in situ bacterial extracellular vesicles (BEVs) formation. (a) 3K‐ATP fibrils adopt a coiled coil‐like morphology on the bacterial surface (insets i and ii). (b) Formation of BEVs, including outer membrane vesicles (OMV), is observed at the sites where the coiled‐coil striped fragments of 3K‐ATP interact with the bacterial outer membrane (OM). These finite‐sized, highly coiled, intertwined fibrils are formed nearly perpendicular to the cell wall of the bacteria. (c) The membrane disruption along with the leakage of bacterial contents from the vesicle site indicate the final stage of the 3K‐ATP antibacterial action (the finite‐sized highly coiled 3K‐ATP assemblies and vesicle genesis are presented in detail in Figures S20–S26). (d) Schematic representation of the last two stages of the multiscale antibacterial effect of 3K‐APs: in stage three, at the membrane level, the fibrils become partially disassembled and highly twisted. Finally, the presence of 3K‐AP constructs correlates with membrane perturbation and vesiculation, as well as the appearance of OMVs, outer‐inner membrane vesicles (OIMVs) and irregular OMVs (for more details see *Mechanism of Antibacterial Action* in Discussion). Figure was created with Biorender.com.

The co‐assembly characteristics of 3K with the adenosine phosphates (APs), including ATP, adenosine diphosphate (ADP) and adenosine monophosphate (AMP), were followed using structural assays such as circular dichroism (CD), attenuated total reflectance infrared (IR), and fluorescence spectroscopy [23, 24] and confirmed spontaneous supramolecular assembly of 3K‐AP (Figures S1–S5 and related text) [21, 25, 26, 27]. The interaction between ATP and 3K was also studied by ^1^H and ^31^P NMR methods (Figure S6). In the ^31^P NMR spectra, upon adding 3K to ATP, extensive decrease in all peak intensities corresponding to the phosphate groups of the ATP (α, β and γ) were observed, indicating a high ordered assemblies formation which was no longer visible by NMR and precipitated out from the solution. This effect is similar to that observed for the interaction of 3K with inorganic phosphate solutions [21]. Electron microscopy was used to visualize the formed supramolecular assemblies, and for 3K‐ADP and 3K‐ATP, long fibrous assemblies were obtained (Figure 1a; Figure S7b,c). These twisted fibrils were continuous and infinite, akin to natural actins and amyloids, but the present co‐assemblies were highly entangled and flexible, also maintaining the striped patterns observed earlier for related morphologies (Figure S8) [21]. Analysis of the stripe widths with negative staining TEM (NS‐TEM) yielded an average of 2.3 nm and 2.9 nm for 3K‐ADP and 3K‐ATP, respectively (Figure S9 and Table S1). These morphologies were also re‐affirmed by AFM (Figure 1d–f; Figure S17). Additionally, cryo‐EM also confirmed the formation of the same supramolecular assemblies in solution (Figure 2; Figure S19). Note, that when alone, all APs showed amorphous structures (Figure S7d–f). To gain a deeper understanding of the molecular packing, molecular dynamics (MD) calculations were performed on a short 3K‐ATP segment using our parameter set for β‐amino acids [28, 29] and previous lamellin models [21] (Figure S10 and Table S2). These simulations showed the presence of the double‐array packing of 3K molecules as previously observed [21], but multiple ATP molecules were located between the 3K strands, influencing the macroscopic morphology. The obtained widths between the arrays were ∼2.5 nm, which closely matches the dimensions calculated from the TEM images (Tables S1 and S2).

### 2.2 Antimicrobial Effect on *E. coli* Strains With Limited Cross‐Resistance

**FIGURE 3:**
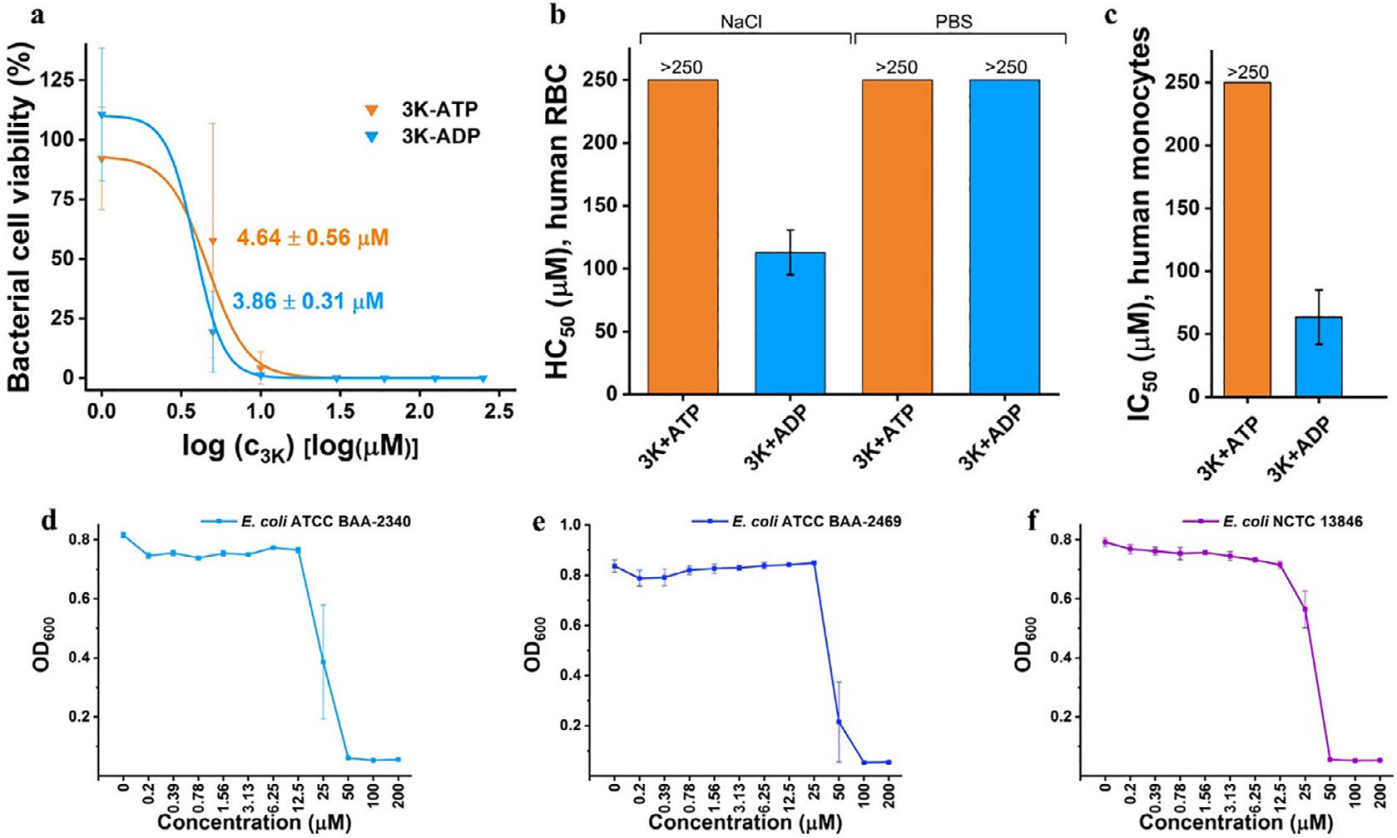
Bioactivity and antibacterial efficacy of the 3K‐AP system. (a) Antibacterial activities of 3K‐ADP and 3K‐ATP in water. Treatments were performed using a fixed nucleotide‐to‐3K molar ratio of 4:1. Viability was defined as the percentage ratio of colony‐forming units (CFU/mL) in 3K‐AP‐treated cultures compared to the respective control cultures. Data represent three biological replicates. IC_50_ values were calculated based on dose‐response fitting of the data points. All errors are reported as SEM (for CFU data, refer to Figure S11). (b) Hemolytic activities of 3K‐ATP and 3K‐ADP in NaCl and PBS media. The half‐maximal hemolysis concentration (HC_50_) values were calculated from the dose‐response curves after fitting with non‐linear regression. All data presented as the mean ± SD (*N* = 4). (c) Cytotoxicity of 3K‐ATP and 3K‐ADP in PBS media measured on MonoMac‐6 human monocytes after 2 h of treatment. The half‐maximal inhibitory concentration (IC_50_) values were calculated from the dose‐response curves after fitting with non‐linear regression. All data presented as the mean ± SD (*N* = 4) (Figure S29 and Table S6). (d–f) Minimum inhibitory concentrations (MICs) of 3K‐ATP (ratio 1:4) were determined against a panel of multidrug‐resistant *Escherichia coli* strains, including *E. coli* ATCC BAA‐2340 (d), *E. coli* ATCC BAA‐2469 (e), and *E. coli NTCT 13846* (f) in LB medium. Bacterial growth was assessed by measuring optical density at 600 nm (OD_600_). For clarity, the graphs are presented with uniformly spaced data points along the *x* axis, regardless of their actual x values. The concentrations on the *x* axis correspond to the 3K concentration within the 3K‐ATP complex.

The antimicrobial efficacy of these structures can be assessed; however, unlike small molecules, it remains challenging to accurately quantify the effects of extended supramolecular assemblies, such as neutrophil traps [30, 31]. Nevertheless, both 3K‐ADP and 3K‐ATP demonstrated potent activity against the laboratory strain *E. coli* BL21 (DE3), with IC_50_ = 4.64 ± 0.56 and 3.86 ± 0.31 µm, respectively (Figure 3a; Figure S11 and Table S3). Additionally, the minimum inhibitory concentration (MIC) of 3K‐ATP was evaluated in a standardized microbiological assay across a panel of pathogenic and multidrug‐resistant *E. coli* strains, showing growth inhibition within the 12.5–100 µm concentration range (Figure 3d–f; Figures S12–S14). To investigate the impact of membrane‐associated mutations on 3K‐ATP susceptibility, we analysed *E. coli* ATCC 25922 strains which were previously adapted to membrane‐targeting antibiotics over ∼120 generations [32]. Notably, while several small molecule antibiotics showed a large increase in MIC values against them [32], with polymyxin B having 128‐fold increase [33], 3K‐ATP showed only a two‐fold increase in MIC, suggesting limited cross‐resistance. Interestingly, tridecaptin M‐adapted lines harboring mutations in the *wbbD* gene showed increased sensitivity to 3K‐ATP. *wbbD* encodes a galactosyltransferase [34] involved in the biosynthesis of the O7‐polysaccharide repeating unit of lipopolysaccharides (LPS), suggesting that alterations in LPS structure may indeed enhance the activity of 3K‐ATP (Figures S12–S14 and related text). Besides activity assays performed under simplified conditions, we evaluated resistant pathogens in LB medium (Figure 3d–f; Figures S13 and S14), which confirmed that 3K‐ATP remains active even in a complex growth medium, consistent with previously reported supramolecular systems [13]. Moreover, the ATP concentrations used were chosen to approximate elevated levels that can occur locally at sites of infection [11, 35, 36].

### 2.3 Multiscale Killing Mechanism With BEV Release Identified in Situ

Most importantly, the mechanism of antibacterial activity for 3K‐APs was investigated in a time‐dependent manner by employing various imaging techniques, including NS‐TEM, AFM, confocal microscopy, and cryo‐EM (Figures S1 and S2). Based on these, several aspects of the antimicrobial effects could be identified (Figure 1h, 2d, 5; Figure S26). At the bacterial colony scale, a mesh of 3K‐AP fibrils surrounding the bacteria could be identified (Figure 1a,b; Figure S15b). This entrapment mechanism was also followed at the macroscale with confocal microscopy, using fluorescently labelled ATP complexed with 3K. At 30 min, evidence of bacterial entrapment was visible, and by 60 min, pockets of the 3K‐ATP mesh were observed entrapping the bacteria in aggregates (Figure 1b; Figure S16). In contrast, APs alone did not build any noticeable mesh, nor induce bacterial aggregation.

When examined at higher resolution on single bacteria, the fibrils were observed gradually folding around the bacteria (Figure 1c; Figures S15c,d,e and S26), where both 3K‐ATP and 3K‐ADP covering their surface, tying‐up individual cells. To gain further insight here, we employed AFM, which confirmed entanglement of individual bacteria along their surface (Figure 1d; Figure S17). In addition, AFM was used to measure the average height of the bacteria under air‐dried conditions. Under these experimental settings, an approximate 30% reduction in bacterial height was observed after treatment, with a statistical significance (Figure 1g). More specifically, the average diameter along the short axis of the individual bacteria decreased from ∼275 to ∼192 nm following treatment with 3K‐ATP whereas non‐treated cells showed no variations from ∼275 nm. While measurements under fully hydrated, near‐native conditions would be more appropriate to assess absolute dimensional changes, the observed differences remain statistically non‐negligeable, indicating a consistent treatment‐associated effect (Figure S18 and Table S4).

At the scale of the cell wall, it can be observed by NS‐TEM, cryo‐EM and AFM that some of these fibrils started to protrude outward and assemble in a more organized manner, undergoing a partial degradation while maintaining a twisted, coiled coil‐like morphology (Figure 1c, 2; Figures S15 g,h, S17f, S21, S24, and S26). The average length of these shorter segments was on the scale of ∼200–500 nm.

As the final key element of the mechanism, on the membrane level, cryo‐EM provided insight into the local antibacterial effect of 3K‐ATP, capturing in situ vesicle formation that may be associated with the lethal effect of the complex (Figure 2; Figures S20, S22, S23, and S26). It is known that dying pyroptotic cells could facilitate the release of extracellular vesicles (EVs) or bacterial EVs (BEVs) [37]. BEVs may carry cellular cargo including cytosolic content, cytoplasmic protein, DNA or RNA, toxins or effectors [38]. However, in this case, BEVs were not only formed independently, but we were also able to directly capture the formation of large BEVs, precisely at the sites where the coiled‐coil striped fragments of 3K‐ATP interacted with the outer membrane (OM) surface of the bacteria (Figure 2; Figures S23 and S26). The end of the 3K‐ATP fragments lies in between the forming BEVs and the OM, suggesting that the supramolecule has a strong amphiphilic character and is able to bend and perturb the bacterial membrane while remaining connected to the surface of both the forming EV and the OM lipid bilayer. To further examine this phenomenon, we have employed model EVs with similar negative surface as for BEVs, and used flow cytometry including our recently established protocol to address EV surface interactions relying on flow linear dichroism spectroscopy (flow‐LD) [39]. Results indicate that upon increasing 3K‐ATP concentrations in the presence of EVs, there is a rapid increase in macroscopic aggregates possibly pointing toward interaction through entrapment and subsequent disruption (Figure s27 and related text) [40, 41]. In overall, the above results confirm a novel antimicrobial mechanism for 3K‐ATP that takes place on multiple scales in a concerted manner (Figure 1h, 2d; Figure S26).

### 2.4 Co‐Assembly Diminishes Hemolytic and Cytotoxic Activities

Subsequently, we explored the hemolytic activity of 3K‐ATP and 3K‐ADP co‐assemblies in two relevant environments, physiological saline solution (SalSol, 0.9% NaCl), and in phosphate buffered saline (PBS). Results indicated that 3K alone had moderate hemolytic activity, with HC_50_ values of 56 and 69 µm, respectively (Figure 2b and Table S5). Interestingly, for 3K‐ATP and 3K‐ADP, significantly higher HC_50_ values were determined (Figure 2b). For 3K‐ATP, HC_50_ values were above 250 µm in both media, whereas for 3K‐ADP, values were 113 µm and above 250 µm, in NaCl and PBS, respectively. Considering that 3K‐ATP exhibited IC_50_ values below 5 µm for *E. coli*, whereas the HC_50_ was more than 50 times higher, there is a significant gap between antibacterial and hemolytic effects, providing an ideal concentration range for therapeutic development (Figure S28 and Table S5).

To further evaluate the biocompatibility of the co‐assemblies, the cytotoxic activity of 3K‐ATP and 3K‐ADP was assessed in PBS using MonoMac‐6 human monocytes. The half‐maximal inhibitory concentration (IC_50_) of 3K‐ADP was determined to be 64 µm, whereas 3K‐ATP exhibited no significant cytotoxicity within the tested concentration range, with IC_50_ values exceeding 250 µm (Figure 3c; Figure S29 and Table S6). Notably, while free 3K displayed measurable cytotoxicity [21], these effects were effectively abolished upon complexation with ATP, indicating that ATP neutralizes the intrinsic cytotoxicity of 3K. Together with the hemolysis data, these results demonstrate a high level of compatibility of 3K‐ATP with mammalian cells, reinforcing the existence of a broad therapeutic window in which potent antibacterial activity is achieved without inducing toxicity toward mammalian cells.

### 2.5 3K‐ATP Controllable Degradation and Stability

**FIGURE 4:**
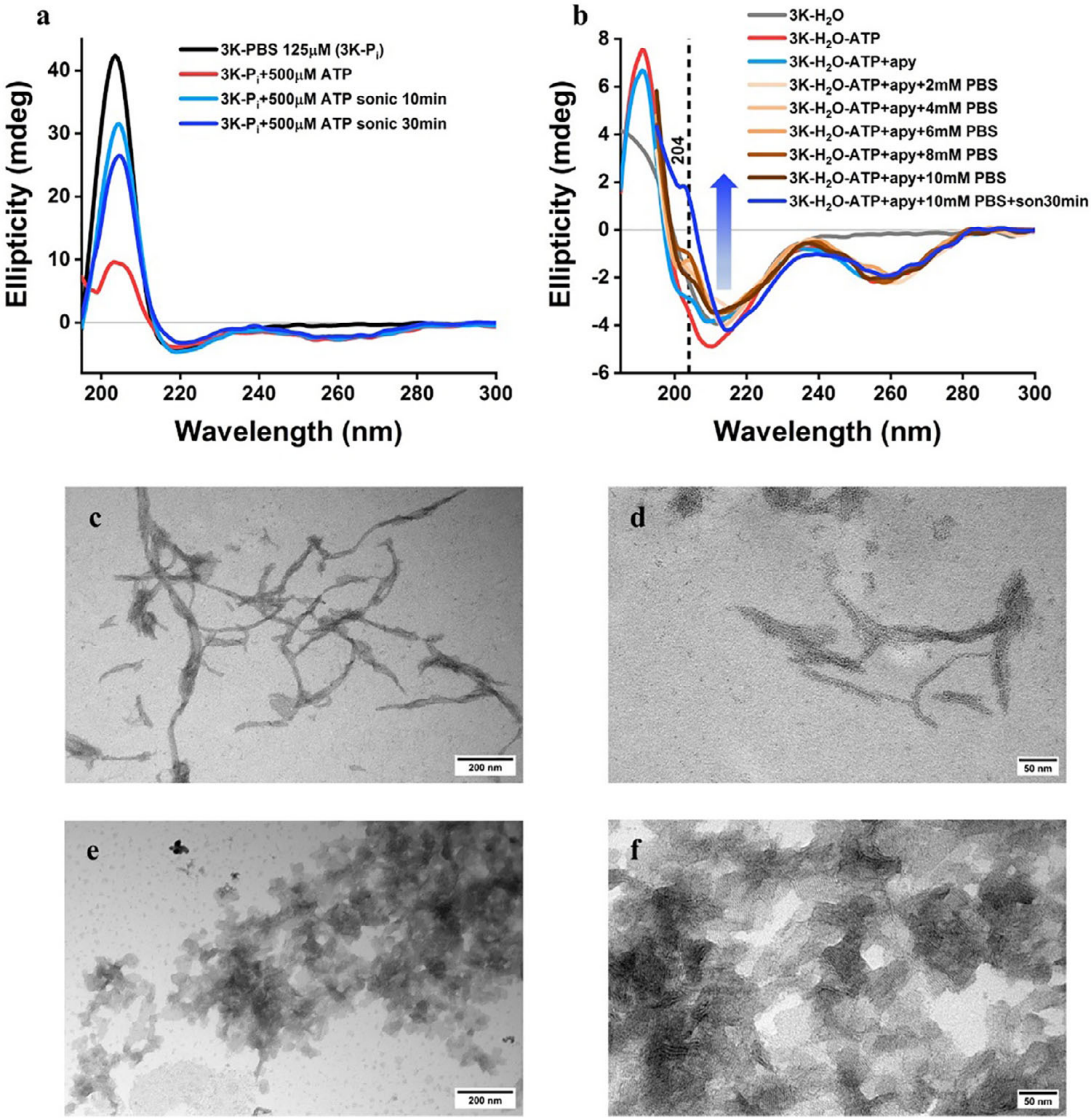
Comparison of the effects of ATP and phosphate ions from PBS upon addition to 3K: degradation and structural stability of the 3K‐ATP assemblies. (a) Upon addition of ATP, the intensity of the characteristic peak of 3K‐PBS (125 µm) reduces drastically. After 10 min sonication, the peak starts building up again, which suggests the presence of both 3K‐PBS and 3K‐ATP populations. (b) In the solution containing 125 µm of 3K and 500 µm of ATP (3K‐H_2_O‐ATP), a calculated amount of apyrase was added to degrade the 500 µm of ATP. An emerging peak at 204 nm is observed, suggesting that apyrase hydrolyses the phosphate group from ATP and the resulting free phosphates co‐assemble with 3K (cyan blue curve). TEM revealed fibrillar morphology that were shorter in length in comparison to those observed in 3K‐ATP assemblies in (c) and (d). To confirm these findings, PBS titration was performed after addition of apyrase, and lamellar morphologies were observed as reported by us earlier for such 3K‐PBS assemblies (lamellin‐3K) in (e) and (f).

Natural extracellular defence networks and ATP‐containing filaments could degrade over time [42], thus, we tested whether 3K‐ATP could spontaneously disassemble through ATP hydrolysis, but the results indicated no such degradation (Figure S30). Further on, we employed apyrase, an enzyme that degrades nucleotide phosphates by cleaving the terminal phosphate groups. Upon addition of apyrase to 3K‐ATP, we observed significantly shorter lamellar morphologies, indicating the disappearance of the longer constructs (Figure 4c–f; Figure S31). These observations show that enzymatic hydrolysis of ATP can modulate and dismantle the 3K‐ATP architecture, serving as a proof‐of‐principle for controllable disassembly and suggesting potential avenues for enzymatically triggered regulation in future applications. Finally, to assess whether 3K‐ATP could be preferred in physiological conditions over other 3K assemblies, we performed several comparison tests in PBS buffer, as we had observed that 3K could form rectangular lamellar structures [21] (3K‐P_i_) with phosphate ions from PBS, which could potentially compete with 3K‐ATP formation. CD spectroscopy results indicated that 3K‐ATP is preferred over 3K‐P_i_ (Figure 4; Figure S31 and related text). Overall, 3K‐ATP are stable under physiological conditions, but they can be enzymatically degraded by targeting ATP, that could result in switching morphology toward smaller lamellae.

## 3 Discussion

### 3.1 Structural Considerations and Supramolecular Assembly Formation

The recently developed acyclic β^3^‐peptides with an alternating chirality pattern in its sequence [22, 43] can change from a random structure into an ordered zig‐zag conformation, which enables assembly formation via interstrand H‐bonds between the peptides. This conformational change was here triggered with APs, that represent multivalent counterions that coordinate to charged side chains in the 3K sequence [21]. Analysis on the morphological details of the 3K‐APs and the MD simulations suggests that their molecular packing is similar to that in striped lamellae of 3K in PBS observed earlier where the double‐arrays of 3K molecules were separated by thin layers of inorganic phosphates (Figures S9,S10 and Tables S1 and S2) [21]. Here, instead of phosphate ions, the larger APs separate probably the double‐arrays of 3K which is supported by the increased stripe size correlating to the size of the AP used. As for similar systems [44], MD simulations suggest that electrostatic interactions drive assembly formation, where multiple APs are present between the peptide arrays forming several interconnected salt‐bridges (Figure S10 and Table S2). The APs in the co‐assembly are more randomly packed, which is likely the reason for formation of flexible, elongated 3K‐AP filaments in contrast to the shorter, rectangular lamellar structures observed in 3K in the presence of small inorganic phosphates [21].

### 3.2 Mechanism of Antibacterial Action

Insight on how antimicrobials exert their function can greatly help in identifying mechanistic differences. Previously, we showed that 3K alone, or in PBS, can form lamellar morphologies upon interaction with LPS on the surface of *E. coli*, that grows into the bacterial membrane and incise that, releasing the inner content and causing bacterial killing almost immediately [21]. In contrast, the antimicrobial effect of 3K‐ATP occurs in several stages and on a longer timescale that enabled a detailed depiction of the concerted killing mechanism through high‐resolution imaging techniques. In overall, we managed to identify distinct phases, which we categorized into four stages along the entire antibacterial process.

**FIGURE 5:**
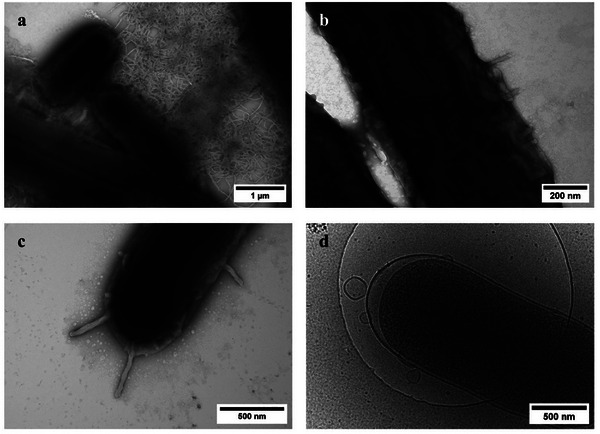
Electron micrographs depicting the multistage antibacterial mechanism of 3K‐AP against *E. coli*. (a) The mechanism starts with the 3K‐ATP fibrils entrapping the bacteria within a mesh‐like network of several hundred nanometers in length, promoting bacterial agglutination. (b) The fibrils gradually fold around the bacteria, ultimately covering their surface and tying the individual cells into finite‐sized constructs. (c) The 3K‐AP fibrils protrude outward, assembling in a more organized manner, and disassemble partially while maintaining a twisted, coiled coil‐like morphology. (d) The formation of extracellular vesicles is observed at the sites where the intertwined fibrils perturb the bacterial outer membrane. Different vesicle types are present including OMVs, OIMVs, and irregular OMVs.

In *Stage 1*, we observed large aggregates where bacterial agglutination is promoted by the 3K‐ATP mesh, a phenomenon that was not observed with 3K alone [21] (Figure 1a, 5a; Figures S15b, S16, and S26). In *Stage 2*, the macroscopic entrapment of bacterial aggregates by the supramolecular assemblies develops at a single bacterial level into “hog‐tying” individual cells (Figure 2, 5b; Figure S26). Besides the cryo‐EM and NS‐TEM images, this aspect is also supported by the reduced height of the individual *E. coli* bacteria measured with AFM after 3K‐ATP treatment (Figure S18 and Table S4). As the process advances, *Stage 2* appears to transition seamlessly into *Stage 3*, suggesting a mechanistic continuum rather than a strictly sequential event. These two stages could occur concomitantly or in concert, where the mechanical stress associated with fibrils entanglement facilitates the detachment of twisted, finite‐length fibrillar segments from the longer filament, which may induce a stronger propensity to interact with the cell wall in comparison to the remaining extended filament form (Figure 1c, 5c, Figures S15e,f and S26) [21]. Here EM images clearly show that numerous finite‐sized highly coiled, intertwined fibrils are formed, with many positioned nearly perpendicular to the cell wall of the bacteria.

Finally, in *Stage 4*, cryo‐EM provides a unique insight directly into the formation of extracellular vesicles on the bacterial outer membrane, exactly at the sites where the intertwined fibrils perturb membrane integrity (Figure 2b,d; Figures S23 and S26). Related, extensive production of BEVs is observed, which is the final stage of antimicrobial mechanism resulting in the cell wall disruption (Figure 5d; Figures S25 and S26). Note, that BEVs are also spontaneously released which may serve as gene transfer, or as a defensive response to hostile environmental changes [45]. However, here the extent is likely lethal, as several bacteria were observed as disrupted in the vicinity of BEVs. We identified four types of BEVs: outer membrane vesicles [45] (OMVs), outer‐inner membrane vesicles [46] (OIMVs), explosive outer membrane vesicles [47] (EOMVs), and irregular outer‐inner membrane vesicles (Figures S20, S23, and S26).

In contrast to 3K alone, which induces membrane incision through interaction with the phosphate groups of lipopolysaccharides (LPS) on the bacterial surface, the 3K‐ATP co‐assemblies operate via a distinct, multistage antibacterial mechanism. This mode of action is characterized by bacterial agglutination and the dissociation of long 3K‐ATP fibrils into shorter fragments that protrude the bacteria, inducing vesicle‐like morphological alteration not observed with 3K alone under comparable conditions. Note that, in contrast to the lamellae observed for 3K alone, which grow into the bacteria [21], neither cryo‐EM nor TEM images of 3K‐ATP samples show similar lamellae structures. This indicates that the antibiotic effect of 3K‐ATP is not influenced by the rapid formation of 3K lamellae. Importantly, ATP also modulates the biological safety profile of 3K: while 3K alone displays measurable cytotoxic effects toward mammalian cells, complexation with ATP markedly attenuates this activity, resulting in minimal cytotoxicity across the tested concentration range and indicating that ATP effectively neutralizes the intrinsic cytotoxicity of the peptide while preserving antibacterial function.

## 4 Conclusion

Here we demonstrated how higher‐order assemblies of peptidic foldamers with small biomolecules could be reached and exploited as alternative strategies that bear the potential to overcome the limitations of existing antimicrobial approaches against multidrug resistant strains. 3K‐APs show a unique mechanism of action on bacteria, that involves bacterial entanglement accompanied by BEV generation, providing an orthogonal mechanistic direction compared to current antibiotics. This is reflected in the results which show only minimal cross resistance for 3K‐ATP on resistant strains adapted to membrane targeting antibiotics, and increased sensitivity for those microbes that deliberately modify LPS during their adaptation. Importantly, although the antibacterial activity of 3K‐ATP is comparable to that of 3K alone, 3K‐ATP has markedly reduced the cytotoxicity, thereby enhancing the balance between antimicrobial potency and host cell compatibility. Combined with the observed negligible hemolytic activity, and controlled degradability, 3K in combination with APs foreshadows future smart antibiotics, which is capable to exert an altered mode of action responding to external signals that could counter adaptation from microbes to bactericidal mechanism of the parent antibiotic. Nanofiber formation via co‐assembly of artificial foldamer peptidomimetics and adenosine phosphates represents the first example of artificial constructs that can mimic natural filaments, which have specific, e.g. antimicrobial function. The current results are expected to lead to new strategies for the design of adaptive, self‐regulating antimicrobial systems that integrate biochemical signalling with supramolecular functionality.

## 5 Experimental Section

### 5.1 Peptide Synthesis

Solid‐phase peptide synthesis of the peptide was carried out in a continuous flow reactor [48]. The coupling reagents and solvents used for the synthesis, such as 1,8‐ diazabicyclo[5.4.0]undec‐7‐ene (DBU), piperidine, trifluoroacetic acid (TFA), N,N‐dimethylformamide (DMF), 1‐[bis(dimethylamino)methylene]‐1H‐1,2,3‐triazolo[4,5‐b]‐pyridinium‐3‐oxide hexafluorophosphate (HATU) and N,N‐diisopropylethylamine (DIPEA) were purchased from Merck Life Science (Budapest, Hungary). TentaGel R RAM resin (capacity = 0.19 mmol/g), purchased from Rapp Polymere GmbH, was loaded into the column (125 × 4 mm). Fmoc‐protected amino acid (2.5 equiv), HATU (2.5 equiv) and DIPEA (5 equiv) were dissolved in 1.5 mL of DMF for the coupling. The flow rate was adjusted to 0.15 mL/min while the pressure and temperature were maintained at 60 bar and 70°C respectively. 2% DBU and 2% piperidine in DMF were used for Fmoc‐deprotection. After coupling and deprotection, the resin was washed with DMF. The peptide was then cleaved by stirring for 3 h in a solution of 95% TFA and 5% water. After evaporation of the TFA, the peptide was precipitated in cold diethyl ether. The crude peptide was purified by reversed‐phase HPLC followed by lyophilization. The peptide was analyzed on an LC‐40 HPLC system (Shimadzu, Kyoto, Japan) on a Phenomenex Jupiter Proteo C12 column (10 µm, 90 Å, 4.6 mm × 150 mm) using gradient elution with eluent A (0.1% TFA in H_2_O) and eluent B (0.1% TFA in ACN: H_2_O = 80:20 (v/v)). The flow rate was 1 mL/min, the gradient was 5–100 B% in 20 min (UV detection at λ = 214 nm). High‐resolution mass spectrometry was performed on a Q Exactive Plus Hybrid Quadrupole‐Orbitrap Mass Spectrometer (Thermo Scientific, Waltham, MA, USA). Monoisotopic molecular mass for 3K [C60H116N12O9] calculated = 1148.8988, observed = 1148.8965 (Figure S32). The measured mass was within 3 ppm of the accurate mass.

### 5.2 Preparation of Peptide Solutions

3 and 3K‐AP were tested in pure MQ water and phosphate buffered saline (PBS). For preparation of the peptide solutions, 125 µm of the peptide and 500 µm of AP were used: H_2_O, pH *6.5*, sonicated 10 min and 10 mm PBS (137 mm NaCl, 2.7 mm KCl, 10 mm of disodium hydrogen phosphate, 2 mm of sodium dihydrogen phosphate), pH *7.4*, sonicated 30 min at 25°C.

As observed in Figures S1 and S3, a plateau is reached at an approximate 1:4 ratio (125 µm 3K to 500 µm nucleotide), which is consistently found for AMP, ADP, and ATP, suggesting that this ratio corresponds to an optimal assembling condition rather than a structural transition. To ensure reproducibility and consistent formation of the assemblies, we therefore adopted this ratio as the standard condition in our experiments.

### 5.3 Circular Dichroism (CD) Spectroscopy

CD spectra were collected at room temperature using a JASCO J‐1500 spectropolarimeter (JASCO, Tokyo, Japan). A rectangular quartz cuvette with a 0.1 cm path length (Hellma, Plainview, NY) was used in continuous mode between 190 to 300 nm with a scanning speed of 50 nm/min, a data pitch of 0.5 nm, response time of 4 s, bandwidth of 2 nm and 3 accumulations. 125 µm of the peptide and 500 µm of AP were used (200 µL). All the spectra were corrected by subtracting a corresponding/matching blank.

### 5.4 Attenuated Total Reflection‐Fourier Transform Infrared (ATR‐FTIR) Spectroscopy

FTIR spectroscopic measurements were performed using a Varian 2000 FTIR Scimitar spectrometer equipped with a Golden Gate accessory (Varian Inc., Palo Alto, CA). 3 µL of the peptides in the aforementioned concentration were pipetted onto the diamond ATR surface and the solvent was evaporated under ambient conditions to obtain a dry film. The spectra were collected at 2 cm^−1^ nominal resolution applying 64 scans. Data acquisition was followed by ATR correction, baseline correction and buffer subtraction. All spectral manipulations were performed using the GRAMS/32 software package (Galactic Inc, USA).

### 5.5 Fluorescence Spectroscopy

Fluorescence spectra were recorded using a JASCO FP‐8500 spectrofluorometer with an excitation and emission bandwidth of 10 and 20 nm, respectively. Three accumulations were measured each time. The 8‐anilino‐1‐naphthalenesulfonic acid (ANS) fluorophore was excited at 388 nm and the emission was recorded from 410 to 600 nm. Binding assays were performed using 125 µm peptide (50 µL) and 2.5 µm ANS. Spectra were corrected for those of the corresponding peptide solutions.

### 5.6 Confocal Microscopy

*E. coli* (BL21 DE3) were grown until exponential growth‐phase as decribed previously [21] and then 20 µL of this homogenous culture was added to 180 µL solution containing 20 µm of 3K, 80 µm of ATP and 0.8 or 0.08 µm of fluorescently labeled ATP (N [6]‐(6‐aminohexyl)‐adenosine‐5'‐triphosphate, labeled with ATTO 550, triethylammonium salt (1 mM) solution in water, NU‐805‐550, Jena Bioscience) in separate wells of an 8 well glass bottom cell culture chamber (Ibidi). Imaging was performed on a Leica SP8 confocal microscopy system using HC PL CS2 APO100x/1.4 OIL objective.

### 5.7 Transmission Electron Microscopy

Two µL of the samples were pipetted in each case to a 200‐mesh copper grid (Ted Pella, Inc, California, USA) with a support film made of formvar. After a contact time of 1 min, excess liquid was removed, and samples were stained with 2% of uranyl acetate. TEM images were obtained routinely at magnifications of 11000x, 28000x, 71000x and 180000x using a Morgagni 268D (FEI, The Netherlands) operating at 80 kV.

### 5.8 Cryo‐Electron Microscopy

5 µL of the samples were applied to freshly plasma‐cleaned TEM grids (Quantifoil, Cu, 300‐mesh, R2/1) and vitrified into liquid ethane using Automatic Plunge Freezer EM GP2 from Leica Microsystems (8°C, 100% rel. humidity, 300 s waiting time, 3.5 s blotting time). The grids were subsequently mounted into the Autogrid cartridges and loaded to Talos Arctica (ThermoScientific) transmission electron microscope for imaging. The microscope was operated at 200 kV. The micrographs were collected on Falcon3 direct electron detection camera at 5600x, 8500x, 49000x, 73000x and 92000x nominal magnifications with an underfocus in the range of 2−3 µm and an overall dose of ∼40 e/Å2.

### 5.9 Atomic Force Microscopy (AFM)

For the AFM imaging and measurements, 1 µL droplets of 3K‐ATP in water (125 µm of 3K and 500 µm of ATP), *E. coli* cells (control), and *E. coli* cells treated with 3K‐ATP (3K:ATP ratio 1:4) for 20 min were deposited onto a cleaned Si (100) wafer chip and let to dry out by evaporation in ambient condition. AFM scans were performed at room temperature, using a Dimenson 3100 AFM instrument equipped with a NanoScope IIIa controller (Digital Instruments/Veeco, USA) in 512 × 512 pixel resolution. Nanosensors TM PPP‐NCHR‐20 type silicon cantilevers with the following parameters, mounted at 10° with respect to the sample stage plane, were used in Tapping Mode: thickness: 40 ± 1 µm; length: 125 ± 10 µm; width: 30 ± 7.5 µm; typical resonance frequency ∼293 kHz; force constant: 10–130 N/m; aluminium‐coated top; tip height: 10–15 µm; typical tip radius <7 nm; tip half cone angle along the cantilever axis: 10°. The scanned areas ranged from 2 × 2 to 50 × 50 µm^2^. The scan rate was 0.1 Hz (= line per second), corresponding to tip velocities ranging from 0.5 to 5 µm/s, depending on the scanned area. Both height and amplitude data were captured. Off‐line treatment of the recorded raw data involved one more of the followings: 1st order plane fit, 0th to 3rd order flattening, “Erase scan lines” treatment, “Edge enhance”, low pass filtering, Gaussian filtering, color contrast and color offset adjustments, application of “Equal area” and “Illuminate” rendition modes.

### 5.10 Hemolytic Activity Assays

Hemolytic activity of the 3K, and 3K‐ATP, 3K‐ADP co‐assemblies was tested on human red blood cells (RBC), as described earlier [49]. Blood of healthy volunteers were obtained from the Hungarian National Blood Transfusion Service (Budapest, Hungary) and centrifuged at 2000 rpm (5 min, 4°C). After the removal of the supernatant, RBCs were washed twice with PBS or with SalSol (sodium chloride 0.9%, 154 mmol/L NaCl, 308 mOsm/L) and diluted with the corresponding media to result 1% *V/V* final RBC concentration, then plated on a 96‐well, round‐bottom plate (Sarstedt, Nümbrecht, Germany). Serial dilution of the 3K peptide was prepared, starting from 250 µm, in the absence or presence of ADP or ATP (using the molar ratio of 1 to 4) and added to the RBC solutions in 4 parallels. Plates were incubated at 37°C, 5% CO_2_ for 2 h, centrifuged at 2000 rpm (5 min, 4°C) and then the supernatants were carefully transferred to a new plate, and the optical density (OD) was measured at 414 and 450 nm by a Multiskan GO microplate reader (Thermo Fisher Scientific, Vantaa, Finland). The percentage of hemolysis was determined compared to a well‐known hemolytic peptide, namely the bee venom Melittin (applied at 20 µm concentration). As control experiment, ADP and ATP solutions in PBS and in SalSol, without 3K peptide, were also prepared and added to the RBC (Figure S28 and Table S5). All data presented as the mean ± SD, N = 4). The half‐maximal hemolysis concentration (HC_50_) values were calculated from the dose‐response curves after fitting with non‐linear regression.

### 5.11 Cytotoxicity Activity Assays

To evaluate the cytotoxicity of the compounds, AlamarBlue viability assay was performed on MonoMac‐6 human monocytic cells (DSMZ no.: ACC 124, Braunschweig, Germany). MonoMac‐6 cell line shows stable phenotypic and functional characteristics of mature blood monocytes; therefore, it is a useful model for in vitro studies of monocyte biology and toxicity toward blood cells. Cells were maintained as an adherent culture in RPMI‐1640 media (Lonza, Basel), supplemented with 10% FCS, L‐glutamine (2 mm) and penicillin/streptomycin (50 IU/mL and 50 µg/mL) at 37°C in a humidified atmosphere containing 5% CO_2_. On the day of the experiment, cells were washed twice with PBS (pH = *7.4*) and plated in 96‐well flat‐bottom tissue culture plates (Sarstedt) (20 000 cells). 500 µm of the 3K‐APs was dissolved and serially diluted (250, 125, 62.5, 31.25, 15.63, 7.81, and 3.91, and 1.95 µm) in PBS and added to the cells. The final concentration range was between 1.95 and 250 µm and the final volume was 200 µL. After 2 h of incubation, cells were washed twice and then 22 µL AlamarBlue (resazurin sodium salt in PBS, pH *7.4*, c = 0.15 mg/mL) solution was added to each well. Following a 2 h of incubation, the fluorescence was measured at λ_Ex_ = 530/30 and λ_Em_ = 610/10 nm using a Synergy H4 multi‐mode microplate reader (BioTek). The percent of viability was calculated compared to untreated control wells. All measurements were performed in quadruplets and the mean values together with ± SD were represented. IC_50_ values were calculated from the dose‐response curves, after fitting with sigmoidal function using the GraphPad Prism software package.

### 5.12 Antibacterial Activity Assays

To investigate the antimicrobial activity of 3K‐ADP and 3K‐ATP systems, we utilized the previously described assay [21]. Briefly, the BL21 DE3 strain of *Escherichia coli (E. coli)*, following chloramphenicol selection (30 µm), was inoculated into fresh LB medium. This culture was grown until the optical density at 600 nm reached 0.5−0.6, indicating the exponential growth phase. Cells were harvested by centrifugation (3220 *g*, 4°C, 20 min) and washed twice with double distilled (dd) water to remove the growth medium. The resultant washed pellets were resuspended in 1/10 volume of dd water compared to the original culture. 10 µL aliquots of this bacterial suspension were mixed with a premixed solution of 3K and pH‐neutralized nucleotide (either ATP or ADP) in a total volume of 100 µL. 3K‐ATP and 3K‐ADP treatments were performed using fixed nucleotide to 3K molar ratios (4:1). Each concentration point of 3K‐ATP and 3K‐ADP had its control treatment containing only the nucleotide at the corresponding concentration in the absence of 3K. Treatments were carried out for 120 min, in sterile, low‐bind, U‐bottom 96‐well microplates (Greiner Bio‐One, Hungary) fitted with an oxygen‐permeable lid at 37°C, maintained under continuous shaking in a BioTek Synergy Mx plate reader. Post‐treatment, aliquots were taken from the wells and plated on LB agar plates. Colony‐forming units (CFU/mL) were determined after 16 h of incubation at 37°C. All treatments were performed in three biological replicates, with each biological replicate comprising three technical replicates for the CFU count. Viability is defined as the % ratio of colony forming units (CFU/mL) of 3K‐nucleotide‐treated and control cultures.

The minimum inhibitory concentrations (MICs) were determined using the standard broth microdilution method [50]. Eleven‐step, two‐fold serial dilutions of the tested compound were prepared in 96‐well microtiter plates using Luria‐Bertani (LB) medium (Millipore). Each well was inoculated with 5 × 10^5^ bacterial cells/mL in a final volume of 100 µL. The plates were incubated at 37 °C for 18 h with continuous shaking at 300 rpm. Each condition was tested in two technical replicates, and the entire experiment was repeated independently twice. Bacterial growth was assessed by measuring optical density at 600 nm (OD_600_) using a Biotek Synergy microplate reader. The MIC was defined as the lowest concentration of the compound that completely inhibited visible bacterial growth.

### 5.13 NMR

All measurements were performed on a Bruker Avance Neo 400 MHz instrument (161.9 MHz for ^31^P) equipped with a 5 mm BBO probe‐head with z‐gradient. The total volume of the sample was 730 uL and contained 10% D_2_O. Standard Bruker pulse sequences: *zgpr, noesygppr1d* for ^1^H, and *zgig* for ^31^P{^1^H} were used for measurements. Phosphorus environments in ATP are sensitive to pH. Therefore, an ATP solution (1 mm in water) was prepared first and then neutralized with NaOH to reach a pH = *6.9* before measurement. Then, the ATP solution was added to the 3K lyophilizate to form the complex 3K‐ATP, which was then measured at a 3K:ATP ratio of 250:1000 µm.

### 5.14 Molecular Dynamics (MD) Simulations

MD simulations were carried out using version 2019.4 of the GROMACS software, with a CHARMM force field extended for β‐peptides. The molecular models of lamellin‐3K were prepared using PyMOL version 2.4, using the pmlbeta extension. A 3D structure for ATP was taken from rcsb.org. Four stacks of six 3K peptides (altogether 24 peptides) were simulated with 100 ATP molecules in water. The box size was 7.8 nm x 7.8 nm x 7.8 nm. Physiological conditions and system neutralization were achieved by setting NaCl to 0.15 m in the system. Equilibration in the NVT ensemble was set to 300 K in a 100 ps simulation run using a velocity‐rescale thermostat. Equilibration in the NPT ensemble followed using a isotropic Berendsen barostat set at 1 bar. For the 1000 ns production run, the velocity‐rescaling thermostat and the Parrinello‐Rahman barostat were used. Long‐range electrostatics were treated by the particle mesh Ewald algorithm, while the short‐range Coulomb cut‐off was set at 1.2 nm as it is recommended by the CHARMM force field. Analysis of the simulation was performed using python scripts with the MDAnalysis python package.

### 5.15 Phosphate Release Assay

The kinetics of inorganic phosphate (P_i_) liberation from ATP was measured using Malachite Green Phosphate Assay Kit (MAK307, Sigma–Aldrich) at 25°C in a Tecan Infinite Nano+ plate reader instrument. ATP concentration was 250 µm in all ATP‐containing samples. Means and standard errors are shown for *n* = 3 (left panel) or *n* = 2 (right panel). OD600 values, corrected for those at *t* = 0 for each sample, were converted to P_i_ concentration values based on standard curves recorded using the P_i_ standard provided with the kit. Linear regression analysis of the data revealed no statistically significant positive slopes (i.e., no measurable P_i_ production, *p* > 0.05 in all cases) for any of the obtained datasets.

## Conflicts of Interest

The authors declare no conflicts of interest.

## Supporting information




**Supporting File**: advs74718‐sup‐0001‐SuppMat.docx.

## Data Availability

The data that support the findings of this study are available in the supplementary material of this article.
