# Supplementary material for: An ATP‐Mediated Antibiotic β‐Peptide Nanofiber That Kills Multidrug‐Resistant Bacteria via a Multistage Mechanism
Source: Adv Sci (Weinh). 2026 Mar 19;13(28):e22269. doi: 10.1002/advs.202522269 (PMC13185843; doi:10.1002/advs.202522269)
Supplement: Supplementary file 1 — Supporting File: advs74718‐sup‐0001‐SuppMat.docx. [file ADVS-13-e22269-s001.docx]

**Supplementary Information**

An ATP-Mediated Antibiotic β-Peptide Nanofiber that Kills Multidrug-Resistant Bacteria via a Multistage Mechanism

Sohini Chakraborty,^a§^ Kamal el Battioui,^a,g§^ Dániel Molnár,^b,h^ Bálint Jezsó,^a^ Lejla Daruka,^c^ Tasvilla Sonallya,^a,g^ Tünde Juhász,^a^ Rita Hirmondó,^b^ Imola Cs. Szigyártó,^a^ Loránd Románszki,^a^ Natália Tőkési,^a^ Daniel Pinkas,^d^ Eszter Házy,^e^ Olivér Pavela,^a,g^ Andrea Bodor,^f^ Zoltán Varga,^a^ István Mándity,^a,i^ Mihály Kovács,^e^ Csaba Pál,^c^ Kata Horváti,^j^ Judit Tóth,^b^ Tamás Beke-Somfai^a*^

^a^ Institute of Materials and Environmental Chemistry, HUN-REN Research Centre for Natural Sciences, Budapest H-1117, Hungary

^b^ Institute of Molecular Life Sciences, HUN-REN Research Centre for Natural Sciences, Budapest H-1117, Hungary

^c^ Synthetic and Systems Biology Unit, Institute of Biochemistry, HUN-REN Biological Research Centre Szeged, Szeged, Hungary

^d^ Cryo-Electron Microscopy and Tomography Core Facility, CEITEC, Masarykova University, Brno, Czech Republic

^e^ HUN-REN-ELTE Motor Pharmacology Research Group, Department of Biochemistry, Eötvös Loránd University, Budapest H-1117, Hungary

^f^ ELTE Eötvös Loránd University, Institute of Chemistry, Analytical and BioNMR Laboratory, Budapest H-1117, Hungary

^g^ Hevesy György Ph.D. School of Chemistry, Eötvös Loránd University, Budapest H-1117, Hungary

^h^ Doctoral School of Biology and Institute of Biology, Eötvös Loránd University, Budapest H-1117, Hungary

^i^ Department of Organic Chemistry, Faculty of Pharmacy, Semmelweis University, Budapest H-1092, Hungary

^j^ MTA–HUN-REN TTK Lendület “Momentum” Peptide-Based Vaccines Research Group, Institute of Materials and Environmental Chemistry, HUN-REN Research Centre for Natural Sciences, Budapest H-1117, Hungary

^§^ These authors contributed equally to this work

* Corresponding author: Tamás Beke-Somfai (e-mail address: beke-somfai.tamas@ttk.hu)

**This PDF file includes:**

Supplementary Figures 1 to 32

Supplementary Tables 1 to 6

**Table of Contents:**

1. **CD spectral characterization of 3K with oligophosphates.**
2. **ATR-IR spectra of 3K oligophosphates report on co-assembly formation.**
3. **ANS binding capacity is higher for 3K-ADP and 3K-ATP co-assemblies.**
4. **^1^H and ^31^P{1H} NMR analysis on 3K-ATP co-assembly.**
5. **Electron micrographs of 3K-AP show fibrils with striped patterns.**
6. **Calculated spatial dimensions of 3K-AP co-assemblies.**
7. **Quantification of the inter-array distances between peptide pairs via MD simulations.**
8. **Antibacterial activity of 3K-ADP and 3K-ATP on *E. coli*.**
9. **Minimum inhibitory concentration of 3K-ATP on *E. coli* BL21 DE3.**
10. **Minimum inhibitory concentration of 3K-ATP against antibiotic-adapted lines and multidrug-resistant *E. coli* strains.**
11. **TEM images of *E. coli.* cells treated with 3K-ATP and 3K-ADP.**
12. **Confocal microscopy images of *E. coli* treated with 3K-ATP with fluorescently labelled ATP.**
13. **Tapping mode AFM height and amplitude images of 3K-ATP on *E. coli*.**
14. **AFM height statistics of the individual *E. coli* cells before and after 3K-ATP treatment.**
15. **Cryo-EM of 3K-AP-treated *E. coli* cells.**
16. **Overview of the multistage antimicrobial mechanism of 3K-AP co-assemblies tracked via EM techniques.**
17. **Effect of 3K-ATP treatment on red blood-derived extracellular vesicles (REVs).**
18. **Hemolytic activity of 3K-ATP and 3K-ADP in NaCl and PBS media.**
19. **Cytotoxic effect of 3K-ATP and 3K-ADP in PBS medium.**
20. **Kinetics of inorganic phosphate (P_i_) release from ATP in the 3K-ATP co-assembly.**
21. **Comparison of the effects of ATP and phosphate ions from PBS upon addition to 3K.**
22. **High-resolution mass spectrum of purified 3K.**

**CD spectral characterization of 3K with oligophosphates**

To assess the interaction of 3K with the selected oligophosphates, conformational change of 3K was followed in a concentration dependent manner by CD spectroscopy. In phosphate-free conditions, 3K exhibits a random coil structure with a minimum at 213 nm and a maximum at 190 nm while in the presence of phosphate ions, a sharp positive signal is obtained at 206 nm suggesting the predominance of a sheet-like structure.^1^ When titrating 125 µM of 3K with 0-500 µM of the adenosine phosphates (AP), the CD spectra obtained show a similar pattern to those of the corresponding APs alone in line with their increasing concentration. In aqueous solutions these are characterized by a minimum in the region of 260 nm.^2^ The intensity of the peak in this region increased with AP concentration. The characteristic maxima at 192 nm and minima at 213 nm indicate changes in intensity and shifts in wavelengths, suggesting the formation of a more ordered structure. In case of 3K-AMP, the minimum at 213 nm was red shifted towards 217 nm with minimal changes in the intensity of the peak (Supplementary Figure 1a and 3a). For 3K-ADP, the minimum at 213 nm was retained along with the appearance of a shoulder peak at 215 nm (Supplementary Figure 1b and 3b). For 3K-ATP, the intensity of this region almost doubled when compared to the other AP’s along with the slight shoulder peak at around 216 nm (Supplementary Figure 1c and 3c). The formation of co-assemblies could be responsible for the loss of intensity as well as superimposition of the CD curves.^3^ Among all the investigated systems, the conformational changes were not obvious from the representative spectra even after sonication (Supplementary Figure 2). Therefore, we carried out the subtraction of the CD spectra of the phosphate agents alone from the spectra of the co-assembled peptide+phosphate agent to monitor conformational changes as a function of increasing concentration in these systems (Supplementary Figure 3). The spectra reveal that the random coil conformation of 3K in water is not preserved at higher concentrations of oligophosphates. There is an appearance of two negative bands of almost equal intensity at higher concentrations of AMP as observed in helical peptides (Supplementary Figure 3a). The same pattern is observed for 3K-ADP and 3K-ATP with a clear dissimilarity in the intensities of the two minima with the second minima resembling a shoulder band with intensities closer to 220 nm (Supplementary Figures 3b,c). This CD signature has been reported earlier for peptides adopting a partly helical nature.^4^ As these conformational changes are more prominent at higher concentrations, we maintain a 3K-phosphate ratio of 1:4 for all other characterizations.


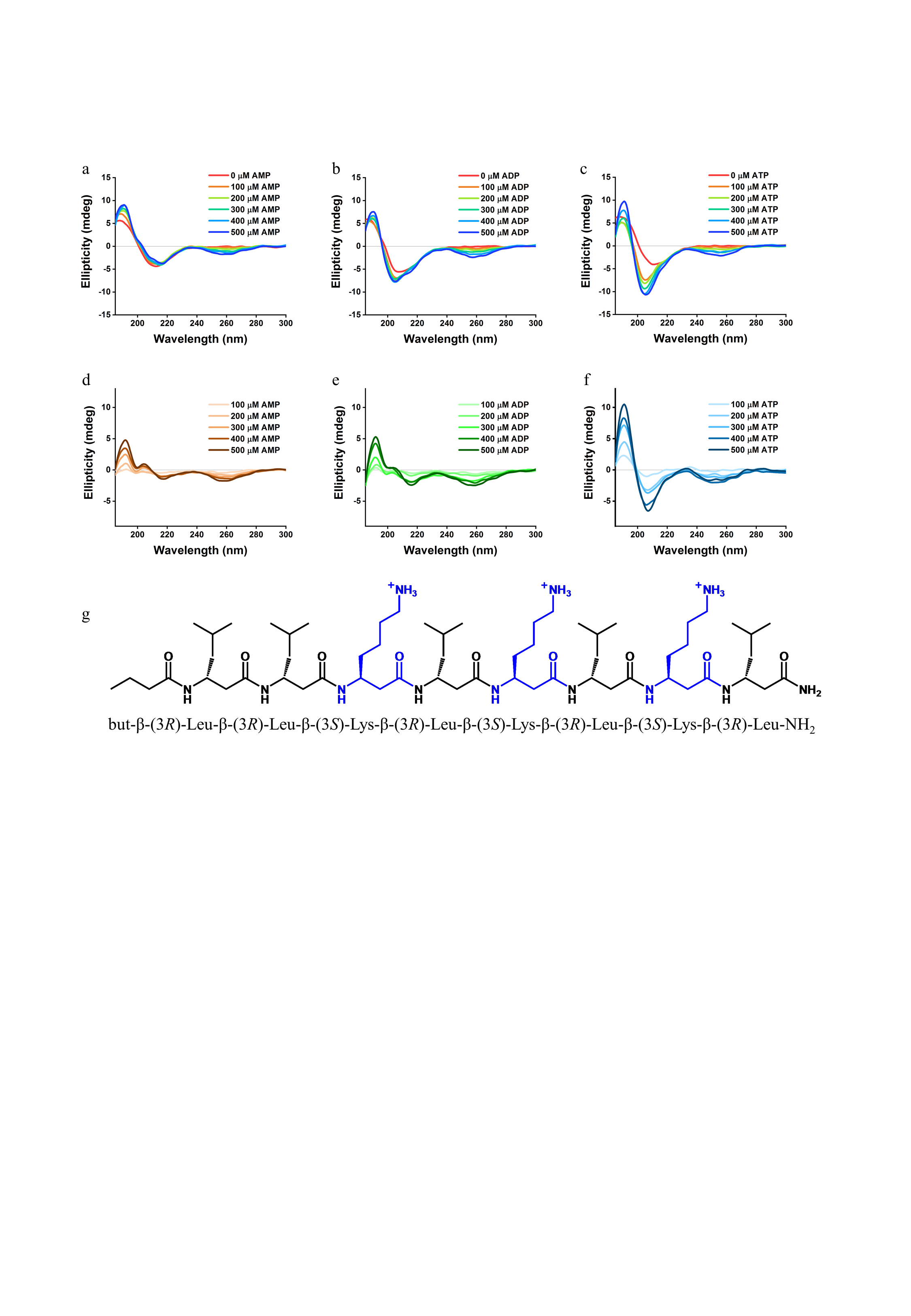


3K+APs

APs control

**Supplementary Figure 1. Circular dichroism spectra of 3K upon titration with APs.** CD spectra of 3K (125 μM) titrated with a) AMP, b) ADP and c) ATP. CD spectra of APs in water: d) AMP, e) ADP and f) ATP. g) Sequence of 3K with the leucines marked in black and the lysines in blue.


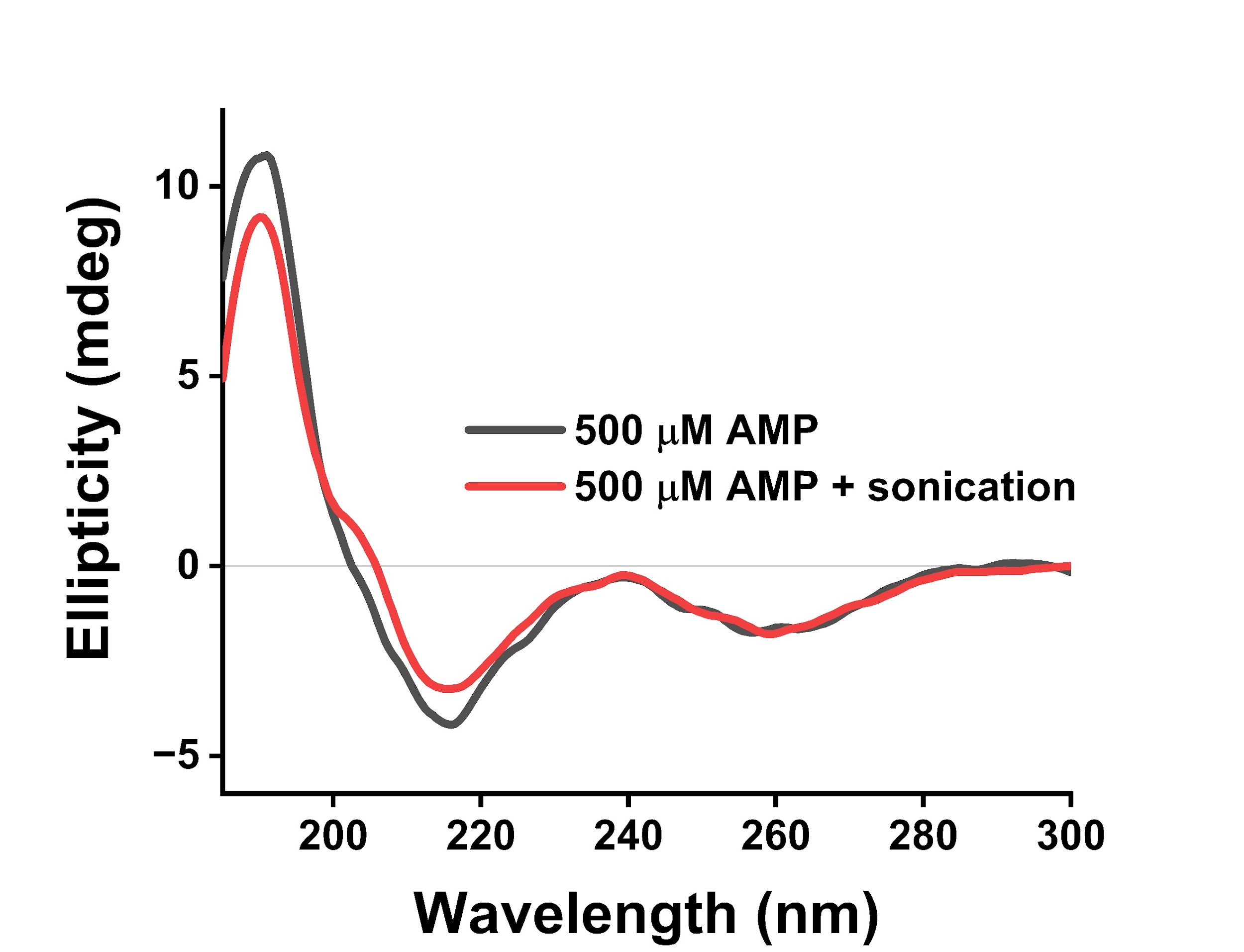


a


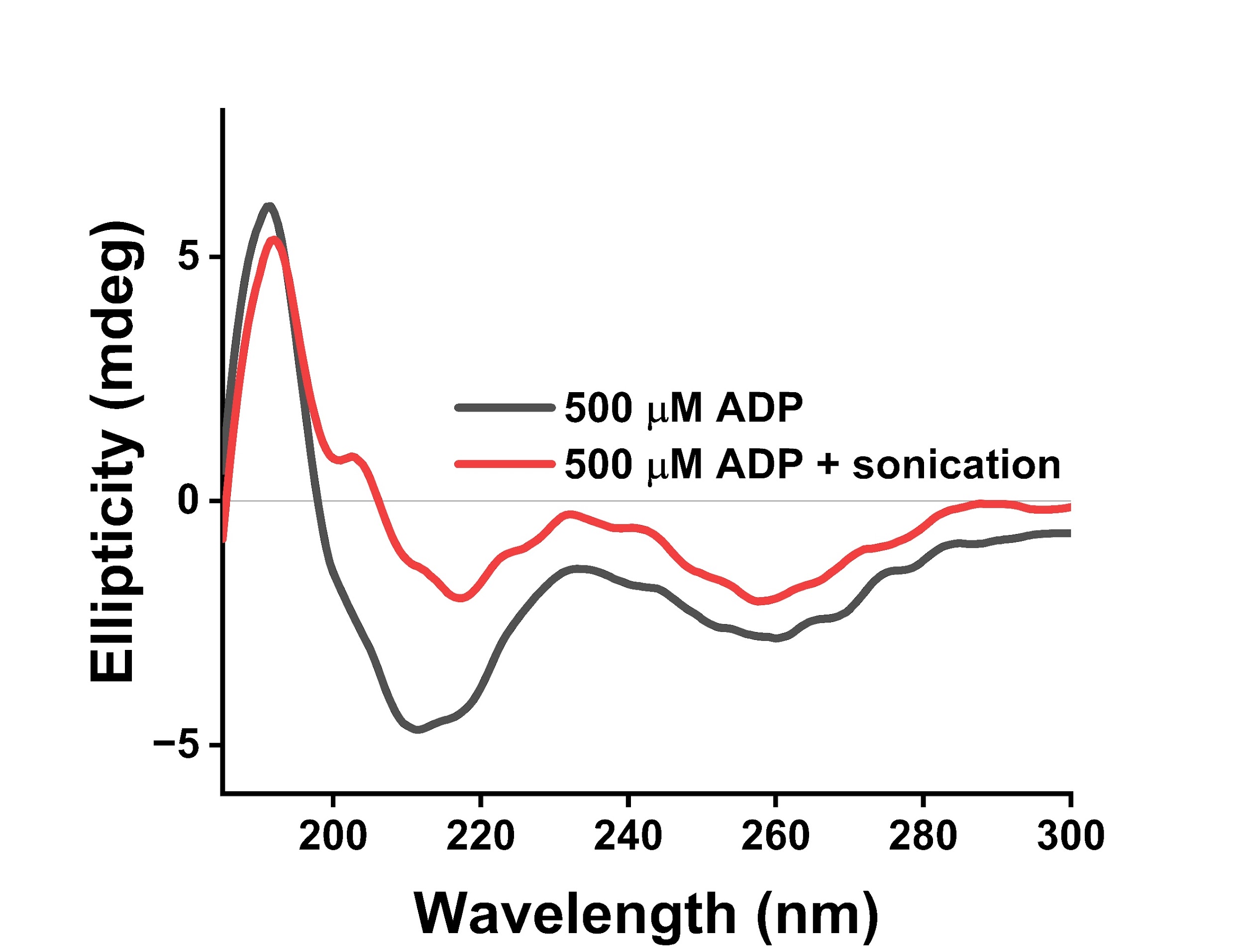


b


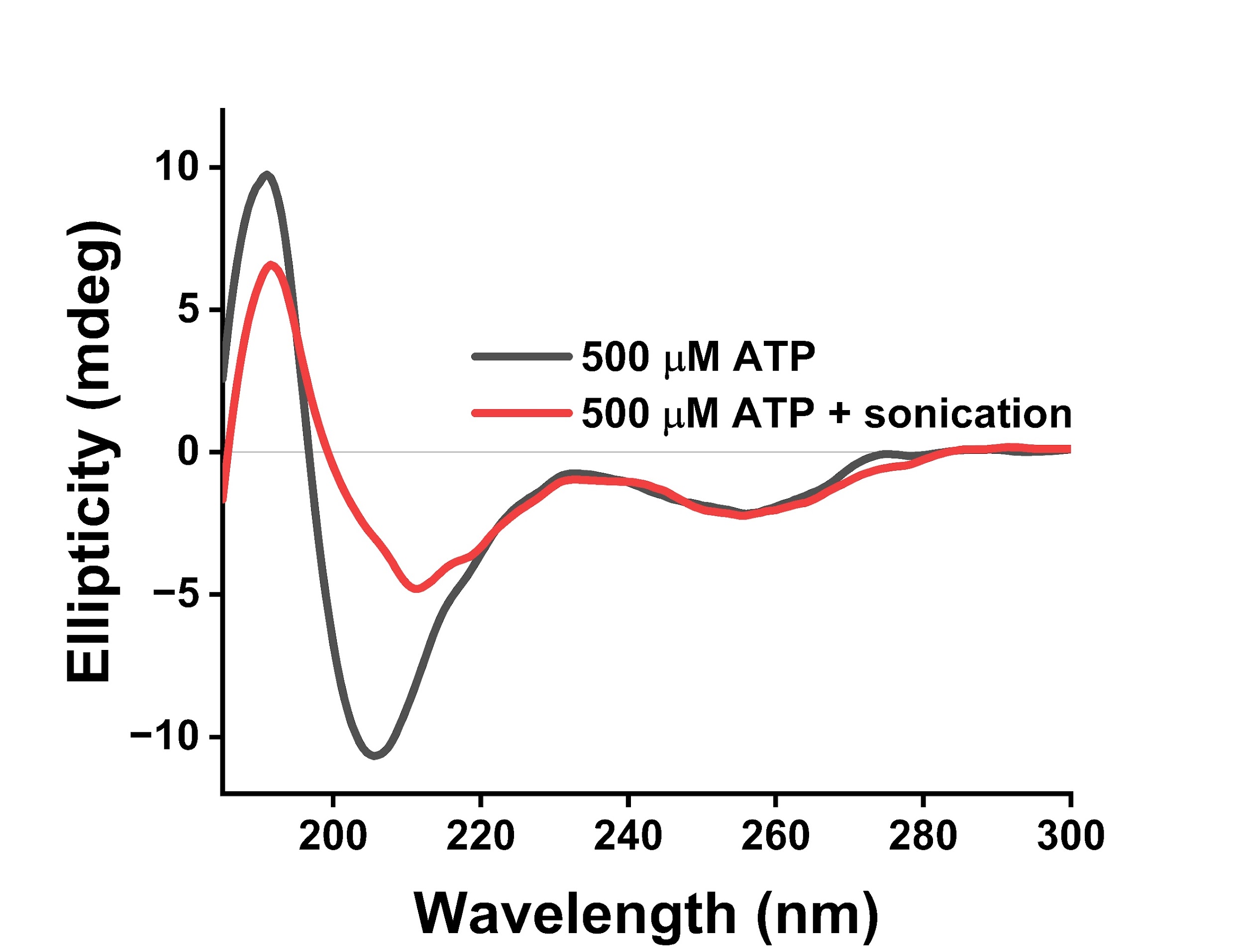


c

**Supplementary Figure 2. Effect of sonication on 3K-APs assemblies.** CD spectra of 3K (125 µM) + phosphate agents (500 µM) before and after 30 min of sonication for a) AMP, b) ADP and c) ATP.


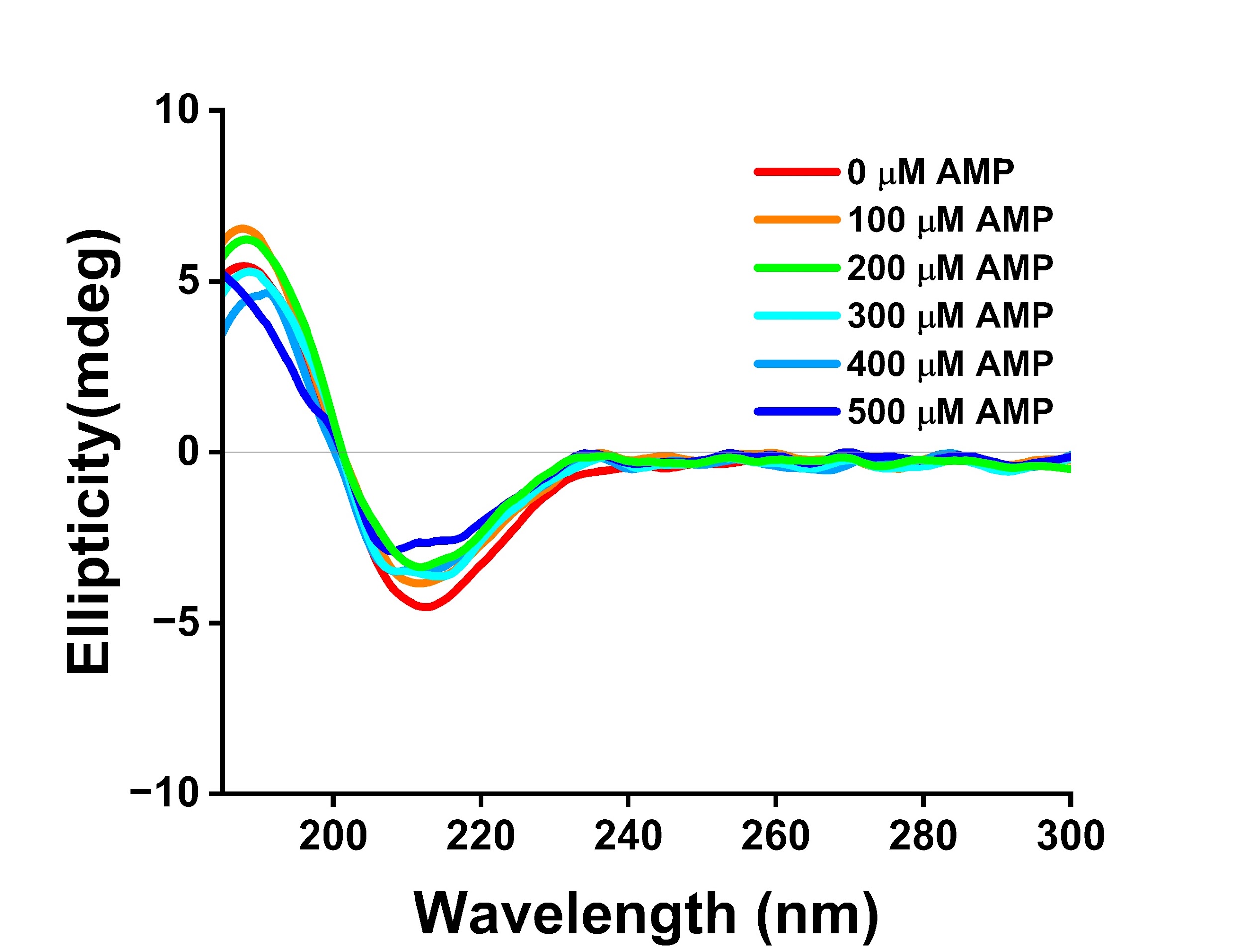


a


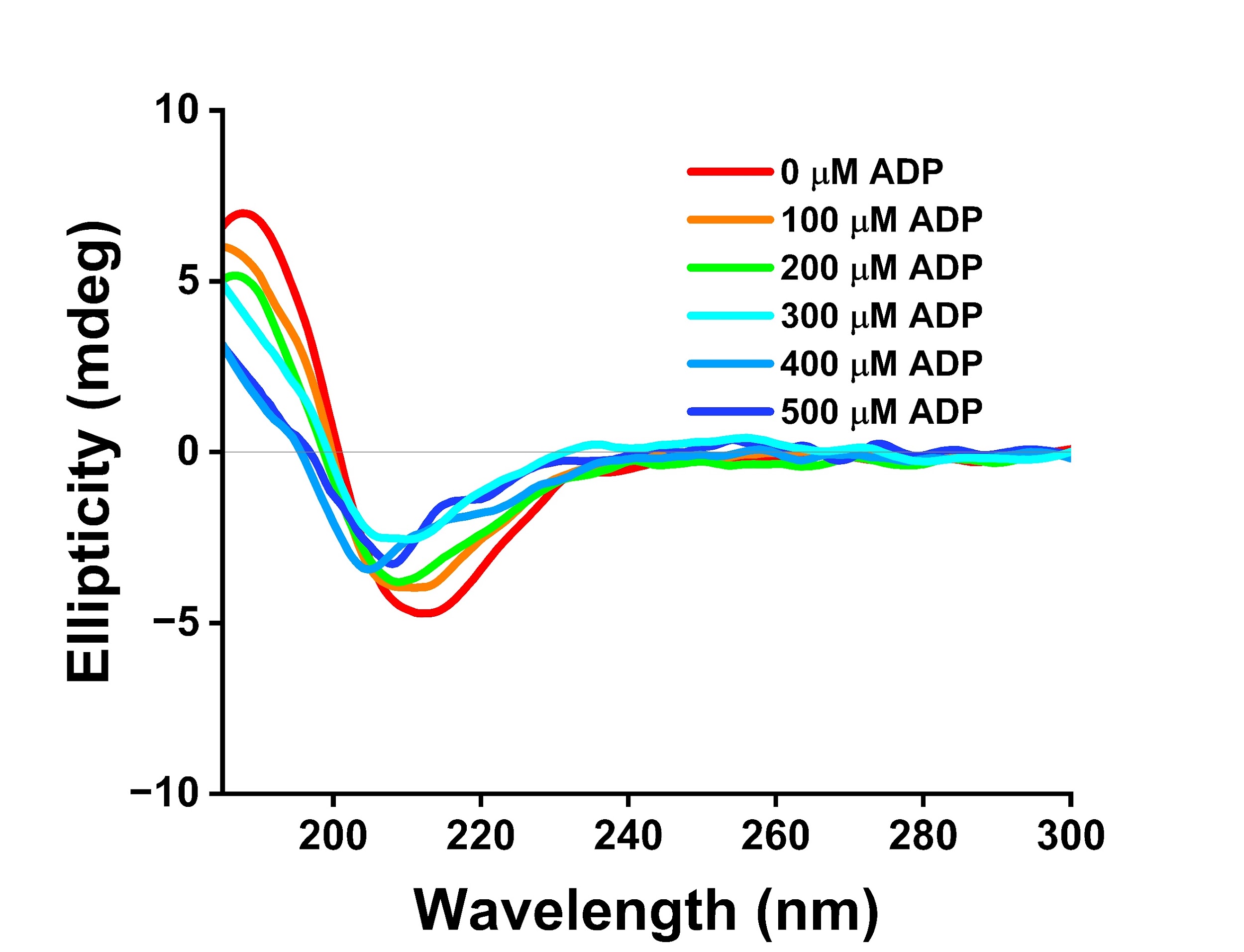


b


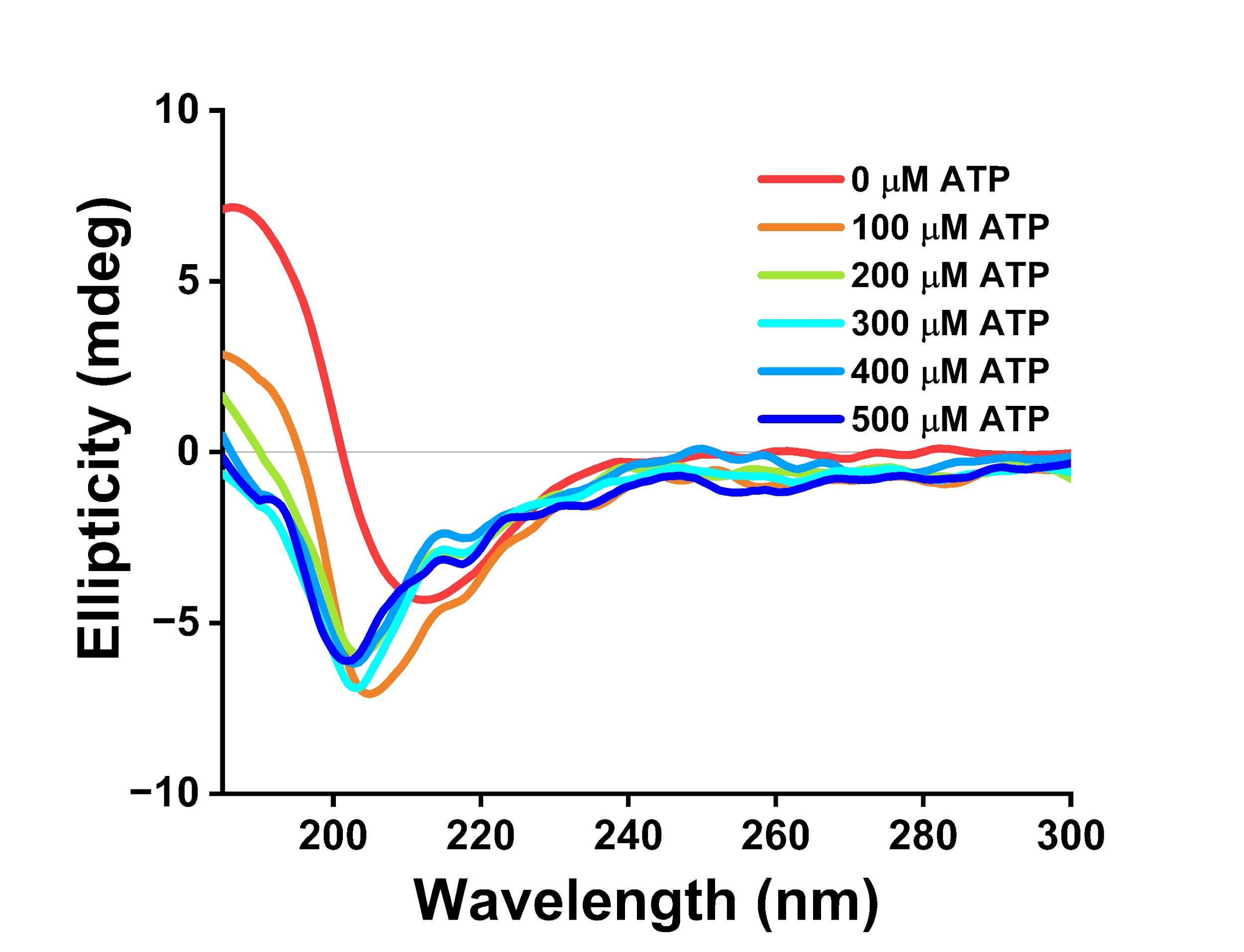


c

**Supplementary Figure 3. CD spectral subtraction to monitor conformational change upon APs titration.** CD spectra obtained by subtracting the CD spectra of phosphate agents from the CD spectra of 3K+phosphate agents for a) AMP, b) ADP, and c) ATP. During titration of 3K with increasing concentrations of AP, no significant changes in spectral signatures were observed, indicating that the secondary structure of the 3K assemblies remains largely unchanged across the tested ratios. Notably, comparison of titration experiments with the different APs (Supplementary Figure 1) reveals a peak at ~213 nm that is not directly related to the added APs.

This is particularly evident in the subtraction spectra (Supplementary Figure 3) indicating that assembly formation between 3K and ATP occurs spontaneously, accompanied by a slight but not substantial conformational change. For most 3K-AP systems, the secondary structure displays minimal variations; however, in the case of 3K-ATP, titration experiments suggest that the addition of 100 μM of ATP is sufficient to initiate a conformational rearrangement (Supplementary Figure 3c). Further increases in ATP concentration do not produce additional changes in the CD spectra, indicating that the system rapidly reaches saturation, corresponding to a 3K-ATP ratio of 1:4. Therefore, the structural organization appears to be governed primarily by molecular interaction rather than by mixing ratio.

**ATR-IR spectra of 3K oligophosphates report on co-assembly formation.**

IR spectroscopy was also used to validate the co-assembly formation and assess potential conformational changes. For 3K in water, the amide I and amide II peaks characteristic of amide bonds in peptides and proteins, appear at 1600–1700 cm^-1^ and 1500–1600 cm^-1^ respectively (Supplementary Figure 4). The strong amide I band component centered at 1650 cm^-1^ can be attributed to the dominant random coil conformation, consistent with previous correlated NMR investigations.^1,5^ A small shoulder band at 1685 cm^-1^ is also observed which can be attributed to the free amide CO groups, which are not involved in hydrogen bonding.^6^ For 3K-AMP, this peak reduces in intensity and it almost disappears for 3K-ADP (Supplementary Figures 4b,c). This is due to the availability of more binding sites for the higher order phosphates resulting in increased hydrogen bonding interactions leading to the formation of structured supramolecular co-assemblies. For 3K-ATP, the shoulder peak reappears at 1684 cm^-1^ which is a contribution from the scissoring vibrations of the pendant -NH_2_ of the adenine moiety and is also prevalent in free ATP (Supplementary Figure 4d).^7–9^ Additionally, peaks at ~1600 cm^-1^ and ~1570 cm^-1^ in the spectra of free AP’s can be attributed to the purine ring.^10,11^ These peaks are also observed in the spectra of 3K-AP systems, although slightly shifted and at higher intensities indicating effective co-assembly formation. In the phosphate region (1300–900 cm^-1^), APs display peaks at around 900, 1075 and 1240 cm^-1^ that can be assigned to the P-O stretching vibrations of the main chain, out of phase symmetrical stretching vibration and the overlapping of the asymmetric stretching vibrations between the α-PO_2_^-^ and β-PO_2_^-^ respectively (Supplementary Figures 4e–g).^7^ In addition to these peaks, well-resolved phosphate vibration bands are observed for the 3K complexed systems suggesting the presence of extended interactions between the peptide and the phosphates.


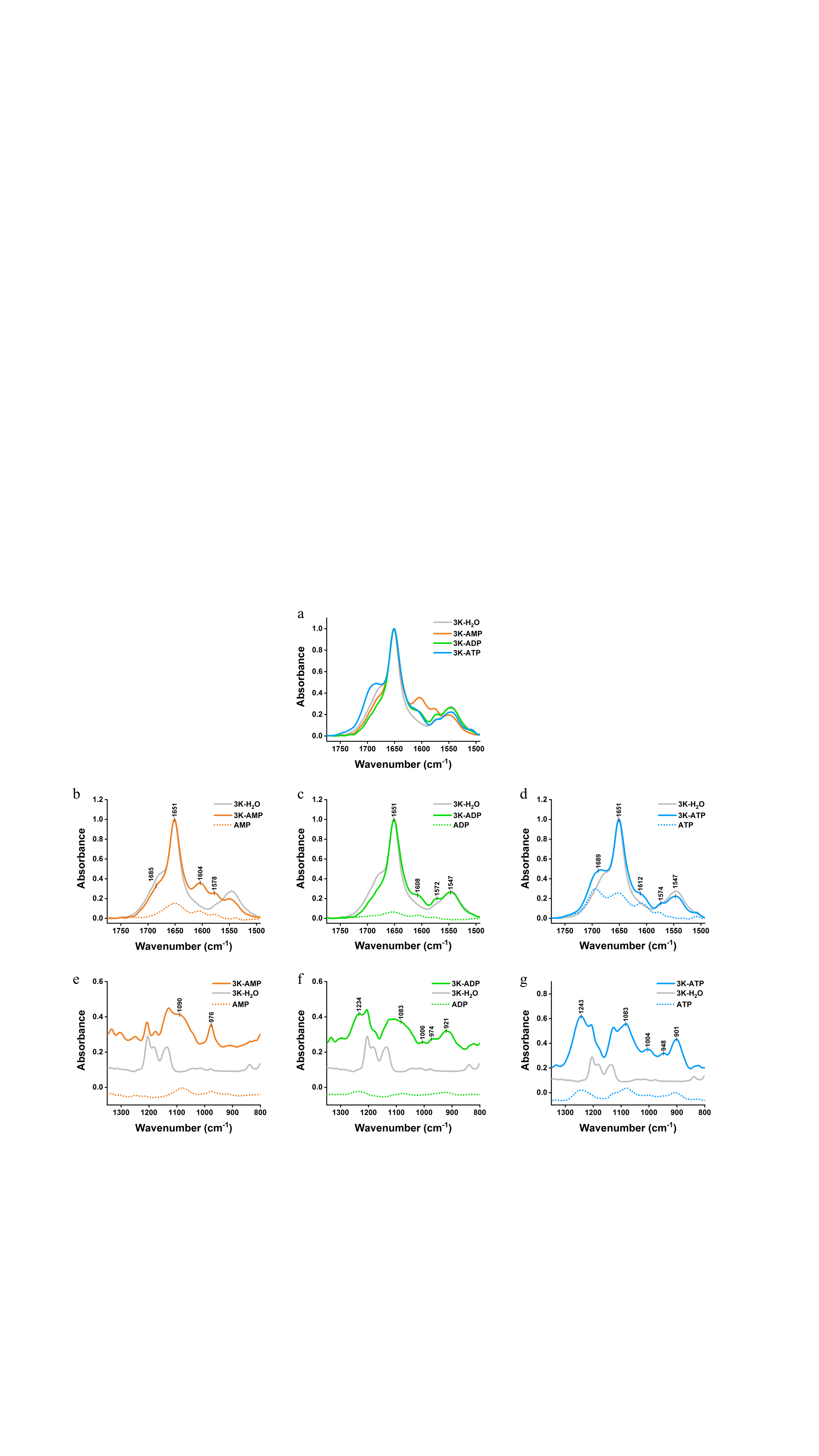


**Supplementary Figure 4.** **Characterization of 3K-APs coassemblies by ATR-IR spectroscopy.** a) IR spectra of the amide region of the 3K-APs coassemblies. Comparative IR spectra of the amide region of the 3K-AP, AP and 3K-H_2_O coassembly in the case of, b) 3K-AMP, c) 3K-ADP and d) 3K-ATP. Comparative IR spectra of the phosphate region of the 3K-AP, AP and 3K-H_2_O coassembly in the case of, e) 3K-AMP, f) 3K-ADP and g) 3K-ATP. The concentration ratio 3K to AP is 1:4 (125 μM 3K and 500 μM AP). 3K-H_2_O and AP in water have a concentration of 125 μM and 500 μM respectively.

**ANS binding capacity is higher for 3K-ADP and 3K-ATP co-assemblies**

Fluorescence spectroscopy was used to study the interactions between the 3K-AP systems and a water-soluble anionic dye, 1-anilinonaphthalene-8-sulfonate (ANS). The fluorescence intensity of 3K-ADP and 3K-ATP was three-fold higher than that of 3K-ATP and phosphate-free 3K. Note that ANS binding occurs predominantly through hydrophobic interactions with aromatic moieties and electrostatic ion pair formation with the sulphonate group, increasing fluorescence intensity.^12^ As no significant change in the secondary structure was detected from the CD curves, it can be inferred that the rapid co-assembly formation was mostly dependent on the electrostatic forces of interaction between the positively charged 3K and the negatively charged APs. Note that ANS fluorescence was observed to be slightly higher in the case of ATP while for the other phosphate agents, it belongs to the same range.


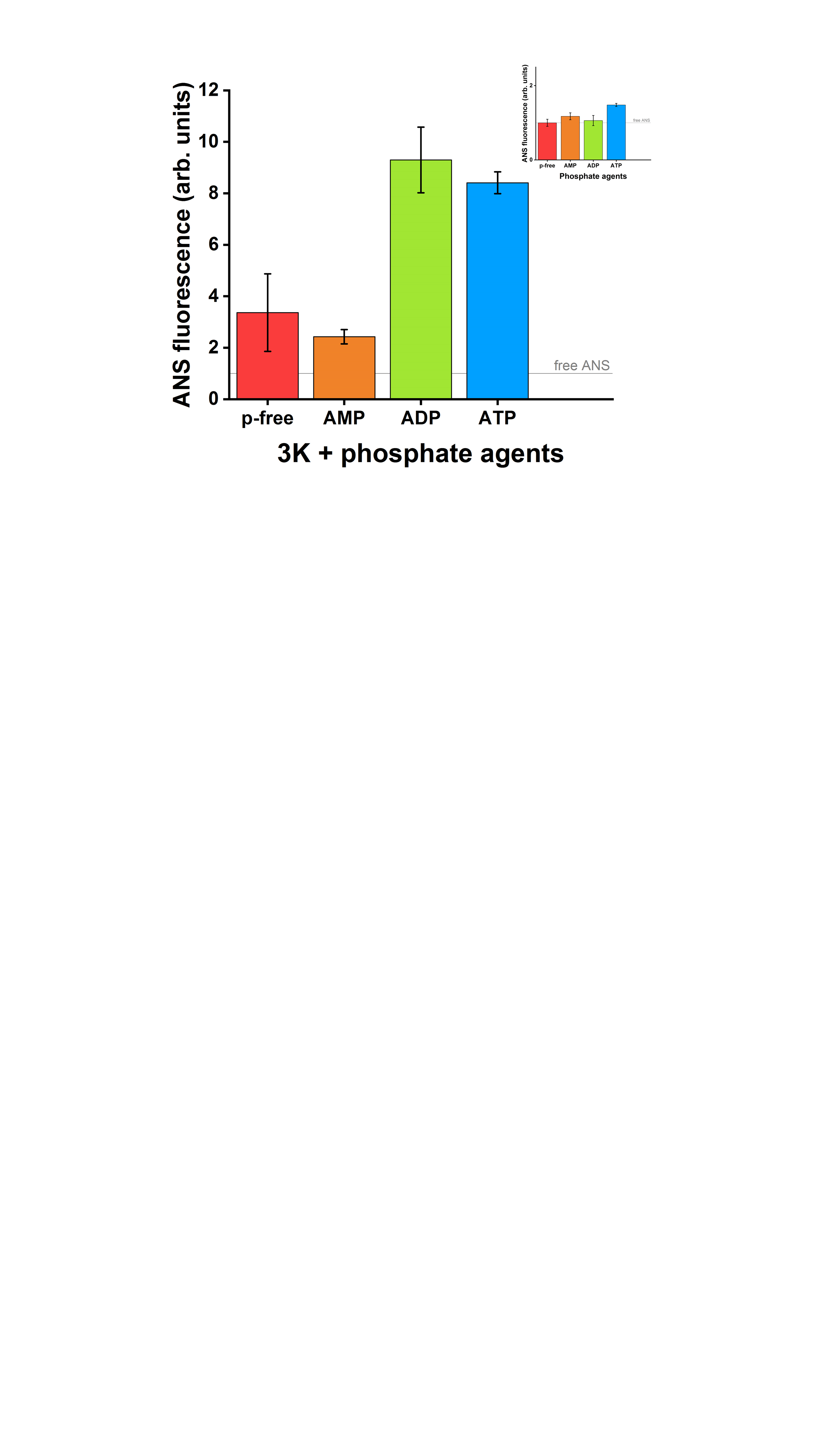


**Supplementary Figure 5. ANS binding capacity for 3K-APs co-assemblies**. ANS binding of 3K, 125 μM with 500 μM of different phosphate agents (AMP, ADP, ATP and non-phosphate source as a control) using the ANS probe at 5 μM in water. The emission intensities at the maxima were read, and values were normalized for that of unbound ANS. Gray line highlights the reference signal of free ANS. The inset shows the ANS binding of 500 μM of different phosphate agents (AMP, ADP, ATP and non-phosphate source as a control) using the ANS probe at 5 μM in water. Error bars represent the standard deviation of the mean for three independent experiment sets.

**^1^H and ^31^P{1H} NMR analysis on 3K-ATP co-assembly**

The atomic level interaction between ATP and 3K at 4:1 ratio was studied by ^1^H and ^31^P NMR methods. In this respect, variations occurring in all regions of ATP can be followed by monitoring the changes in chemical shift and integral values. Supplementary Figure 6 shows the segment of the ^1^H NMR spectra, which highlights the singlet peaks (8.42 and 8.15 ppm, respectively) of the adenine part and the doublet C*H* peak (6.03 ppm, ^3^J_HH_ = 6.1 Hz) of the ribose ring connected to the adenine. Further resonances of the ribose environment are distorted, as they fall in the vicinity of the residual solvent peak (4.7 ppm; not shown). The three environments of the triphosphate side-chain are well separated peaks in ^31^P NMR spectra: α (-11.4 ppm, doublet, ^2^J_PP_ = 20 Hz), β (-22.9 ppm, triplet, ^2^J_PP_ = 20 Hz), and γ (-9.41 ppm, broad due to H^+^ exchange).

Addition of lyophilized 3K causes a visible colloid formation, followed by a white precipitate formation on the bottom of the NMR tube. Integral values of the highlighted resonances in the ^1^H spectra show a 50% decrease, meaning about half of the ATP is precipitated from the solution. Accordingly, this integral decrease is detected from the ^31^P{^1^H} measurements (red spectra). Almost all observed chemical shift values are constant, meaning the ATP remained in solution is in free form. Only a small variation towards lower values is detected for the Pγ environment (-9.90 ppm) which indicates that the solution became more acidic. Indeed, pH measurement showed that addition of 3K causes a slight change from pH = 6.9 to pH = 6.7.


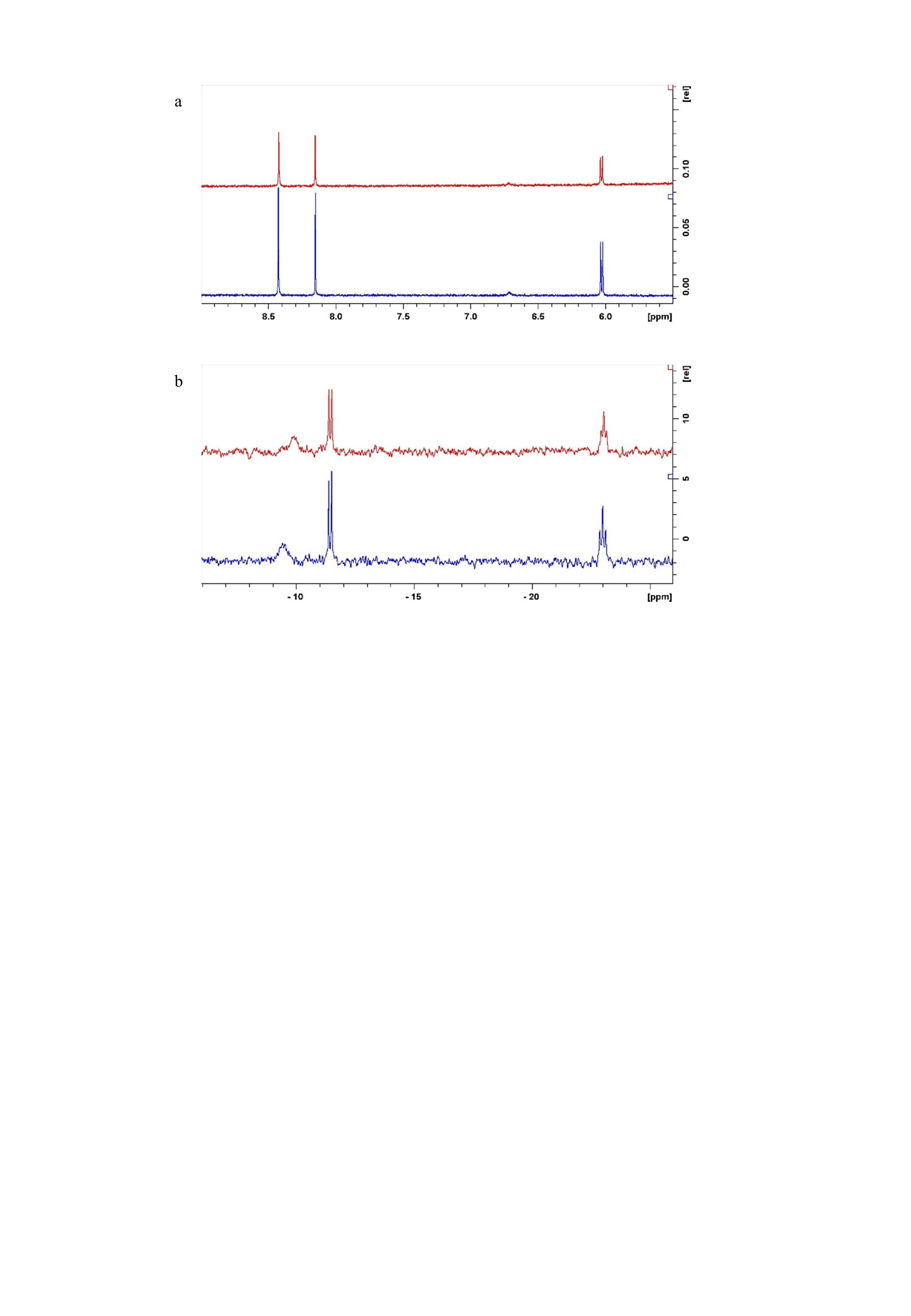


**Supplementary Figure 6.** **Molecular insights into the formation of 3K-ATP coassemblies via NMR spectroscopy.** ^1^H (*noesygppr1d*, NS = 32; left panel) and ^31^P{^1^H} (*zgig*, NS = 160; right panel) for the neat 1mM ATP pH = 6.9 sample (blue) and for the 1mM ATP:250 μM 3K pH = 6.7 sample (red).


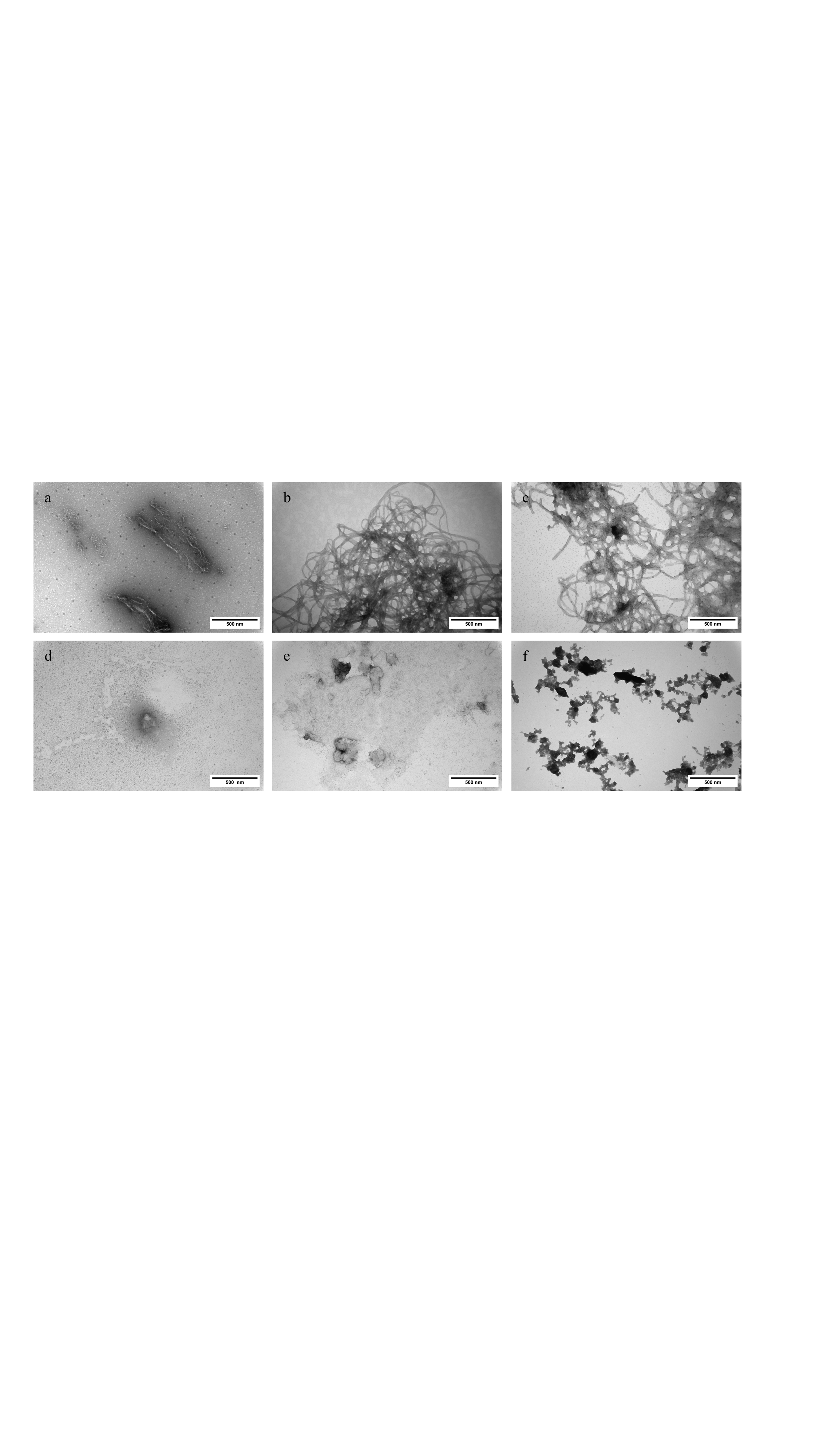


**Supplementary Figure 7. Morphological characterization of 3K-APs coassemblies by NS-TEM.** TEM images of a) 3K-AMP, b) 3K-ADP and c) 3K-ATP. The concentration ratio 3K to AP is 1:4 (125 μM 3K and 500 μM AP). TEM images of APs (500 µM) in water as control for d) AMP, e) ADP and f) ATP.


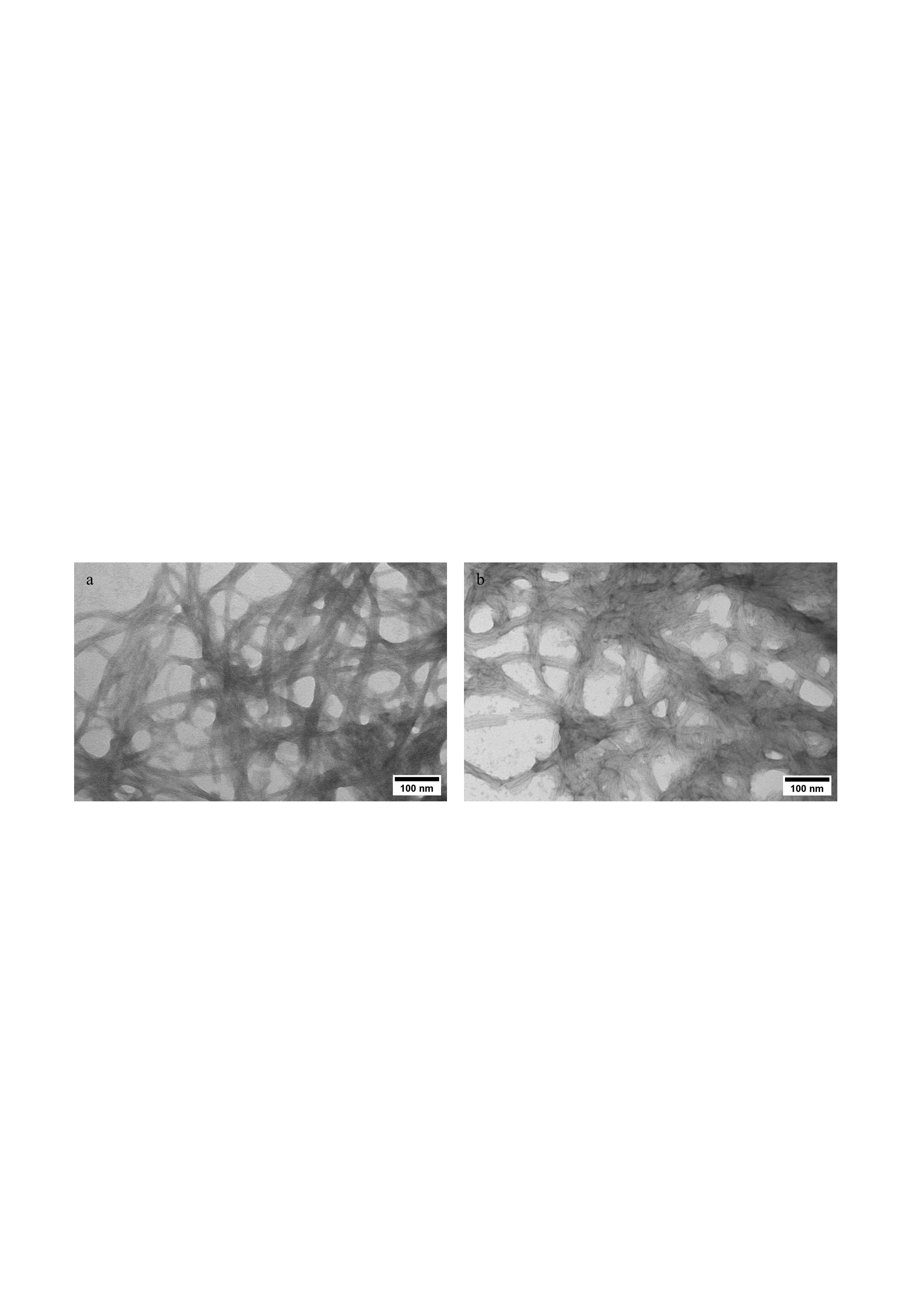


**Supplementary Figure 8. Striped morphologies observed in 3K-APs coassemblies by NS-TEM.** TEM images revealing characteristic striped patterns in a) 3K-ADP and b) 3K-ATP. The concentration ratio of 3K to AP was 1:4 (125 μM 3K and 500 μM AP).


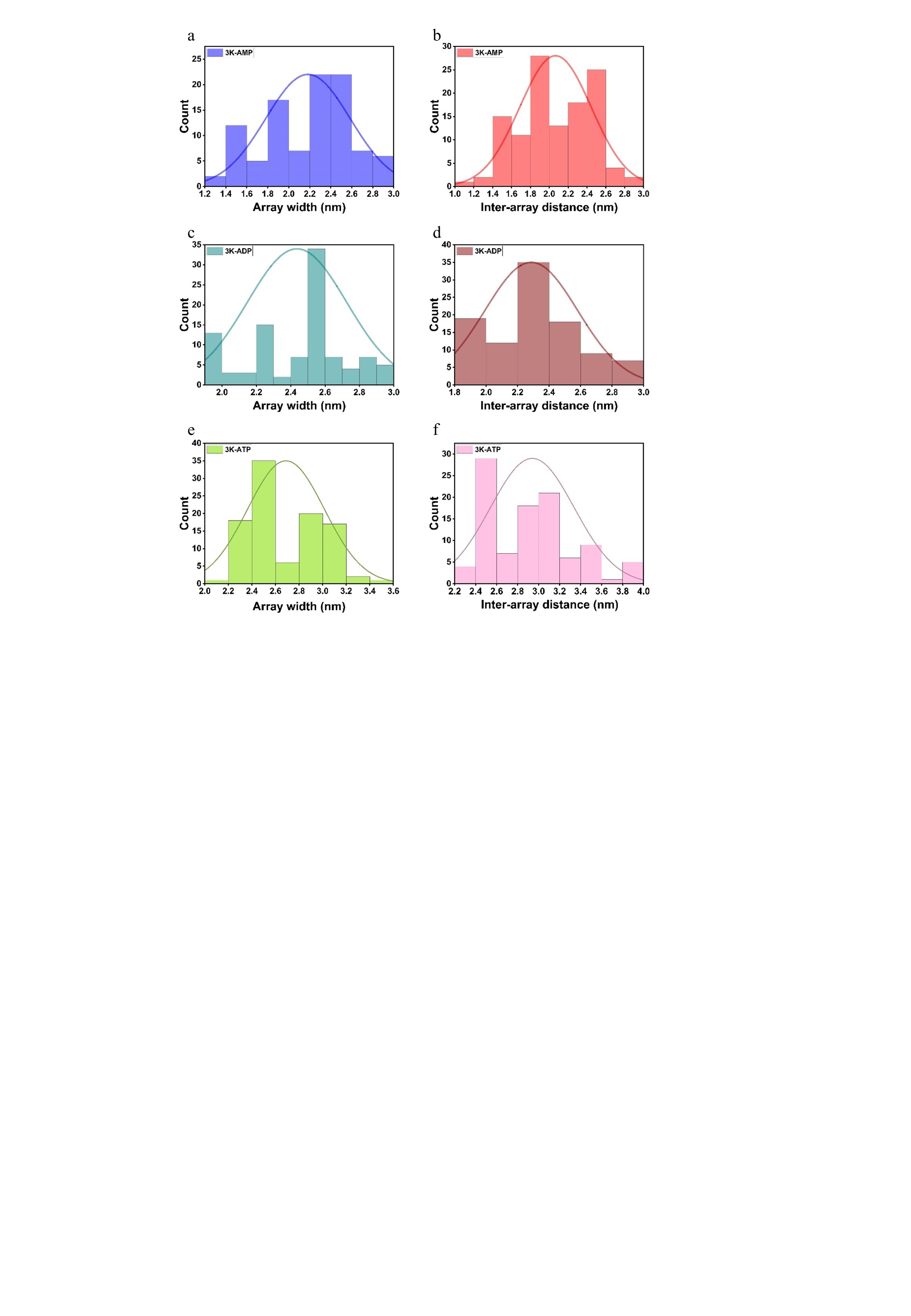


# **Supplementary Figure 9. Calculated spatial dimensions of 3K coassemblies.** Histograms showing size distribution for various 3K coassemblies. a-b) 3K-AMP, c-d) 3K-ADP, e-f) 3K-ATP. Values were determined from TEM images analyzed with ImageJ software. Distance parameters are summarized in Supplementary Table 1.

**Supplementary Table 1. Spatial dimensions of 3K coassemblies.** Values were calculated for 100 particles from parameters determined based on TEM images using the ImageJ software.

| **Coassembly** | **Parameter** | |
| --- | --- | --- |
|  | **Array width (nm)** | **Inter-array distance (nm)** |
| 3K-PBS | 1.60 ± 0.22 | 1.13 ± 0.13 |
| 3K-AMP | 2.18 ± 0.40 | 2.09 ± 0.39 |
| 3K-ADP | 2.44 ± 0.29 | 2.29 ± 0.29 |
| 3K-ATP | 2.69 ± 0.32 | 2.94 ± 0.40 |

.

**Extended details on model employed for MD simulations**

The main parameters and setup details of the molecular dynamics (MD) simulations involving 24 3K peptides and 100 ATP molecules are described in the main text. The simulation system was derived from a previously published system containing 24 3K peptides and 100 methyl phosphate molecules,^1^ with initial peptide assemblies (6-mer units) extracted from our earlier simulation. The present MD model and simulation was specifically designed to investigate the inter-array distances between assembled peptides in presence of ATPs and compare them with previous models using phosphates as counterions, and with experimental measurements obtained here from TEM micrographs analyzed using ImageJ (Supplementary Table 1). Given the larger steric volume of ATP molecules, an increase in the inter-array distance was anticipated and obtained when compared to the models containing phosphates. However, the system exhibited considerable structural fluctuations, making it challenging to identify peptide pairs that reliably represented the spacing of interest. Ultimately, a subset of peptide pairs from distinct 6-mer assemblies was selected by visual inspection of the trajectory, where clear ATP filled gaps were present between the peptides and lysine residues were oriented toward the ATP molecules. Based on this consideration a total of 4 peptide pairs were identified, where each peptide within the pairs were facing towards each other separated by only ATP molecules. For those peptides the relative distance between them was monitored as described in the caption of Supplementary Table 2.

The input files and coordinate files for the simulations are available at: <https://zenodo.org/records/15738308?token=eyJhbGciOiJIUzUxMiJ9.eyJpZCI6ImM5NDU2NGNjLTM5YTQtNDc1Mi04MjlmLWExNmU1ZGM4MDdjNCIsImRhdGEiOnt9LCJyYW5kb20iOiIyZTM5ZjljYzMwNTJlYTA2ODczNjM4YzU4MDZjOWIwMyJ9.EIpcdTvMi6svYZfDE7k4VD8JW75KFTuOlgoBjQnwRljo5VRTBsrSvOkuvgjuAJLP-t3SedEYRk5wCJV6ffM2FQ>

Note: at this stage, these are reviewer-only materials, where anonymous access is provided. Upon acceptance, the link will be made publicly available.


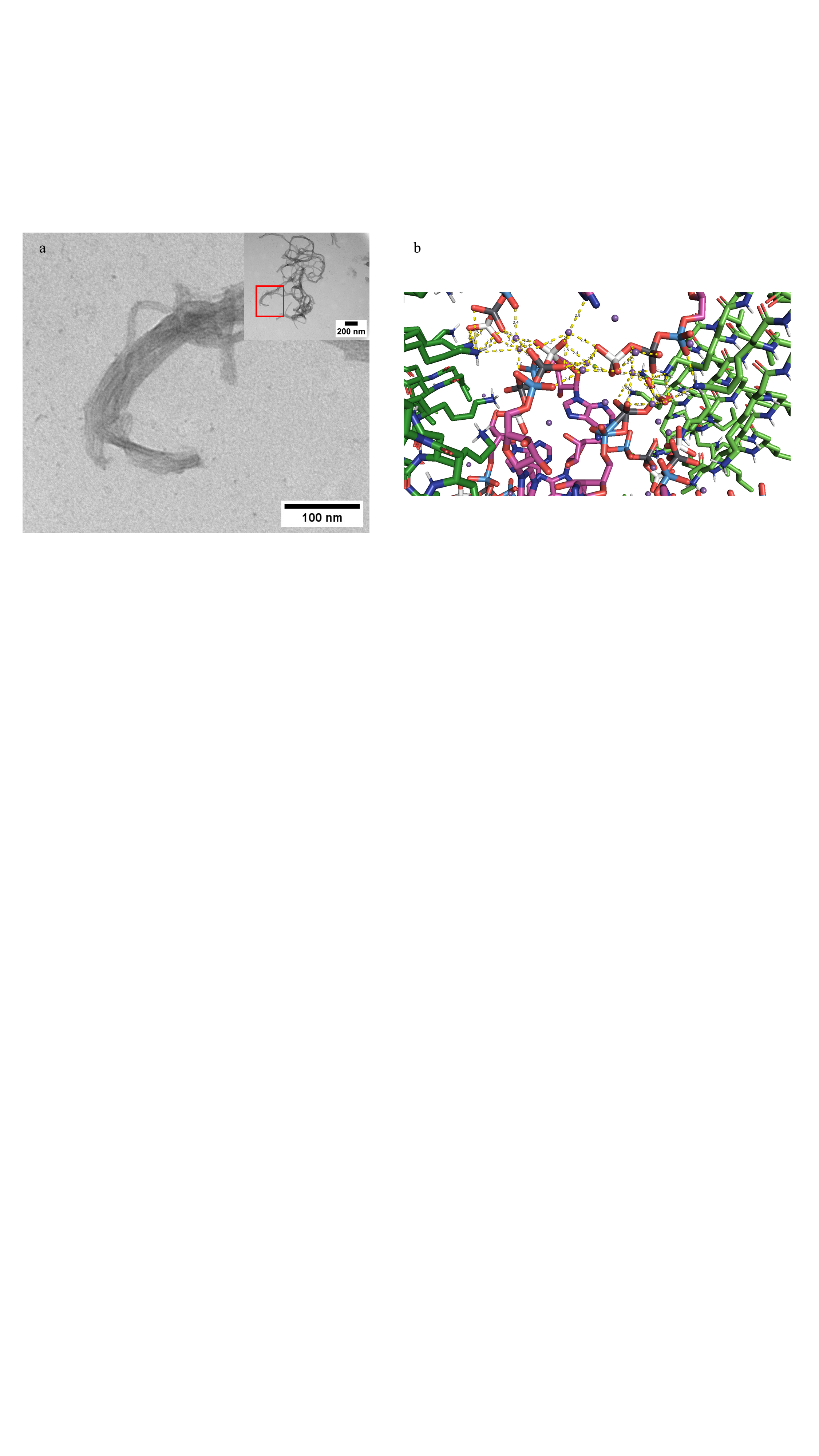


**Supplementary Figure 10.** **Structural and simulation insights of 3K-ATP**. a) TEM image of 3K-ATP.The concentration ratio of 3K to AP was 1:4 (125 μM 3K and 500 μM AP). b) MD simulation snapshot showing the hydrophobic and hydrophilic regions in 3K-ATP, consistent with the array and inter-array patterns observed as white and black stripes, respectively, in the TEM image (a). Multiple ATP molecules (shown in purple) are located between the peptide arrays (green), which are interconnected via several salt bridges (indicated by yellow dotted lines).

**Supplementary Table 2.** **Quantification of the inter-array distances between peptide pairs via MD simulations.** The inter-array distance was calculated for selected peptide pairs by measuring the distances between the β-carbon atoms of the lysine residues. Eight peptides were analyzed out of 24 peptides, as transient fluctuations in the system allowed meaningful pairwise matching for these combinations within a limited time interval. For each peptide pair, the distances between the β-carbon atoms of the lysine residues were measured at positions 3, 5, and 7 (i.e., 3Lys–3Lys, 5Lys–5Lys and 7Lys–7Lys), corresponding to the lysines in the 3K sequence (Supplementary Figure 1). The calculation was performed over the final 50 ns of the simulation, with distance values averaged at 1 ns interval. The values over this 50 ns period are presented in the following table as mean ± SD.

| **Peptide pair** | **Average distance (nm)** |
| --- | --- |
| Peptide pair 1 | 2.54 ± 0.53 |
| Peptide pair 2 | 2.55 ± 0.33 |
| Peptide pair 3 | 2.47 ± 0.47 |
| Peptide pair 4 | 2.43 ± 0.48 |


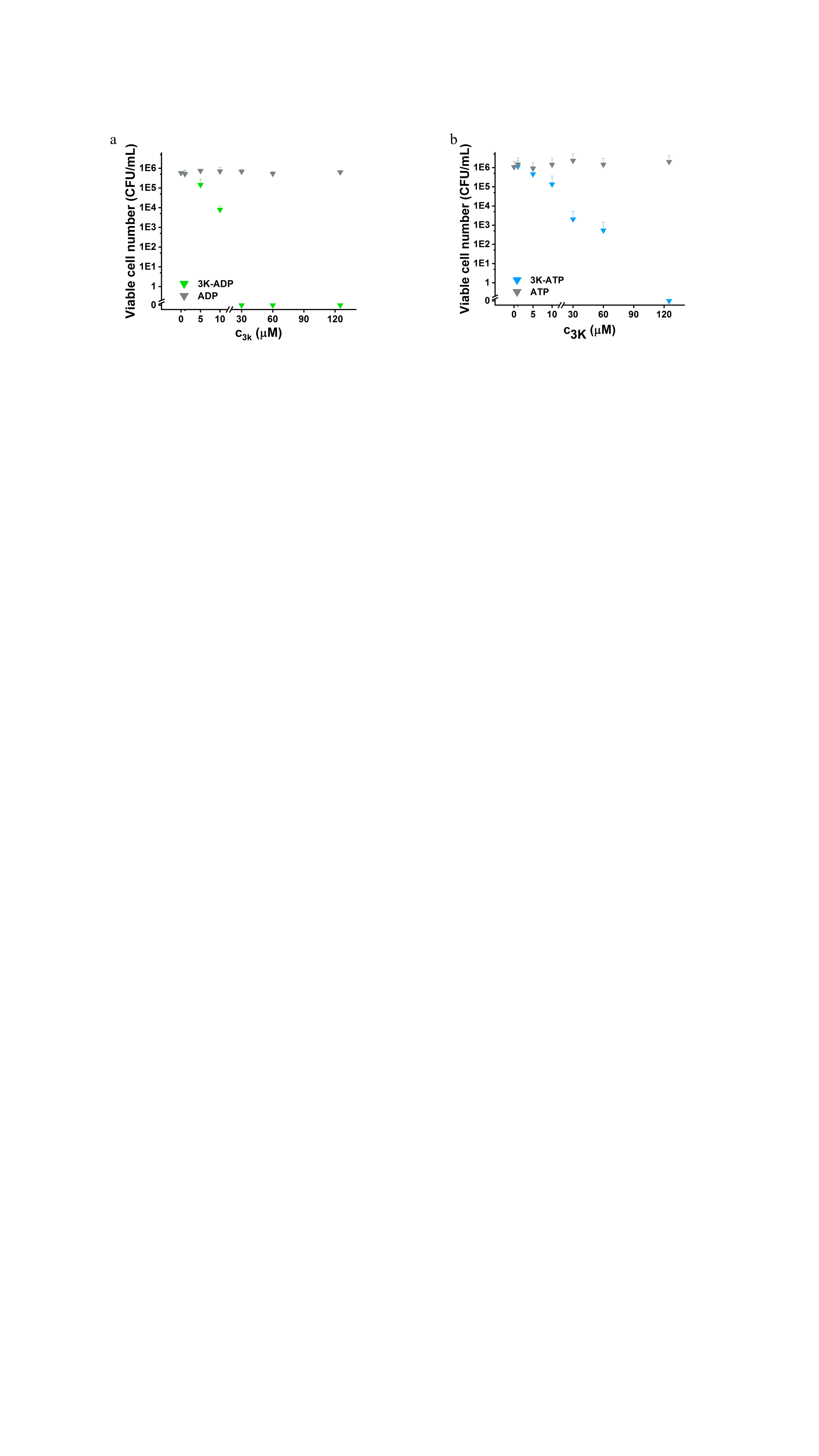


**Supplementary Figure 11.**  **Antibacterial activity of 3K-APs**. Raw CFU data for antibacterial activities of a) 3K-ADP and b) 3K-ATP in water. The concentration ratio 3K to AP is 1:4. Data represent mean of 3 biological replicates. All errors are SEM. The calculated values of the fit are listed in Supplementary Table 3 and viability is presented in Figure 2a.

**Supplementary Table 3.** **Antibacterial activity of 3K-ATP and 3K-ADP.** The concentration ratio 3K to AP is 1:4. Concentrations given correspond to that of 3K. The data represent 3 biological replicates. All errors are SEM.

| **Compound** | **IC_20_ [µM]** | **IC_50_ [µM]** | **IC_80_ [µM]** |
| --- | --- | --- | --- |
| 3K-ADP | 2.90 ± 0.30 | 3.86 ± 0.31 | 5.14 ± 0.31 |
| 3K-ATP | 3.11 ± 0.46 | 4.64 ± 0.56 | 6.92 ± 0.66 |

**Minimum inhibitory concentration of 3K-ATP on *E. coli* BL21 DE3**


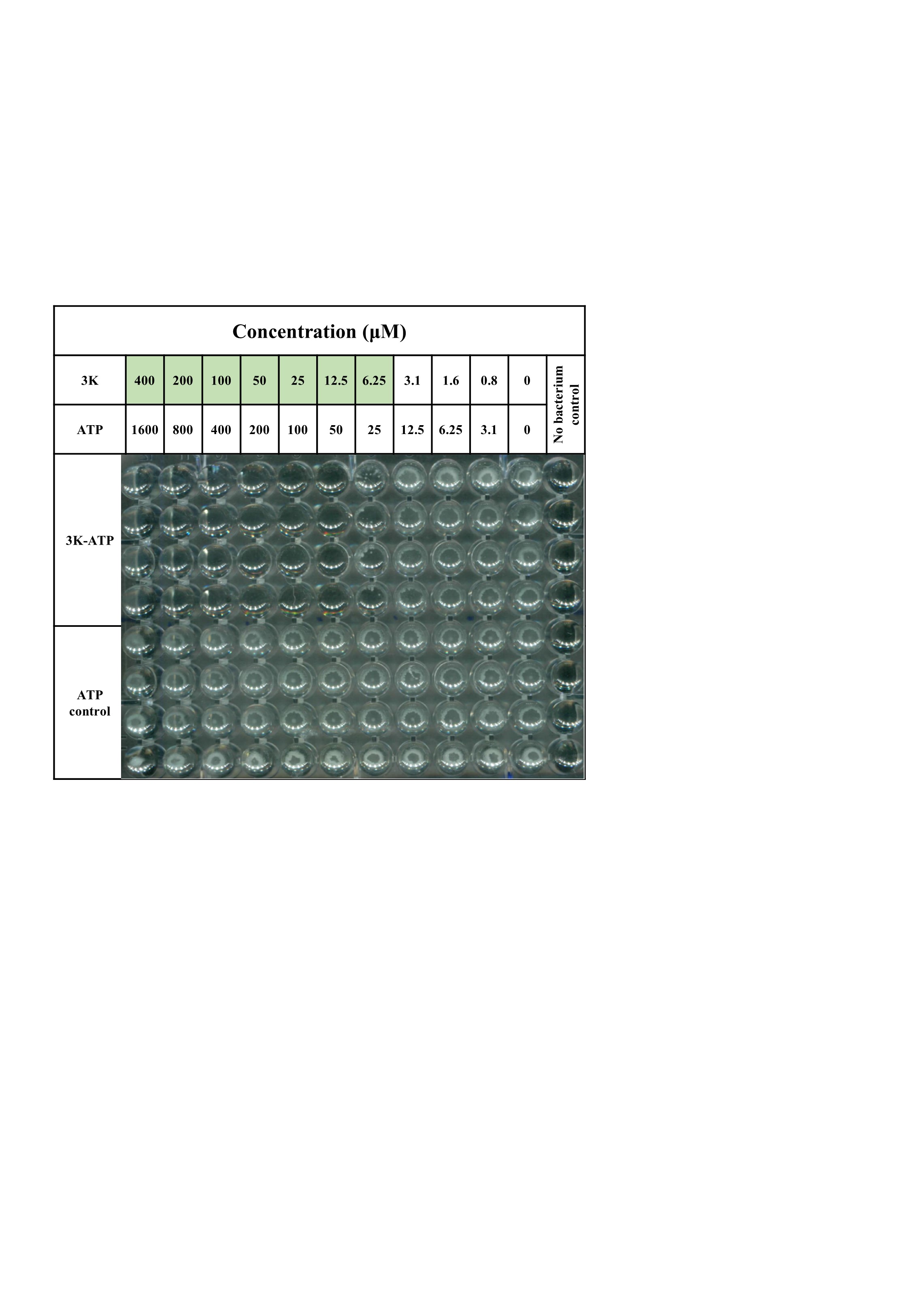


**Supplementary Figure 12. Minimum inhibitory concentration (MIC) determination using the EUCAST microdilution protocol.** MICs for 3K-ATP (ratio 1:4) against the *E. coli* laboratory strain BL21 DE3 were assessed by visible turbidity changes. Each well was inoculated with 5 ± 3 × 10⁵ bacterial cells/mL in a final volume of 100 µL. The plates were incubated at 37 °C for 20 hours without shaking.

**Minimum inhibitory concentration of 3K-ATP against antibiotic-adapted lines and multidrug-resistant *E. coli* strains.**

To further investigate the role of membrane-related mutations in 3K-ATP susceptibility, we examined *E. coli* ATCC 25922 strains adapted to membrane-targeting antibiotics, as described in a previously published evolution experiment.^13^

In this study, bacterial populations were exposed to polymyxin B (PMB), POL7306 (POL), or tridecaptin M (TRD) over approximately 120 generations (Supplementary Figure 14). These antibiotics target different components of the bacterial membrane, including lipopolysaccharide (LPS), BamA, and lipid II. During adaptation, the evolved lines exhibited a 128-fold increase in resistance to PMB and an 8- to 16-fold increase in resistance to TRD and POL, relative to the ancestral strain.^14^

Given the membrane-targeting nature of these antibiotics, we hypothesized that adaptation might affect susceptibility to 3K-ATP. MIC testing of the evolved lines revealed values of 100 µM or lower, corresponding to only a two-fold reduction in susceptibility compared to the ancestral strain, indicating limited cross-resistance. Interestingly, TRD-adapted lines harboring mutations in the *wbbD* gene showed increased sensitivity to 3K-ATP. *wbbD* encodes a galactosyltransferase involved in the biosynthesis of the O7-polysaccharide repeating unit of LPS, suggesting that alterations in LPS structure may enhance the activity of 3K-ATP.^15^

3K-ATP also demonstrated notable activity against multidrug-resistant Escherichia coli strains. MIC values remained below 100 µM for β-lactamase-producing isolates (E. coli ATCC BAA-2469 and E. coli ATCC BAA-2340), both of which are listed among the highest priority pathogens by the World Health Organization (WHO).^16^ Furthermore, E. coli strains carrying the mcr-1 gene, associated with lipid A modification and colistin resistance, were also tested. Importantly, the presence of mcr-1 did not substantially reduce the efficacy of 3K-ATP, as the MIC remained at 50 µM, indicating that this resistance mechanism does not significantly impair its antibacterial activity.


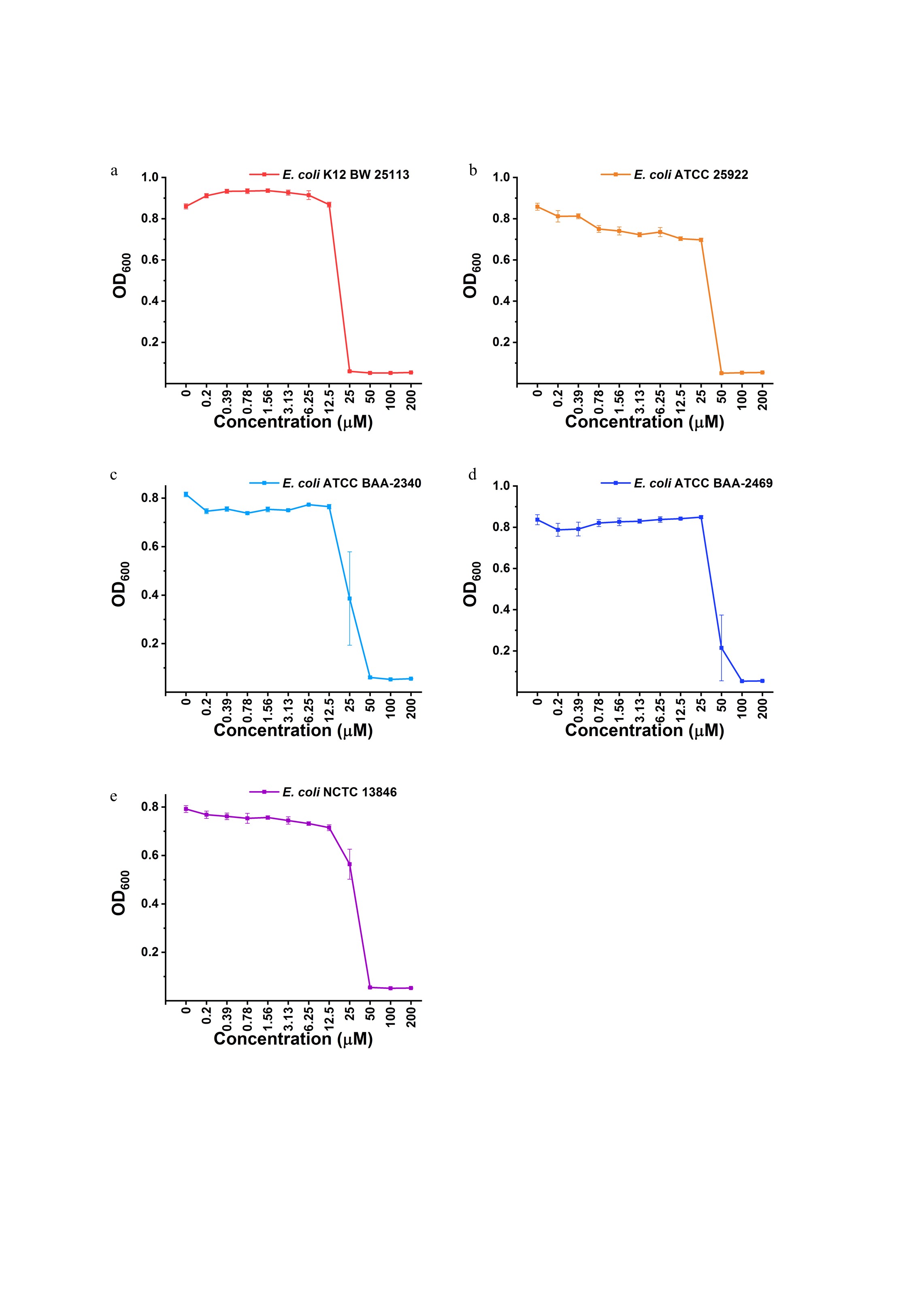


**Supplementary Figure 13. Minimum inhibitory concentrations (MICs) of 3K-ATP against pathogenic *E. coli* strains**. MICs of 3K-ATP (ratio 1:4) were determined against a panel of pathogenic *Escherichia coli* strains, including E. coli K12 BW 25113 and E. coli ATCC 25922 in LB medium (a, b). Bacterial growth was assessed by measuring optical density at 600 nm (OD_600_). For better readability the graphs are presented with uniformly spaced data points along the x axis, regardless of their actual x values. The concentrations on the x axis correspond to the 3K concentration within the 3K-ATP complex.


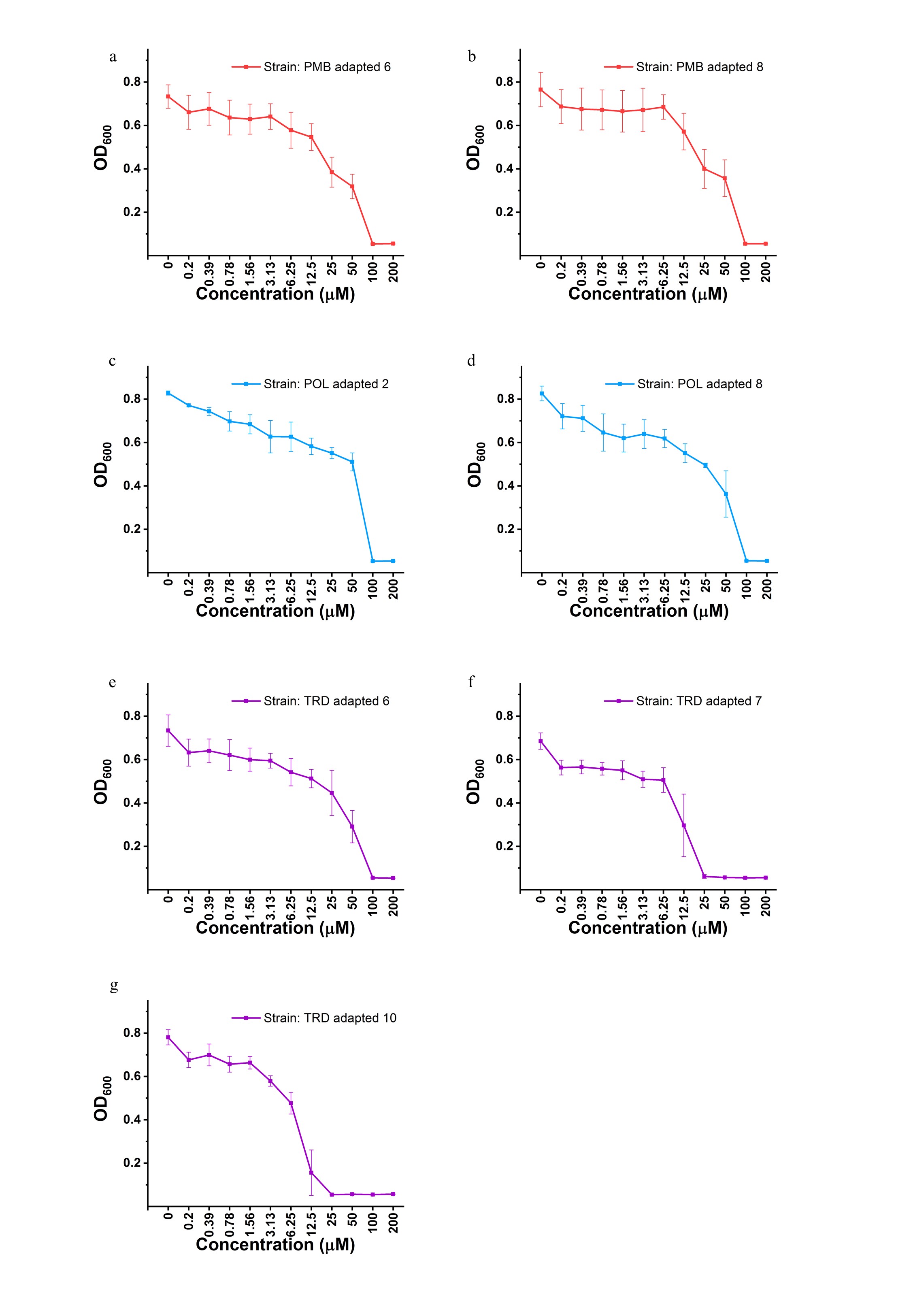


**Supplementary Figure 14. MICs of 3K-ATP (ratio 1:4) against *E. coli* ATCC 25922 strains adapted to membrane-targeting antibiotics (PMB, POL and TRD) in LB medium.** Bacterial growth was assessed by measuring optical density at 600 nm (OD_600_). For better readability the graphs are presented with uniformly spaced data points along the x axis, regardless of their actual x values. The concentrations on the x axis correspond to the 3K concentration within the 3K-ATP complex.

**
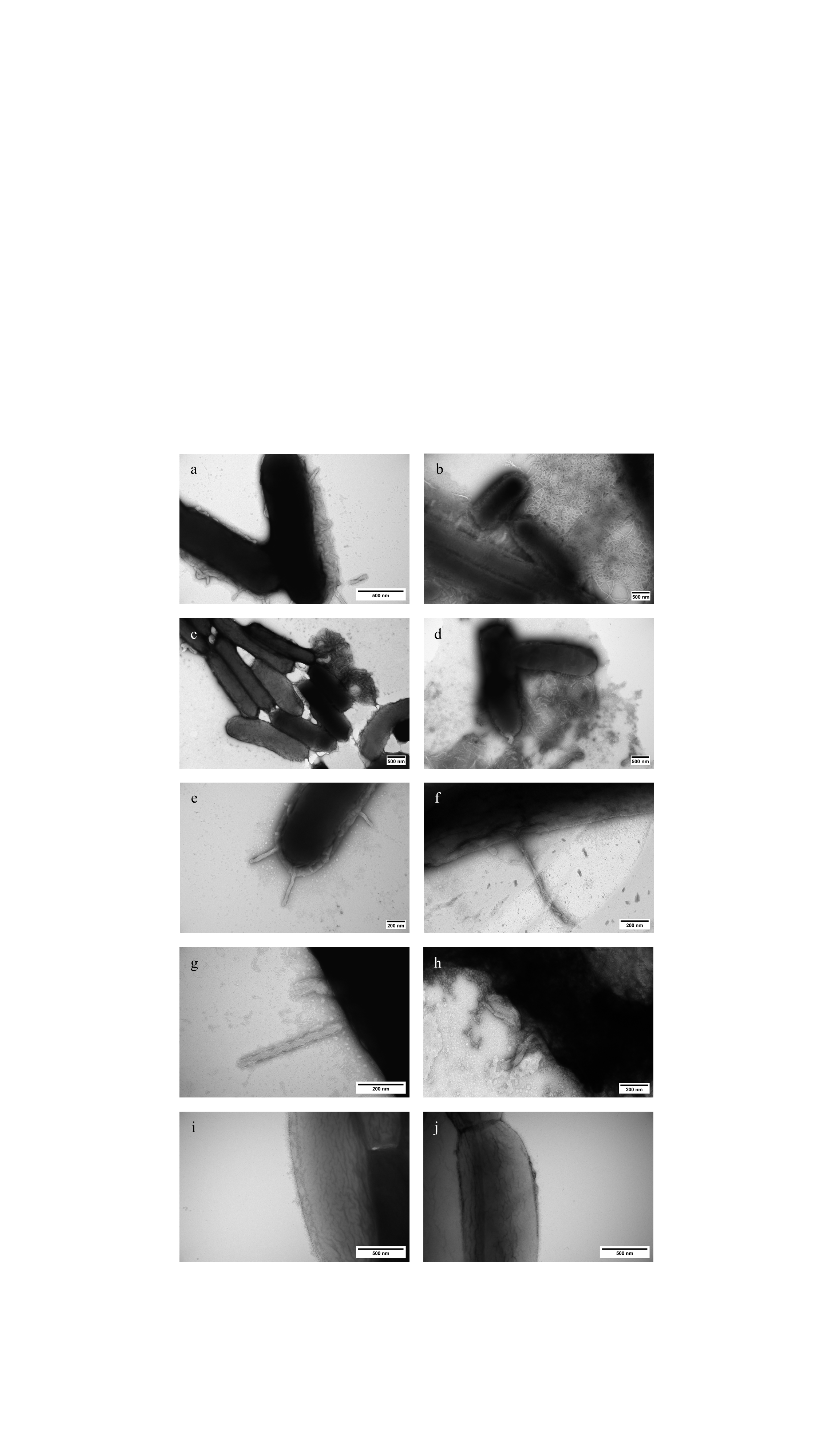
**

**Supplementary Figure 15. Transmission electron microscopy reveals time-dependent morphological evolution of 3K-AP assemblies in contact with *E. coli*.** TEM images of *E. coli* cells treated with 3K-ATP and 3K-ADP for a) and b) 20 min, c) and d) 40 min, e) and f) 60 min, g) and h) 80 min respectively. i) and j) *E. coli* cells treated with ATP and ADP alone at 20 min (controls) respectively. The concentration ratio 3K to AP was 1:4 (20 μM 3K and 80 μM AP).

**
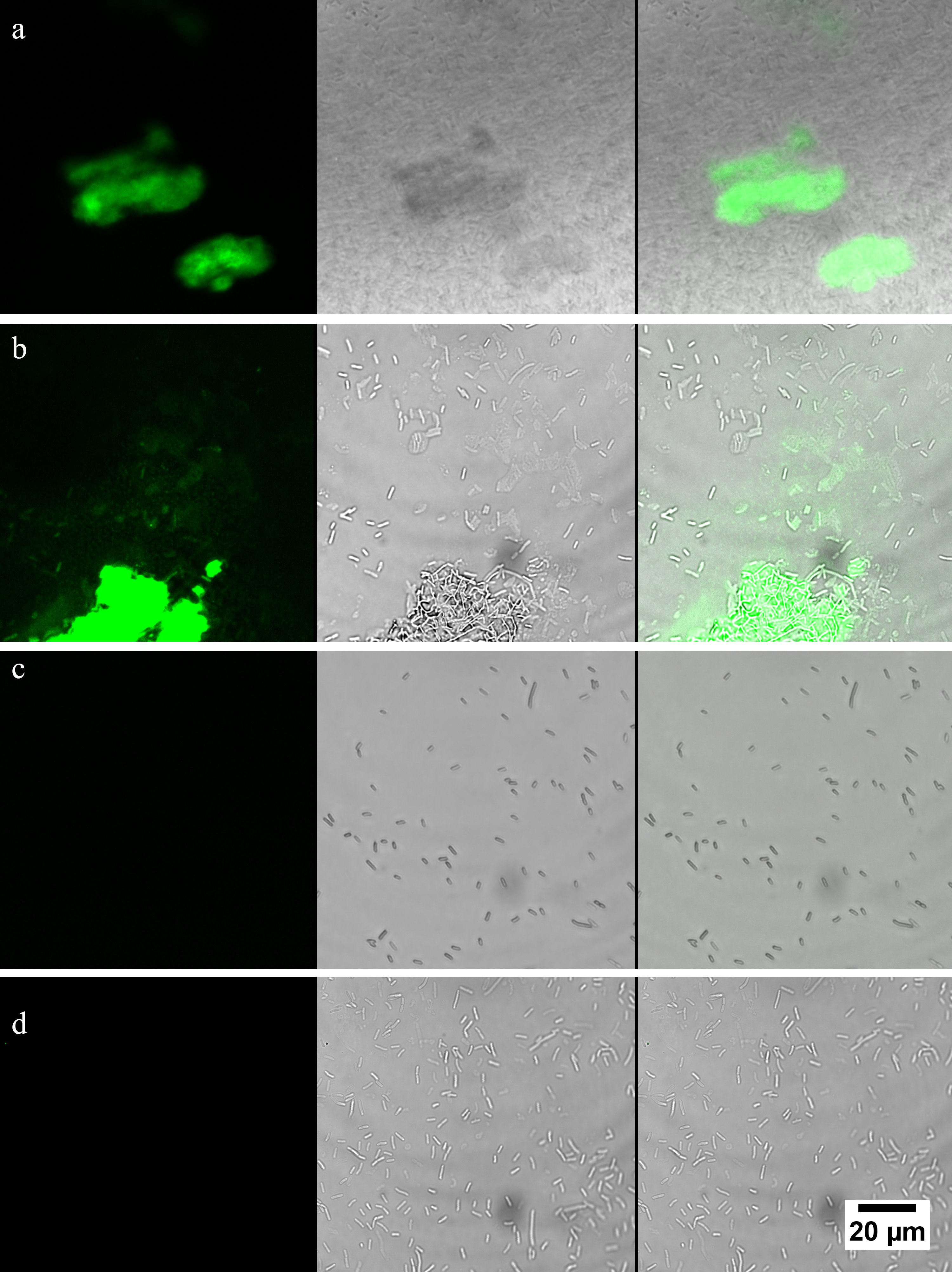
**

**Supplementary Figure 16. Visualization of the bacterial colony agglutination induced by the 3K-ATP coassemblies.** Confocal microscopy images of *E. coli* treated with a) 3K-1% fluoATP (fluorescently labelled ATP), 30 min, b) 3K-0.1% fluoATP, 60 min, c) 3K-0,1% fluoATP, 0 min, d) 1% fluoATP, 30 min (control). The signal of fluoATP is shown in green (1st column), *E. coli* are visualized via transmitted light (2nd column). Merged images are presented in the third column. The concentration ratio 3K to AP was 1:4 (20 μM 3K and 80 μM AP).


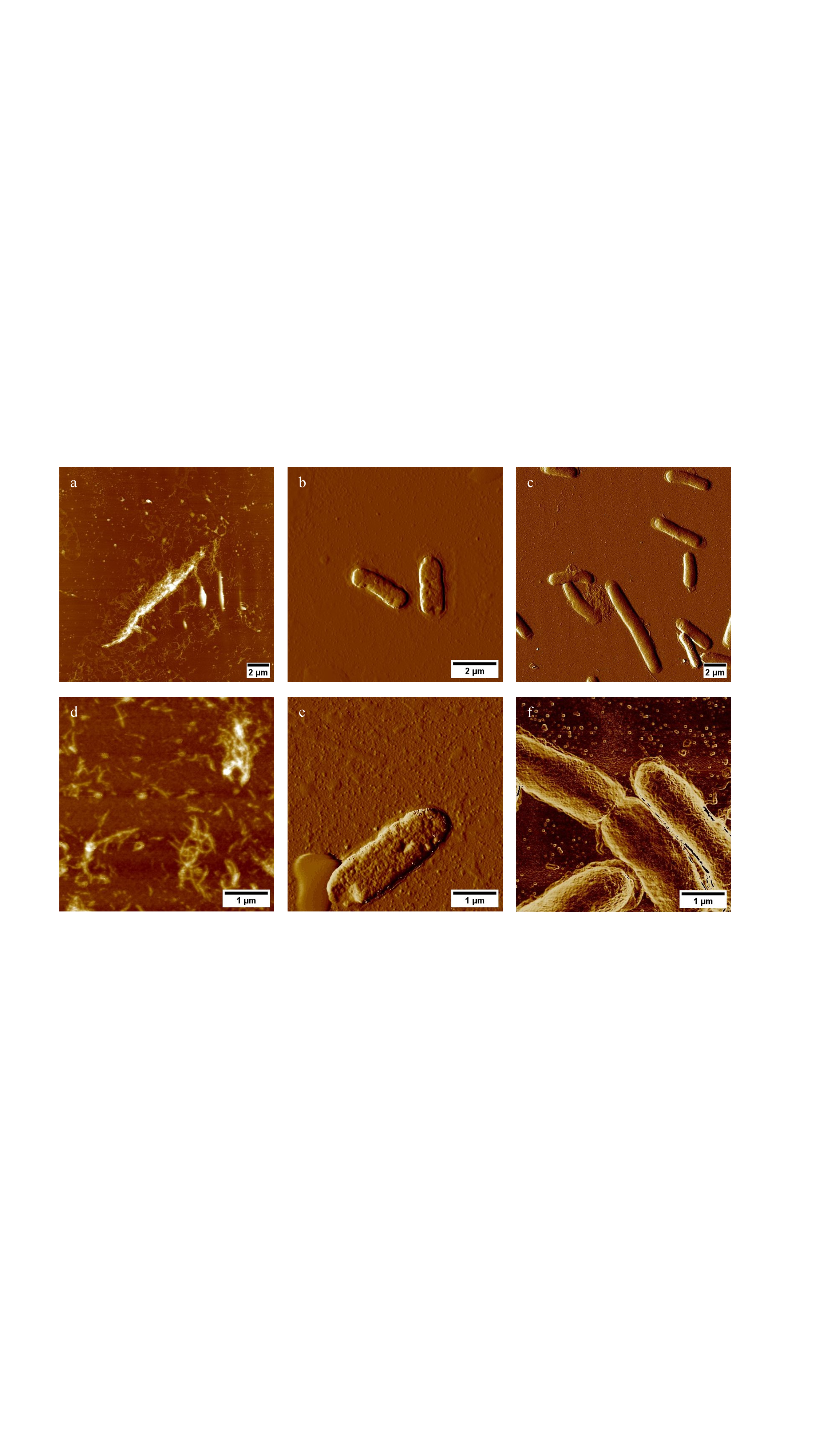


**Supplementary Figure 17. AFM imaging of 3K-ATP assemblies and their interaction with *E. coli*.** Tapping mode AFM height and amplitude images at two different magnifications of 3K-ATP assemblies (20 μM 3K and 80 μM ATP) (a, d), untreated *E. coli* cells (b, e), and *E. coli* cells treated with 3K-ATP (c, f). The 3K-ATP assemblies are clearly visible and appear to wrap around the bacterial cells.


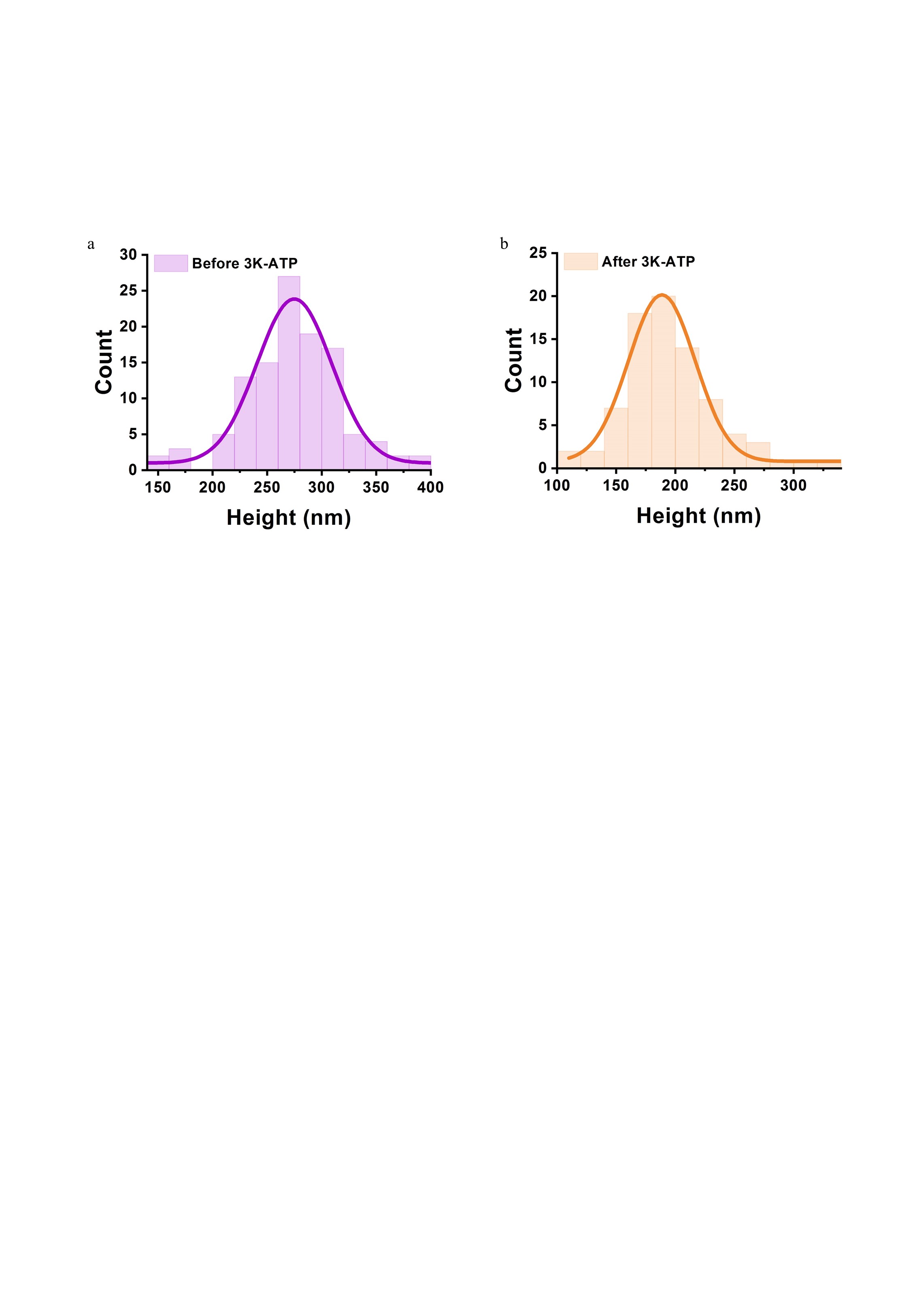


**Supplementary Figure 18. Height reduction of *E. coli* cells following 3K-ATP treatment.** Height statistics of individual *E. coli* cells. before (a) and after (b) 3K-ATP treatment. 38 untreated and 27 treated cells have been analysed, with three measurements taken per cell (front, middle, and end). A Mann–Whitney U test performed on the two datasets confirmed that the difference is statistically significant at the 95% level.

**Supplementary Table 4. Median height of individual *E. coli* cells before and after 3K-ATP treatment**. Values are obtained from data shown in the Supplementary Figure 18. Upon 3K-ATP treatment, a reduction of 30.1% in median cell height was observed.

| 3K-ATP treatment | Median height (nm) |
| --- | --- |
| Before treatment | 275.09 |
| After treatment | 192.18 |

a

c


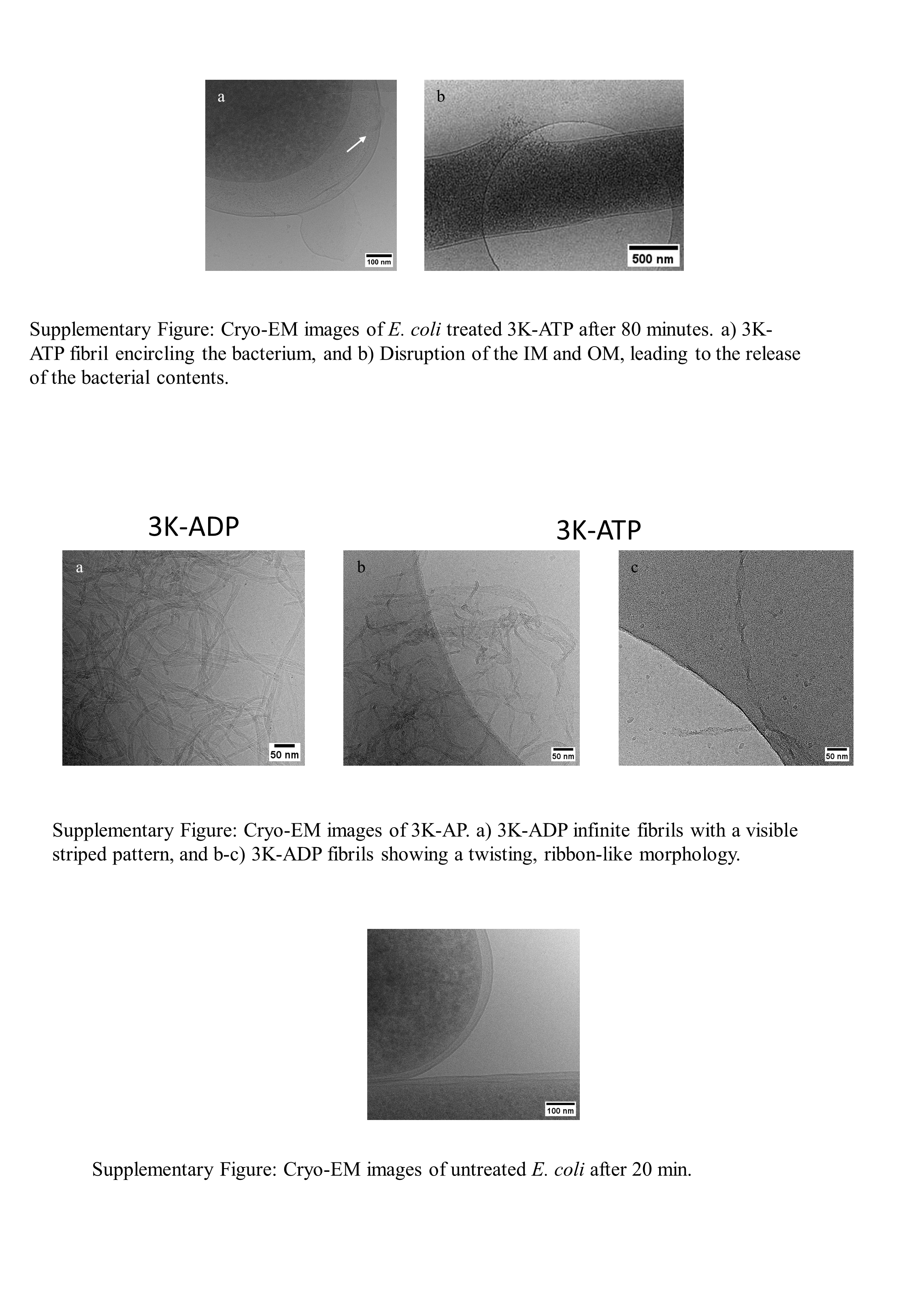


**Supplementary Figure 19.** **Cryo-EM images of 3K-AP (20 μM:80 μM).** a) 3K-ADP infinite fibrils with a visible striped pattern, and b-c) 3K-ADP fibrils showing a twisting, ribbon-like morphology.


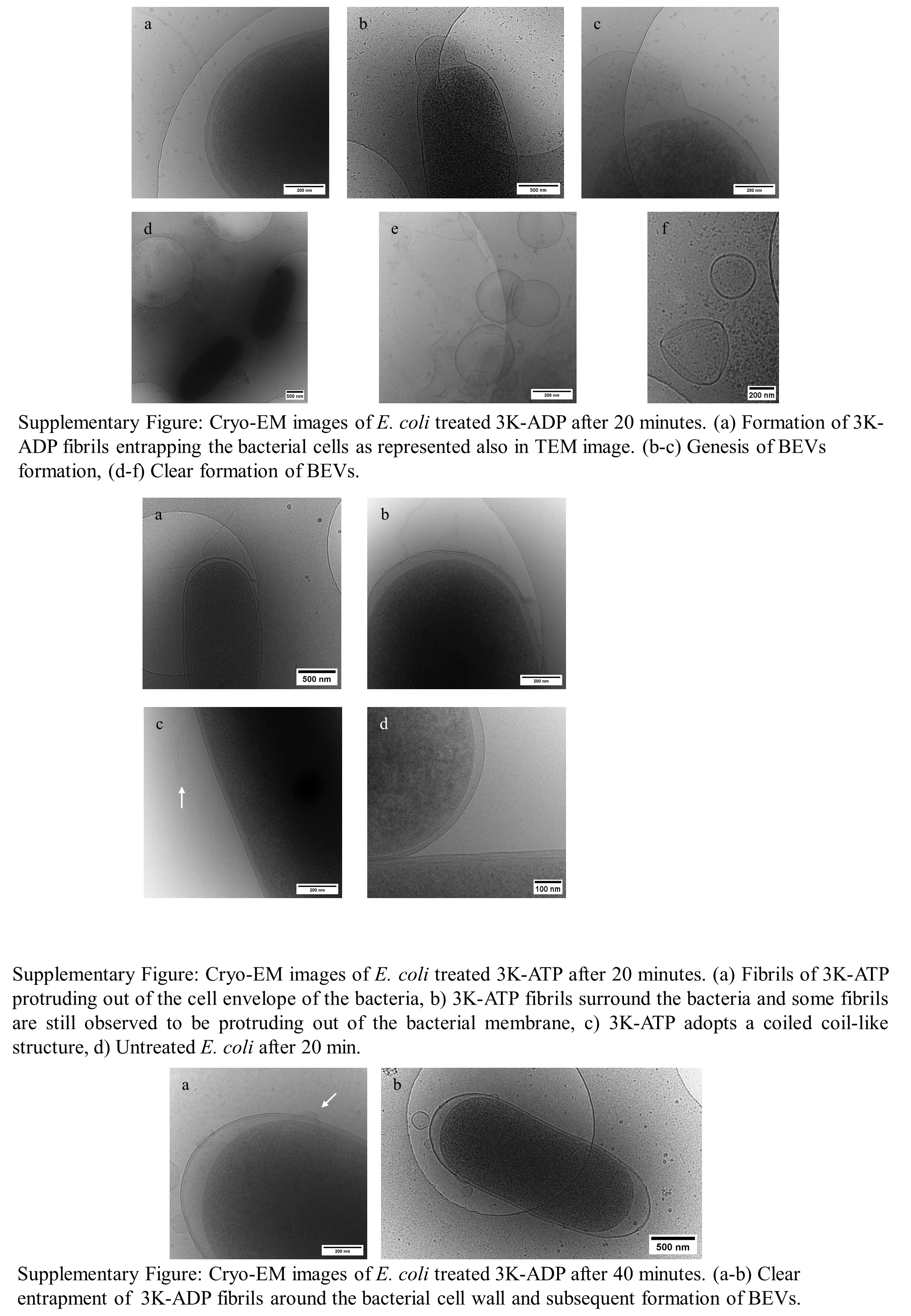


**Supplementary Figure 20.** **Cryo-EM images of *E. coli* treated with 3K-ADP for 20 minutes.** The concentration ratio 3K to AP was 1:4 (20 μM 3K and 80 μM AP) (a) Formation of 3K-ADP fibrils entrapping the bacterial cells was observed also in NS-TEM images. (b-c) BEV generation, the vesicles are shown during formation, (d-f) already formed and released spherical BEVs.


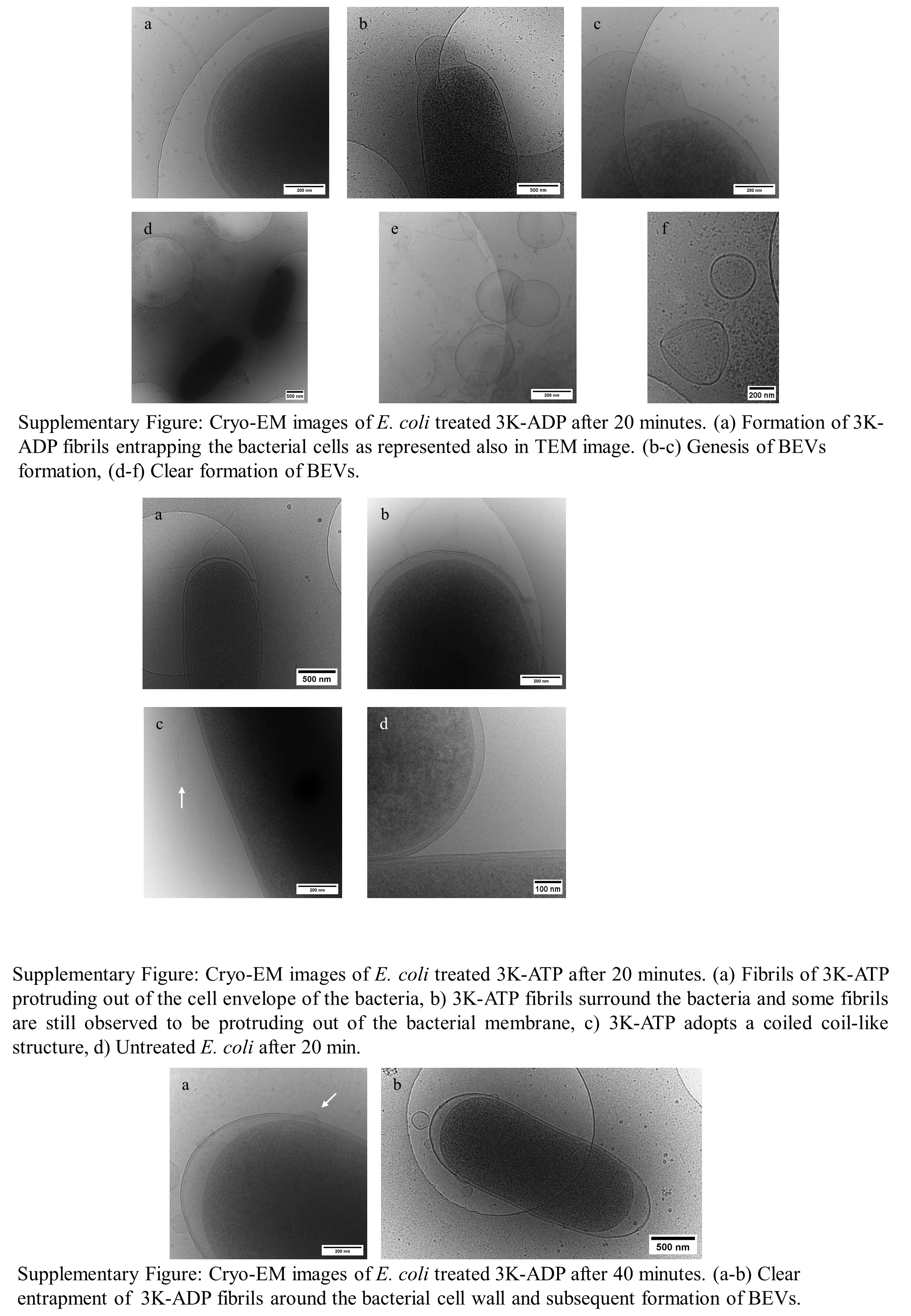


**Supplementary Figure 21.** **Cryo-EM images of *E. coli* treated 3K-ATP after 20 minutes.** a) Fibrils of 3K-ATP protruding out of the cell envelope of the bacteria, b) 3K-ATP fibrils surround the bacteria and some fibrils are still observed to be protruding out of the bacterial membrane, c) 3K-ATP adopts a coiled coil-like structure, d) Untreated *E. coli* after 20 min incubation. The concentration ratio 3K to AP was 1:4 (20 μM 3K and 80 μM AP).


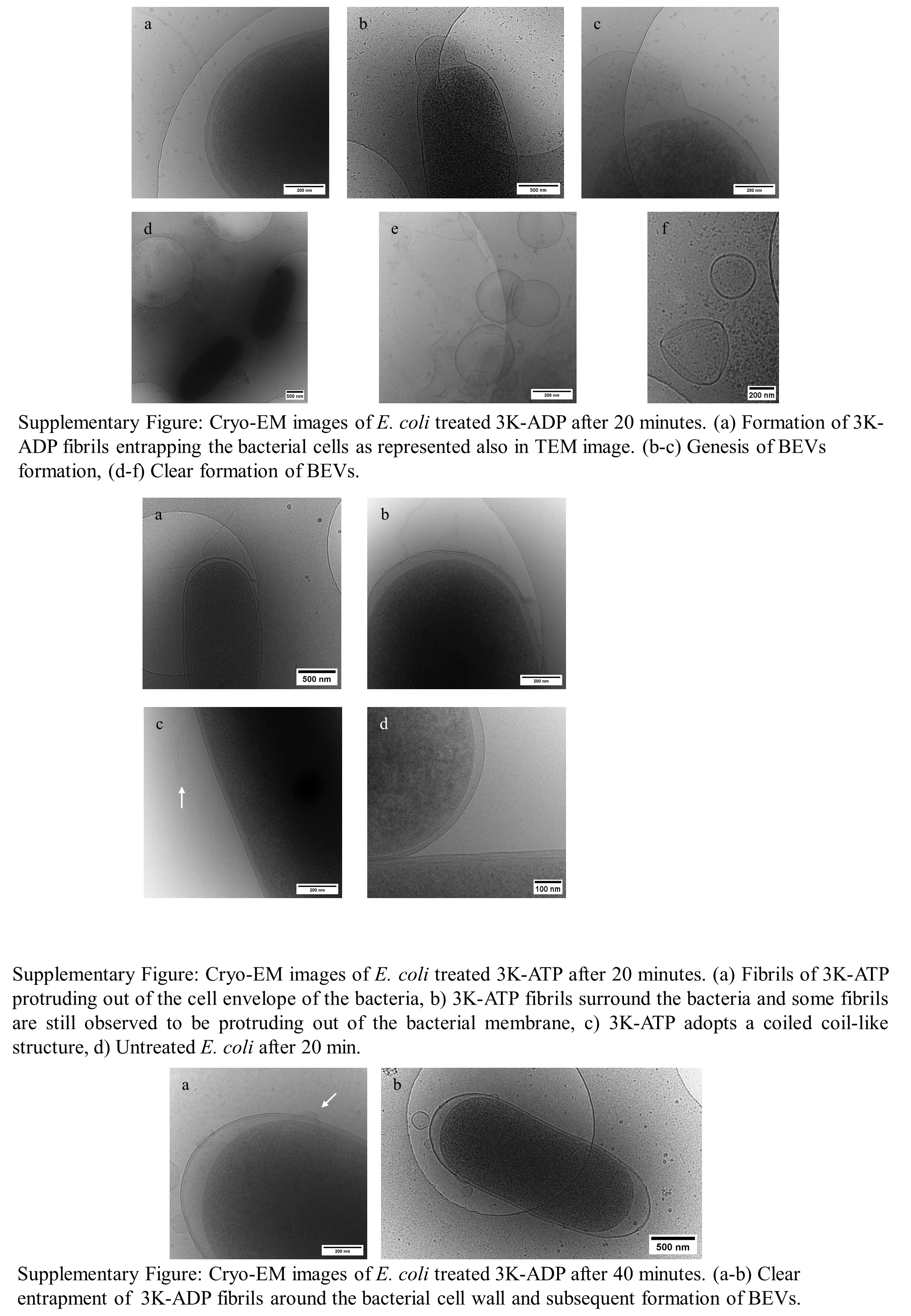


**Supplementary Figure 22. Cryo-EM images of *E. coli* treated 3K-ADP after 40 minutes.** (a-b) Clear entrapment of 3K-ADP fibrils around the bacterial cell wall and formation of BEVs. The concentration ratio 3K to AP was 1:4 (20 μM 3K and 80 μM AP).


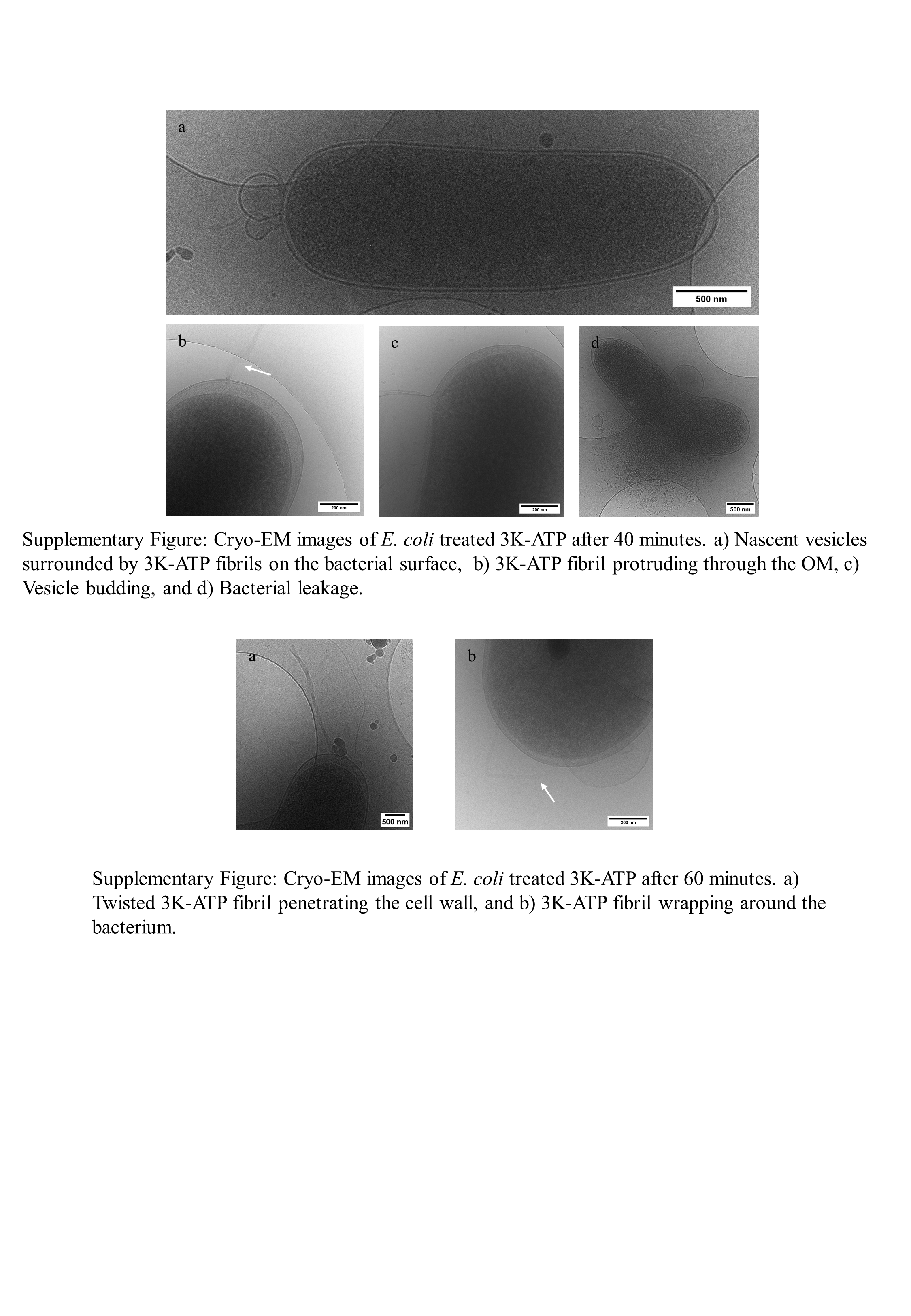


**Supplementary Figure 23.** **Cryo-EM images of *E. coli* treated 3K-ATP after 40 minutes.** a) Nascent vesicles surrounded by 3K-ATP fibrils on the bacterial surface, b) 3K-ATP fibril protruding through the OM, c) Vesicle budding, and d) Bacterial leakage. The concentration ratio 3K to AP was 1:4 (20 μM 3K and 80 μM AP).


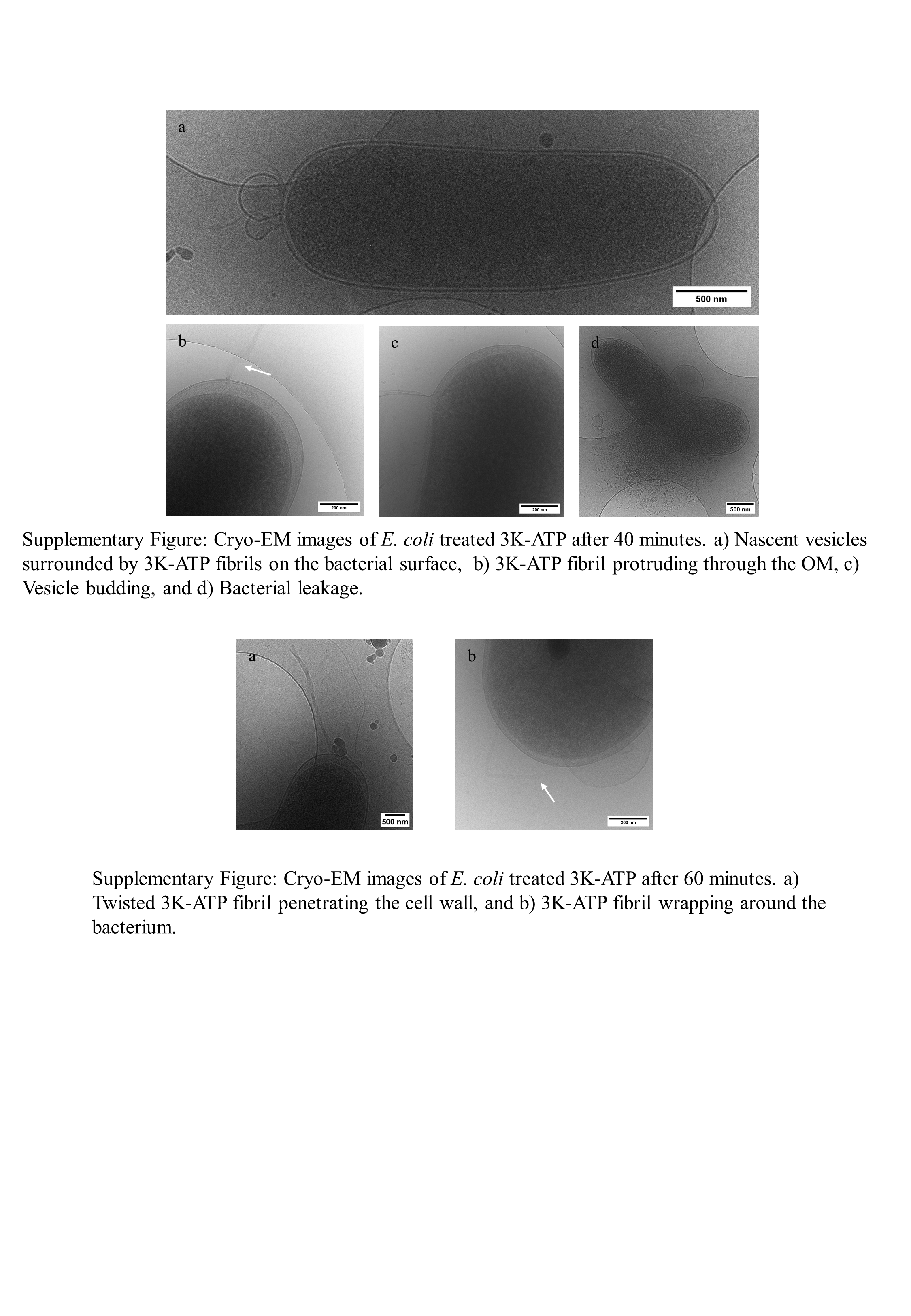


**Supplementary Figure 24.** **Cryo-EM images of *E. coli* treated 3K-ATP after 60 minutes.** a) Twisted 3K-ATP fibril penetrating the cell wall, and b) 3K-ATP fibril wrapping around the bacterium. The concentration ratio 3K to AP was 1:4 (20 μM 3K and 80 μM AP).


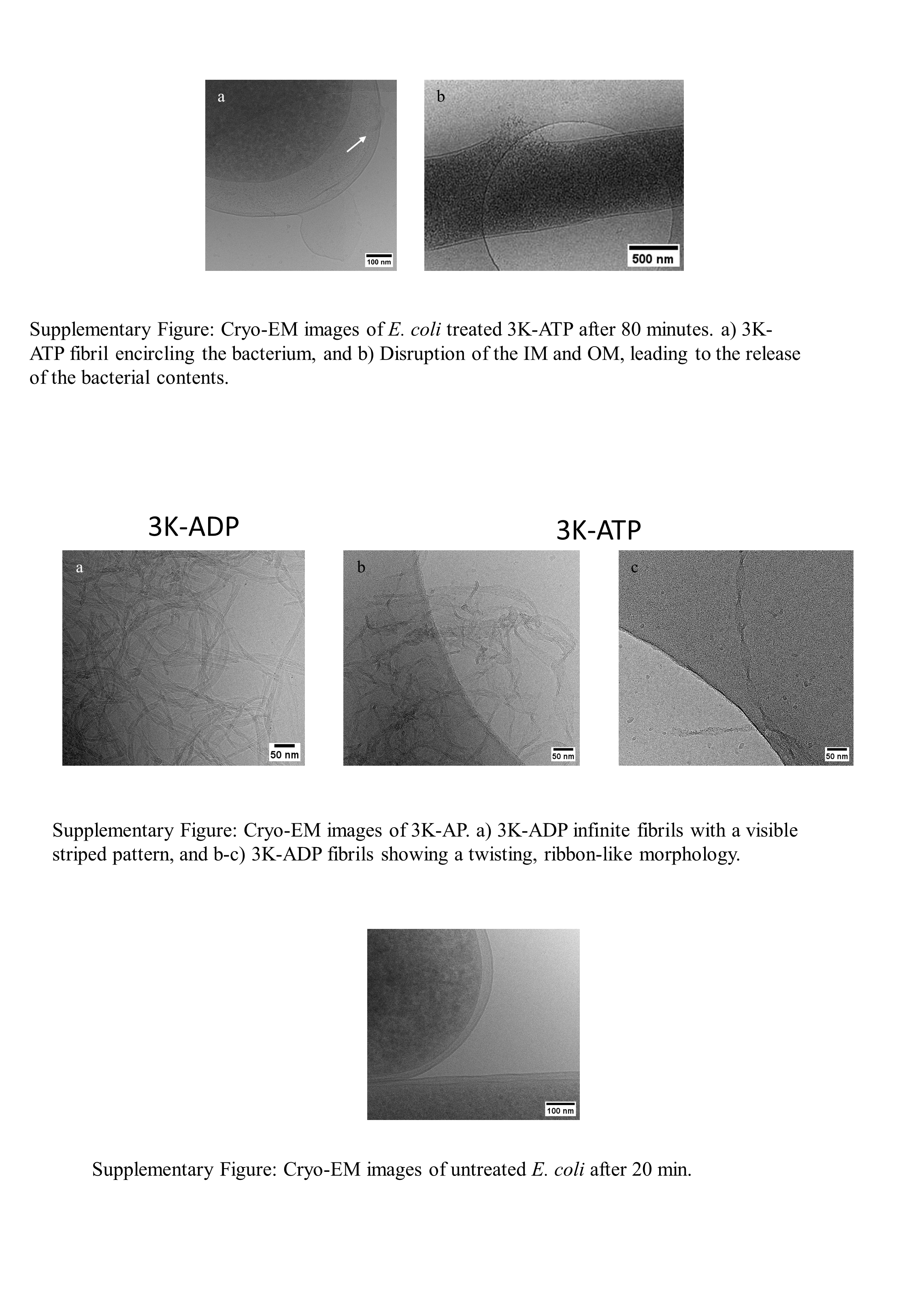


**Supplementary Figure 25.** **Cryo-EM images of *E. coli* treated 3K-ATP after 80 minutes.** a) 3K-ATP fibril encircling the bacterium, and b) Disruption of the IM and OM, leading to the release of the bacterial contents. The concentration ratio 3K to AP was 1:4 (20 μM 3K and 80 μM AP).


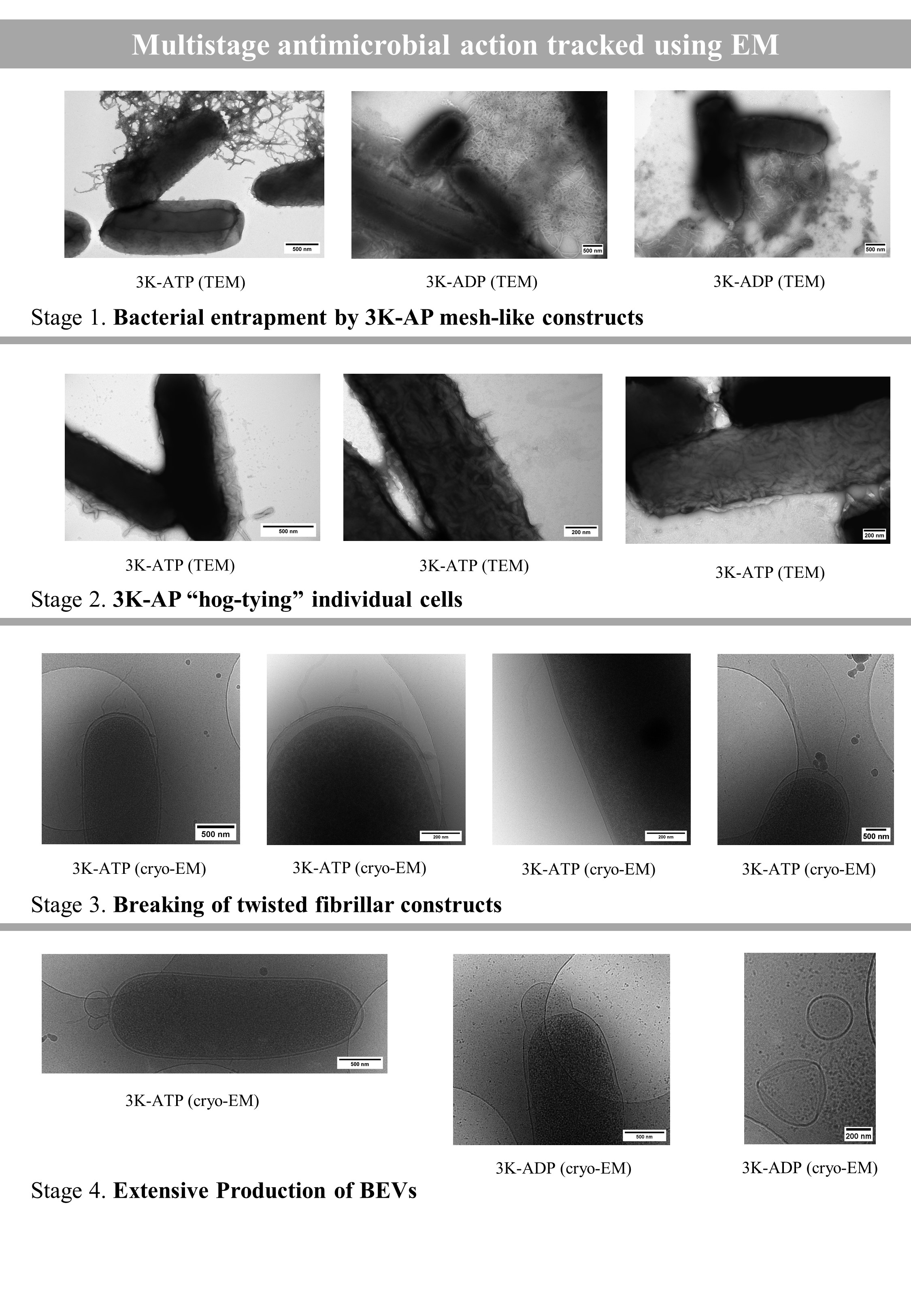


**Supplementary Figure 26. Overview of the multistage antimicrobial mechanism of 3K-AP co-assemblies tracked via EM techniques.** The first stage is initiated by the bacterial entrapment, caused by the 3K-AP mesh-like construct. In stage 2, 3K-AP wraps around the bacterial surface and “hog-ties” the individual cells. The 3K-AP assemblies adopt a twisted fibrillar morphology and anchor to the bacterial outer membrane. The stage 4 depicts the production of BEVs and the disruption of the bacteria cell wall.

**Effect of 3K-ATP treatment on red blood-derived extracellular vesicles (REVs)**

Red blood cell derived EVs (REVs) isolation

Ethical approval for using human blood samples was obtained from the Scientific and Research Ethics Committee of the Hungarian Medical Research Council (ETT TUKEB IV/701-3/2022/EKU). The investigations were carried out on consensual healthy donors, following the guidelines and regulations of the Declaration of Helsinki. A 12 mL blood sample was collected from healthy male donors aged between 27 to 45 years in tripotassium ethylenediaminetetraacetic acid (K3EDTA) tubes (Greiner Bio-One International GmbH). To isolate red blood cells (RBCs), the blood sample was centrifuged at 800 *g* for 10 minutes at 4 °C using an Eppendorf 5810R centrifuge (A-4-62 model with adapters for 15 mL centrifuge tubes). The RBCs were collected, and the supernatant was discarded. The RBCs were then washed with a physiological salt solution, and this wash step was repeated at least three times, ensuring the removal of platelets and the buffy coat. The isolated RBCs were resuspended in phosphate-buffered saline (PBS 1x, pH 7.4, 0.2 μm filtered) and stored for a 7-day incubation period to produce red blood cell-derived extracellular vesicles (REVs). After incubation, the sample underwent a centrifugation step at 2500 *g* for 15 minutes and then at 3000 *g* for 30 minutes to remove cells and cellular debris. To isolate the REVs, the supernatant was centrifuged at 16 000 *g* for 30 minutes at 4 °C using an Eppendorf 5415R centrifuge (with an adapter for 2 mL tubes). The pellets containing the REVs were then resuspended in 100 μL of PBS.

Polarized light spectroscopy (Linear Dichroism)

Linear dichroism (LD) is a phenomenon observed in systems that are either intrinsically oriented or can be aligned by an external force. LD is defined as the difference in absorption (A) of light that is linearly polarized parallel (//) and perpendicular (⊥) to an orientation axis within such a system.

$$LD=A_{\parallel}-A_{\perp} . (1)$$

For the study, a shear gradient was generated using a Couette flow cell, which altered the spherical shape of extracellular vesicles (EVs) into a distorted ellipsoid. This alteration oriented the chromophores relative to the macroscopic orientation axis of the system. Extracellular vesicles (REVs) were diluted in PBS (1x, pH 7.4, filtered through a 0.2 μm filter). A 90 μL aliquot of the 3-fold diluted sample was then transferred into a circular quartz Couette cell.

For the experiments, 3K-ATP was titrated into the Couette cell at increasing concentrations (0 µM, 5 µM, 10 µM, 20 µM, and 40 µM). LD measurements were conducted at room temperature using a JASCO-1500 spectropolarimeter equipped with a Couette flow cell system (CFC-573). Spectra were collected from 200 to 600 nm at a rate of 100 nm/min, with a data pitch of 0.5 nm, a response time of 1 s, and a bandwidth of 1 nm. The samples were oriented under a shear gradient of 2270 s^-1^. Baselines at zero shear gradient were measured and subtracted from the results.

### Flow Cytometry Analysis (FCM)

### A 13-color, 3-laser CytoFLEX s V4-B2-Y0-R3 flow cytometer, equipped with 405 nm, 488 nm, and 638 nm lasers (Beckman Coulter, USA), was utilized and operated using CytExpert Software v2.5 (Beckman Coulter, Brea, CA). For measuring single extracellular vesicles (EVs), the configuration was set to use Violet-Side Scatter (VSSC) detection. VSSC served as the trigger channel, with a height threshold of 1500 arbitrary units. The gain settings for the violet SSC, FITC, and PE fluorescence channels were set to 30, 40, 250, and 50, respectively. Samples were analyzed for 60 seconds at a flow rate of 10 µL/min.

### 3K-ATP was added to the REVs at concentrations of 0 µM, 5 µM, 10 µM, 20 µM, and 40 µM, after which they underwent a 7000-fold dilution. Data processing was performed using Kaluza analysis software from Beckman Coulter. Three individual measurements were conducted for the selected peptides, and mean values along with standard deviations were calculated for each peptide.


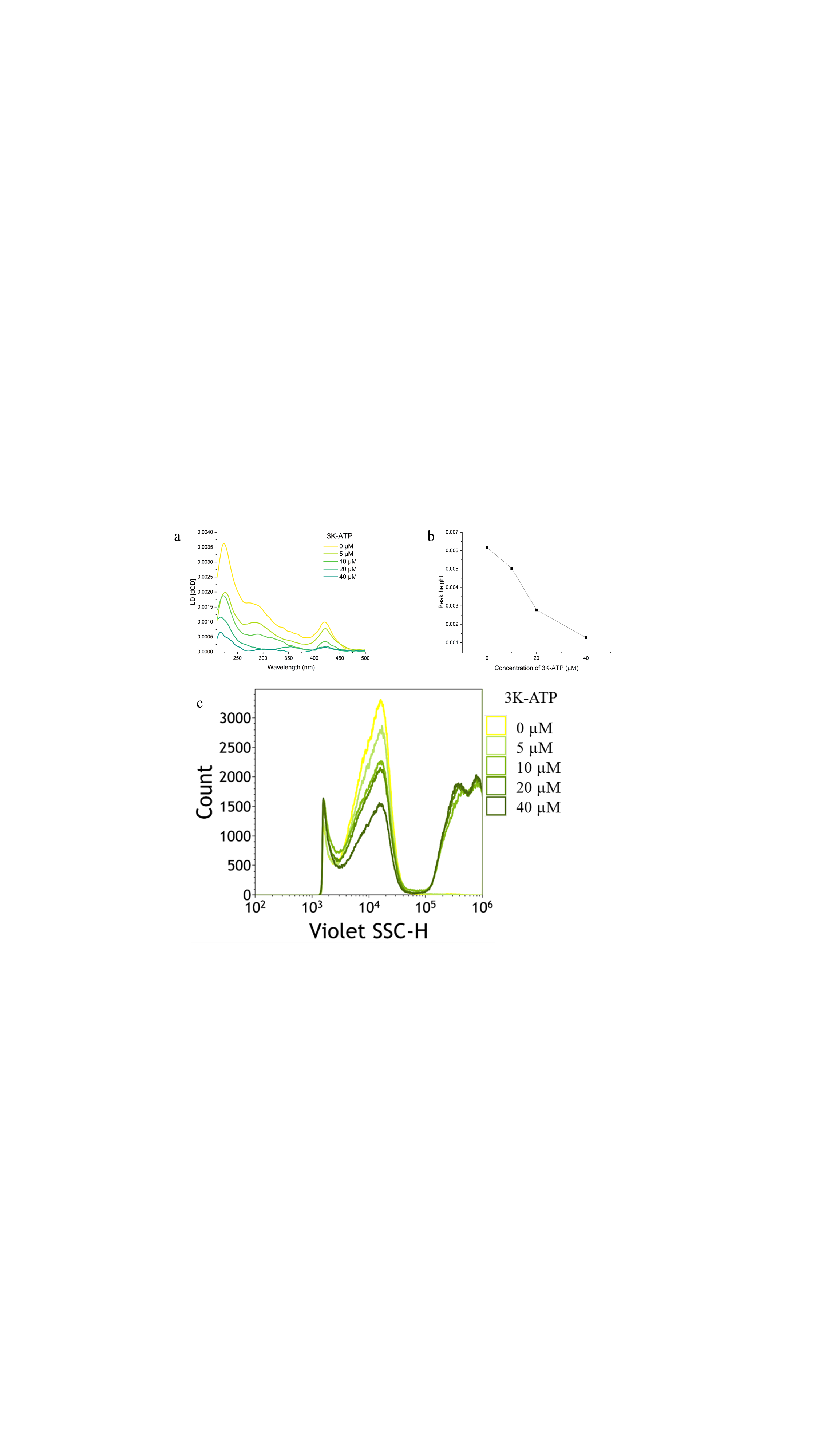


**Supplementary Figure 27. Effect of 3K-ATP treatment on red blood-derived extracellular vesicles (REVs).** a) Linear Dichroism (LD) spectra of the REVs upon subsequent addition of 3K-ATP. Control REVs (yellow) display two main peaks, notably the Soret band at ∼420 nm, arising from the heme groups of surface-attached hemoglobin (Hb). 3K-ATP treatment results in signal loss of both signals. b) LD Soret band intensity changes upon addition of 3K-ATP. c) Flow cytometry analysis showing lytic trends in REVs upon addition of 3K-ATP.

**
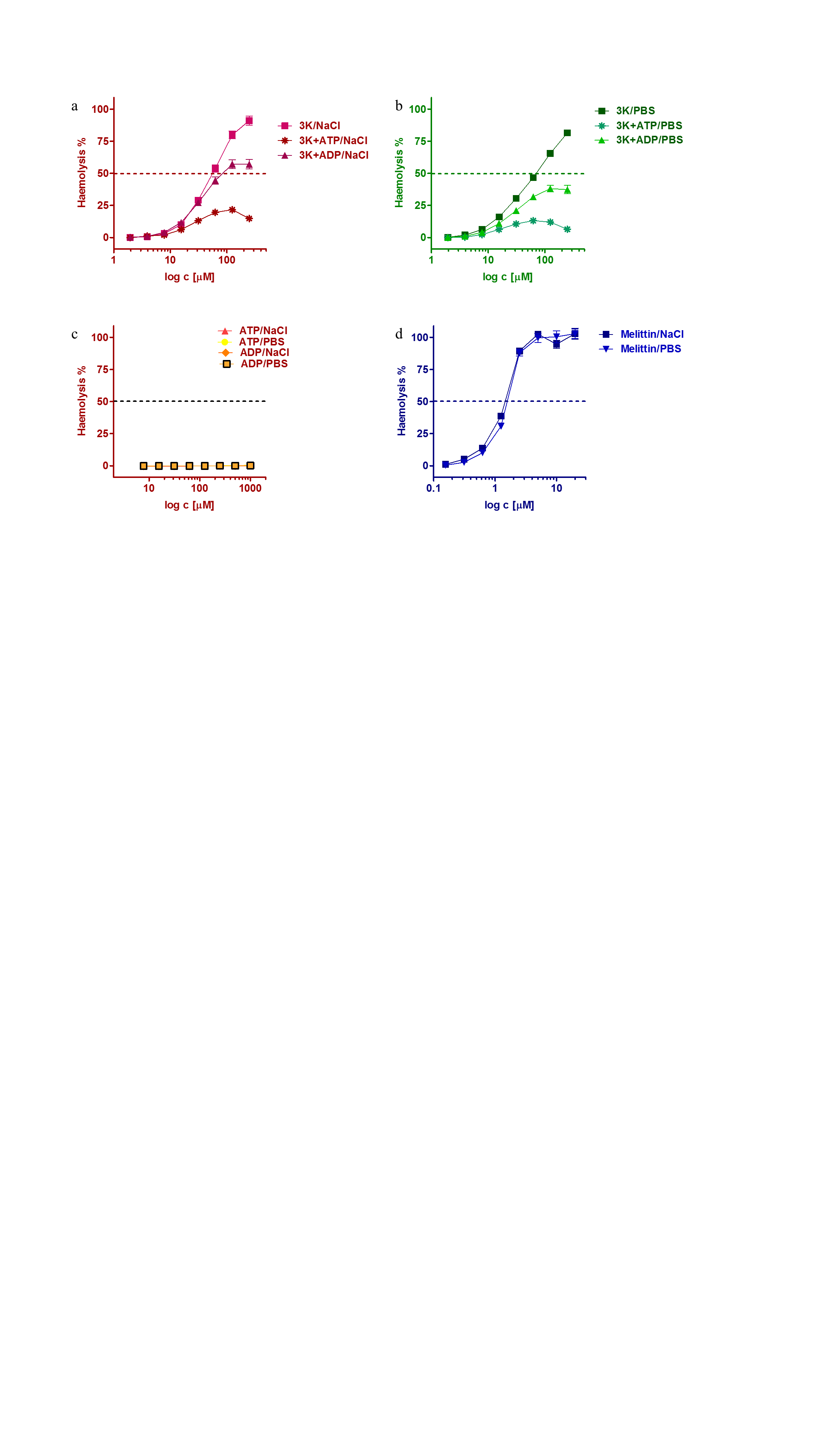
**

**Supplementary Figure 28. Hemolytic activity of 3K-AP in different ionic media with appropriate controls.** Hemolytic activity of 3K-ADP, 3K-ATP, and 3K a) In NaCl medium, b) In PBS. c) Negative controls with APs in both NaCl medium and PBS, d) Positive controls with melittin in both NaCl medium and PBS. All data presented as the mean ± SD (N = 4).

**Supplementary Table 5.** **Hemolytic activity of 3K-ATP, 3K-ADP, and 3K in NaCl and PBS media.** The half-maximal hemolysis concentration (HC_50_) values were calculated from the dose-response curves after fitting with non-linear regression. All data presented as the mean ± SD (N = 4).

| **Medium** | **Compound** | **HC_50_ [µM]** |
| --- | --- | --- |
| NaCl | 3K | 55.85 ± 1.79 |
|  | 3K-ADP | 112.90 ± 17.74 |
|  | 3K-ATP | > 250 |
| PBS | 3K | 69.11 ± 2.22 |
|  | 3K-ADP | > 250 |
|  | 3K-ATP | > 250 |

**Supplementary Figure 29. Cell viability of MonoMac-6 human monocytes after treatment with 3K-AP in PBS with appropriate controls.** Cytotoxic effect of 3K-ADP, 3K-ATP, ADP and ATP in PBS (pH = 7.4), after 2 hours of treatment. All data presented as the mean ± SD (N = 4).

**Supplementary Table 6.** **Cytotoxic effect of 3K-ATP and 3K-ADP in PBS medium.** The half-maximal inhibitory concentration (IC_50_) values were calculated from the dose-response curves after fitting with non-linear regression. All data presented as the mean ± SD (N = 4).

| **Compound** | **IC_50_ [µM]** |
| --- | --- |
| 3K-ADP | 63.54 ± 21.52 |
| 3K-ATP | > 250 |


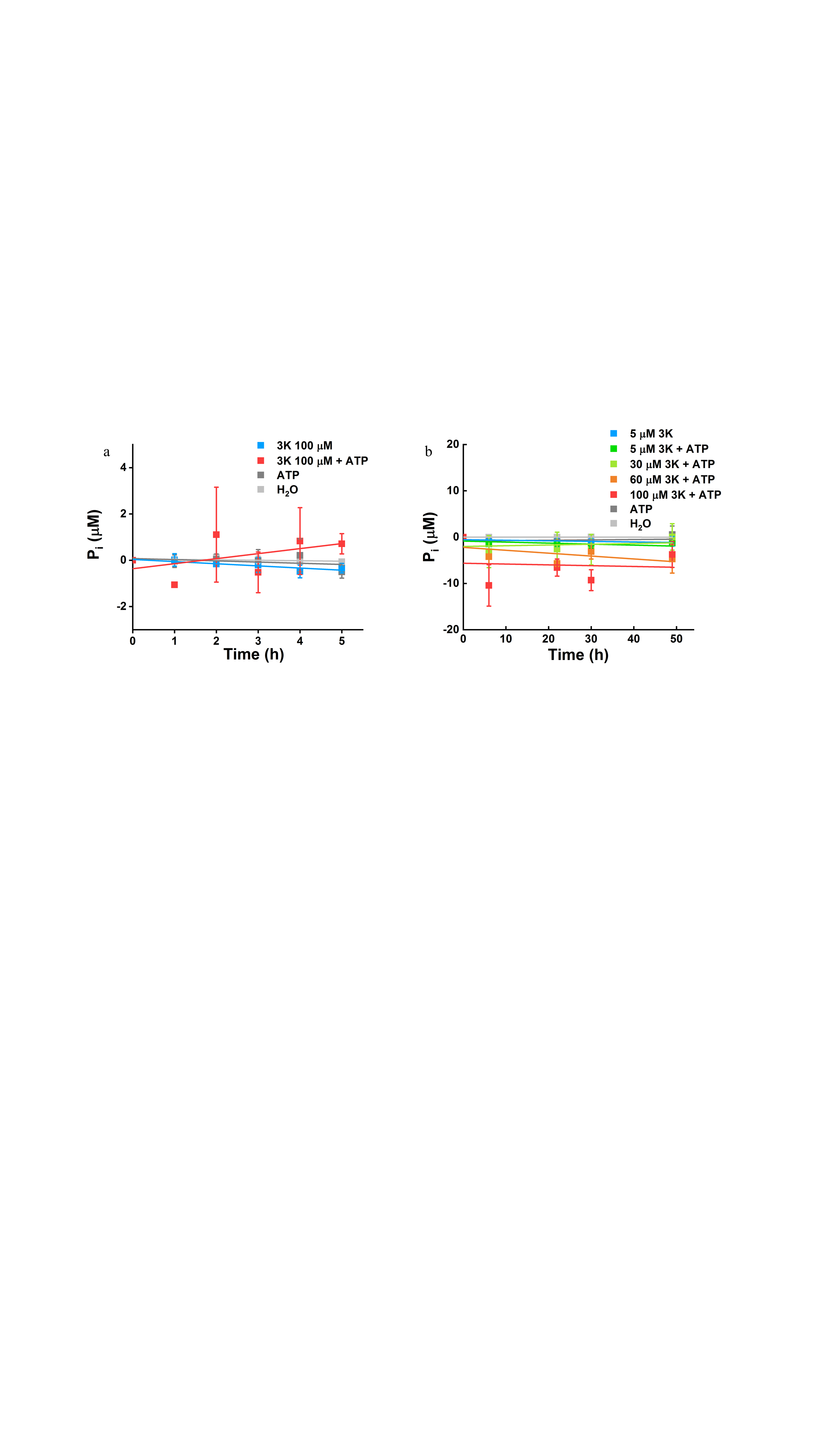


**Supplementary Figure 30.** **Kinetics of inorganic phosphate (Pi) release from ATP in the 3K-ATP co-assembly.** The phosphate (Pi) release from ATP was measured using Malachite Green Phosphate Assay Kit (MAK307, Sigma-Aldrich) at 25 ^o^C in a Tecan Infinite Nano+ plate reader instrument. ATP concentration was 250 µM in all ATP-containing samples. Means and standard errors are shown for *n* = 3 (left panel) or *n* = 2 (right panel). OD600 values, corrected for those at *t* = 0 for each sample, were converted to Pi concentration values based on standard curves recorded using the Pi standard provided with the kit. Linear regression analysis of the data revealed no statistically significant positive slopes (i.e., no measurable Pi production, *p* > 0.05 in all cases) for any of the obtained datasets.

**Comparison of the effects of ATP and phosphate ions from PBS upon addition to 3K**

We addressed whether competing assembly formation could be present. We earlier observed that lamellin-3K with phosphate ions from PBS (3K-P_i_) forms short rectangular lamellar structures^29^, that could compete with 3K-ATP mesh formation. Thus, we tested the assembly behaviour of 3K upon the addition of equimolar amounts of ATP and phosphate ions via PBS. Once mixed, the characteristic CD spectral peaks of 3K-ATP were obtained at 260 nm, whereas the 206 nm CD peak corresponding to 3K-P_i_ could not be observed (Supplementary Figure 31).^29^ Note, while 3K-ATP co-assembly forms almost spontaneously, for 3K-P_i_ a sonication period of 30 minutes is required, thus sonication was also applied, which resulted in no significant spectral changes. Upon addition of ATP to a sample with pre-formed 3K-P_i_ lamellae a sudden intensity decrease at 206 nm was observed, suggesting almost complete disassembly of 3K-P_i_. However, the 206 nm peak regained its intensity upon a further sonication step, thus somehow an equilibrium between the sonication-induced 3K-P_i_ and the spontaneously forming 3K-ATP could be achieved (Figure 4a). In general, results suggest a preference for 3K to ATP over phosphate ions.


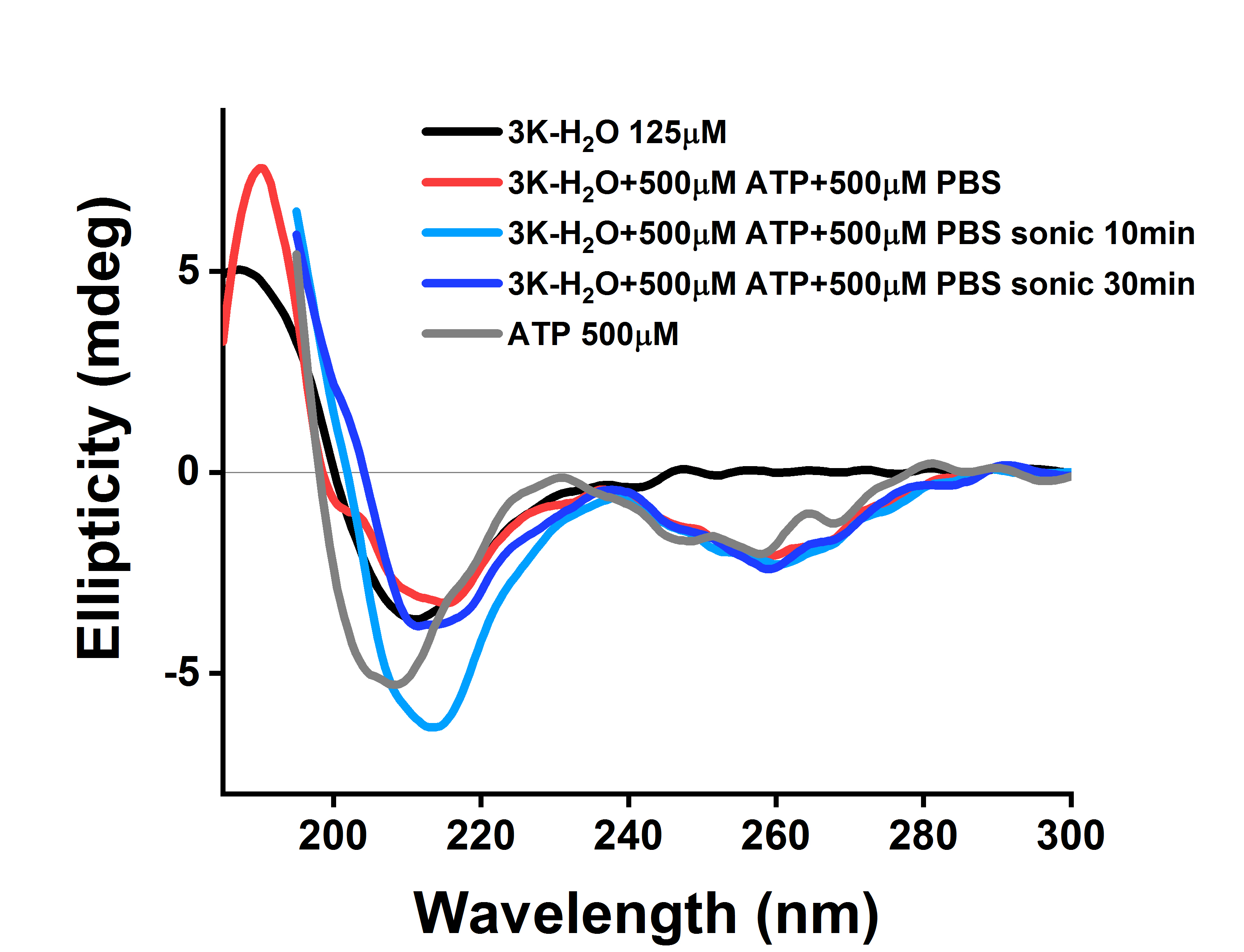


**Supplementary Figure 31. Comparison of the effects of ATP and phosphate ions from PBS on 3K upon simultaneous addition**. Addition of ATP and PBS, both at 500 μM concentration at the same time to 3K-H_2_O (125 μM) results in the characteristic contribution of ATP at 190 and 260 nm. After sonication, no appearance of 3K-PBS positive peak at 206 nm at any time frame indicating a preferential interaction of 3K with ATP and formation of 3K-ATP co-assembly predominantly.


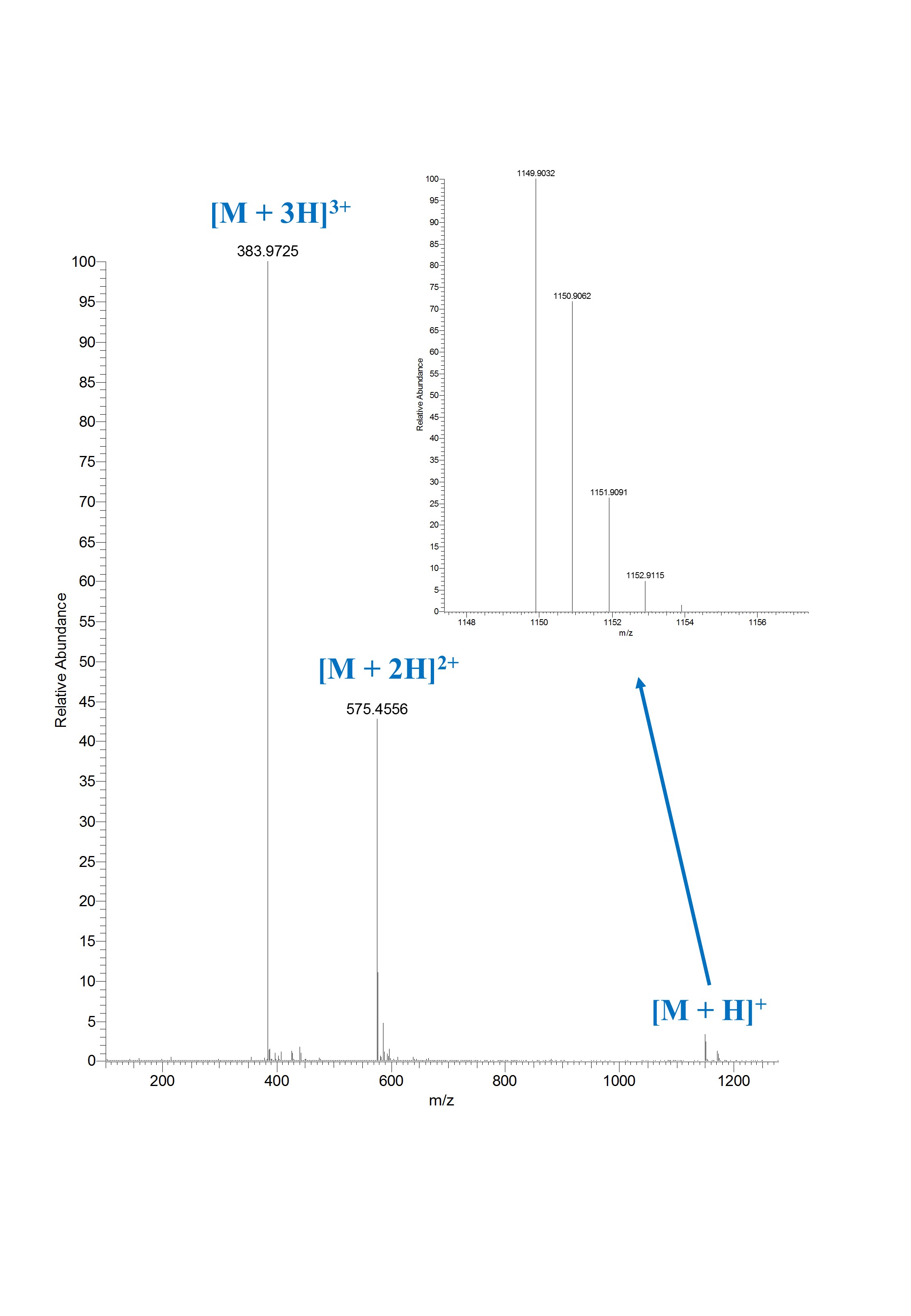


**Supplementary Figure 32. High resolution mass spectrum of 3K.** The observed monoisotopic molecular mass is 1148.8965. Calcd. M_mo_ = 1148.8988; Δppm = 2.00.

**References**

1. el Battioui, K. *et al.* In situ captured antibacterial action of membrane-incising peptide lamellae. *Nat. Commun.* **15**, 3424 (2024).

2. Trévisan, M. *et al.* Self-assembly and chiroptical properties in supramolecular complexes of adenosine phosphates and guanidinium-bispyrene. *Chirality* **30**, 719–729 (2018).

3. Ioannou, J. C., Donald, A. M. & Tromp, R. H. Characterising the secondary structure changes occurring in high density systems of BLG dissolved in aqueous pH 3 buffer. *Food Hydrocoll.* **46**, 216–225 (2015).

4. Avitabile, C. *et al.* Design, structural and functional characterization of a Temporin-1b analog active against Gram-negative bacteria. *Biochim. Biophys. Acta - Gen. Subj.* **1830**, 3767–3775 (2013).

5. Szigyártó, I. C. *et al.* Membrane active Janus-oligomers of β3-peptides. *Chem. Sci.* **11**, 6868–6881 (2020).

6. Martinek, T. A., Tóth, G. K., Vass, E., Hollósi, M. & Fülöp, F. cis-2-aminocyclopentanecarboxylic acid oligomers adopt a sheetlike structure: Switch from helix to nonpolar strand. *Angew. Chemie - Int. Ed.* **41**, 1718–1721 (2002).

7. Green, M., Taylor, R. & Wakefield, G. The synthesis of luminescent adenosine triphosphate passivated cadmium sulfide nanoparticles. *J. Mater. Chem.* **13**, 1859–1861 (2003).

8. Beć, K. B. *et al.* IR Spectra of Crystalline Nucleobases: Combination of Periodic Harmonic Calculations with Anharmonic Corrections Based on Finite Models. *J. Phys. Chem. B* **123**, 10001–10013 (2019).

9. Madzharova, F., Heiner, Z., Gühlke, M. & Kneipp, J. Surface-Enhanced Hyper-Raman Spectra of Adenine, Guanine, Cytosine, Thymine, and Uracil. *J. Phys. Chem. C* **120**, 15415–15423 (2016).

10. Tajmir-Riahi, H.-A. & Messaoudi, S. The Effects of Monovalent Cations Li+, Na+, K+, NH4 +, Rb+ and Cs+ on the Solid and Solution Structures of the Nucleic Acid Components. Metal Ion Binding and Sugar Conformation. *J. Biomol. Struct. Dyn.* **10**, 345–365 (1992).

11. Sit, I., Sagisaka, S. & Grassian, V. H. Nucleotide Adsorption on Iron(III) Oxide Nanoparticle Surfaces: Insights into Nano-Geo-Bio Interactions through Vibrational Spectroscopy. *Langmuir* **36**, 15501–15513 (2020).

12. Gasymov, O. K. & Glasgow, B. J. ANS fluorescence: Potential to augment the identification of the external binding sites of proteins. *Biochim. Biophys. Acta - Proteins Proteomics* **1774**, 403–411 (2007).

13. Daruka, L. *et al.* *ESKAPE pathogens rapidly develop resistance against antibiotics in development in vitro*. *Nature Microbiology* vol. 10 (2025).

14. Maharramov, E. *et al.* Exploring the principles behind antibiotics with limited resistance. *Nat. Commun.*  **16**, 1–18 (2025).

15. Riley, J. G. *et al.* The wbbD gene of E. coli strain VW187 (O7:K1) encodes a UDP-Gal: GlcNAcα-pyrophosphate-R β1,3-galactosyltransferase involved in the biosynthesis of O7-specific lipopolysaccharide. *Glycobiology* **15**, 605–613 (2005).

16. Sati, H. *et al.* The WHO Bacterial Priority Pathogens List 2024: a prioritisation study to guide research, development, and public health strategies against antimicrobial resistance. *Lancet Infect. Dis.* (2025) doi:10.1016/S1473-3099(25)00118-5.
